# Supplementary material for: Human Tumor-Associated Macrophage and Monocyte Transcriptional Landscapes Reveal Cancer-Specific Reprogramming, Biomarkers, and Therapeutic Targets
Source: Cancer Cell. 2019 Apr 15;35(4):588–602.e10. doi: 10.1016/j.ccell.2019.02.009 (PMC6472943; doi:10.1016/j.ccell.2019.02.009)
Supplement: Document S1. Figures S1–S6 [file mmc1.pdf]

Cancer Cell, Volume *35*

Supplemental Information

Human Tumor-Associated Macrophage and Monocyte Transcriptional Landscapes Reveal Cancer-Specific Reprogramming, Biomarkers, and Therapeutic Targets

Luca Cassetta, Stamatina Fragkogianni, Andrew H. Sims, Agnieszka Swierczak, Lesley M. Forrester, Hui Zhang, Daniel Y.H. Soong, Tiziana Cotechini, Pavana Anur, Elaine Y. Lin, Antonella Fidanza, Martha Lopez-Yrigoyen, Michael R. Millar, Alexandra Urman, Zhichao Ai, Paul T. Spellman, E. Shelley Hwang, J. Michael Dixon, Lisa Wiechmann, Lisa M. Coussens, Harriet O. Smith, and Jeffrey W. Pollard

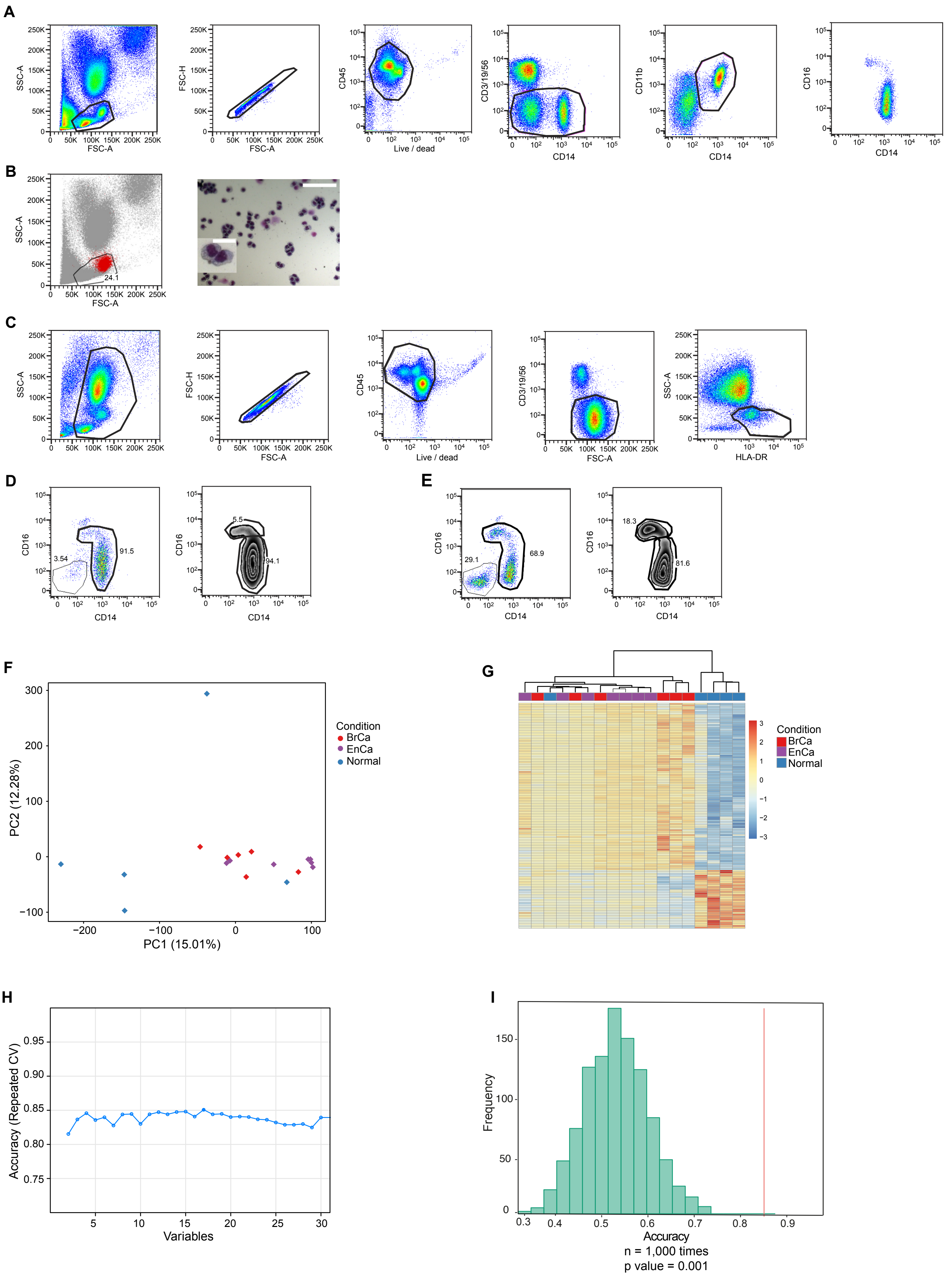

A
SSC-A
FSC-A
FSC-H
CD45
Live / dead
CD3/19/56
CD14
CD11b
CD16
B
24.1
C
HLA-DR
D
3.54
91.5
5.5
94.1
E
29.1
68.9
18.3
81.6
F
PC2 (12.28%)
PC1 (15.01%)
Condition
BrCa
EnCa
Normal
G
Condition
BrCa
EnCa
Normal
H
Accuracy (Repeated CV)
Variables
I
Frequency
Accuracy
n = 1,000 times
p value = 0.001

Figure S1. Flow cytometry gating strategy for the identification and isolation of human monocytes and transcriptomic analysis of the non-classical sub-population, related to Figure 1.

(A) Representative monocyte gating strategy based on physical and fluorescence parameters.

(B) Validation of gating strategy was performed by backgating and nuclei coloration (Giemsa, staining, scale bar 50 µm, inset 10 µm). Representantive monocytic cell shown.

(C, D and E) Representative monocyte gating strategy based on physical, and fluorescence parameters (C) of classical and non-classical monocytes separation in healthy controls (D) and cancer patients (E).

(F) PCA plot of n = 12,712 genes expressed in non-classical monocytes derived from healthy individuals (n = 6), breast cancer (BrCa) (n = 6) and endometrial cancer (EnCa) patients (n = 7).

(G) Hierarchical clustering on all samples (Healthy, n = 5; BrCa, n = 6; EnCa, n = 7) using the significantly DEGs between breast and healthy non-classical monocytes (n = 139 genes). Expression values are Z score-transformed and samples clustered using complete linkage and Euclidean distance.

(H) Plot of the accuracy yielded for different gene signature sizes during feature selection with Recursive feature elimination with Random forest (RFE-RF) model training.

(I) Histogram showing the performance of random classifiers during Random forest (RF) model training. Solid vertical red line represents the performance of the observed 17-gene signature on the training data.

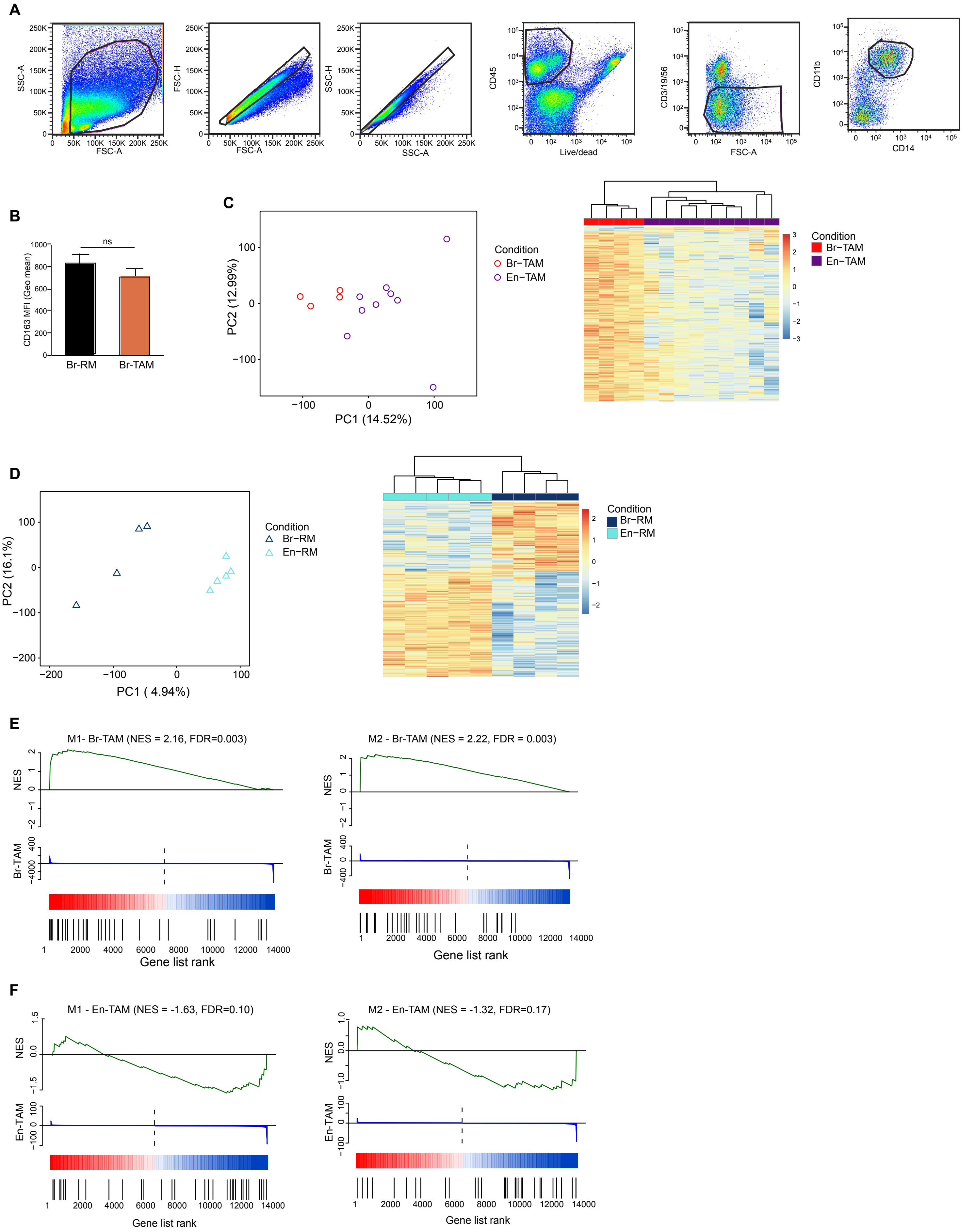
A
SSC-A
FSC-A
FSC-H
SSC-H
CD45
Live/dead
CD3/19/56
CD11b
CD14
B
ns
CD163 MFI (Geo mean)
Br-RM
Br-TAM
C
PC2 (12.99%)
PC1 (14.52%)
Condition
Br−TAM
En−TAM
D
PC2 (16.1%)
PC1 (4.94%)
Condition
Br−RM
En−RM
E
M1- Br-TAM (NES = 2.16, FDR=0.003)
M2 - Br-TAM (NES = 2.22, FDR = 0.003)
NES
Br-TAM
Gene list rank
F
M1 - En-TAM (NES = -1.63, FDR=0.10)
M2 - En-TAM (NES = -1.32, FDR=0.17)
NES
En-TAM
Gene list rank

Figure S2. Flow cytometry sorting strategy of tissue macrophages and TAMs, and analysis of CD163 expression and distinct macrophage populations, related to Figure 2.

(A) Gating strategy for tissue macrophages and TAMs; macrophages were defined as $CD45^{+}CD3/56/19^{-}CD11b^{+}CD14^{+}CD163^{+}$.

(B) Representative histogram of macrophage CD163 expression in Br-MR (n = 5) compared to Br-TAM (n = 5). Data are expressed as Geometric Mean (Mean±SEM).

(C) PCA plot of n = 14,229 expressed genes in Br-TAM (n = 4) and En-TAM (n = 9) (left). Hierarchical clustering of all DEGs between Br-TAM and En-TAM (right). Expression values are Z score-transformed and samples clustered using complete linkage and Euclidean distance.

(D) PCA plot of n = 13,907 expressed genes in Br-RM (n = 4) and En-RM (n = 5) (left). Hierarchical clustering of all DEGs between Br-RM against En-RM (right).Expression values are Z score-transformed and samples clustered using complete linkage and Euclidean distance.

(E) Enrichment analysis of M1-like (left) and M2-like (right) macrophage signature (Martinez et al., 2006) in Br-TAM. Black bars represent the position of M1-like or M2-like genes in the ranked list of Br-TAM expressed genes together with the running enrichment score (green line).

(F) Enrichment analysis of M1-like (left) and M2-like (right) macrophage signature (Martinez et al., 2006) in En-TAM. Black bars represent the position of M1-like or M2-like genes in the ranked list of En-TAM expressed genes together with the running enrichment score (green line).

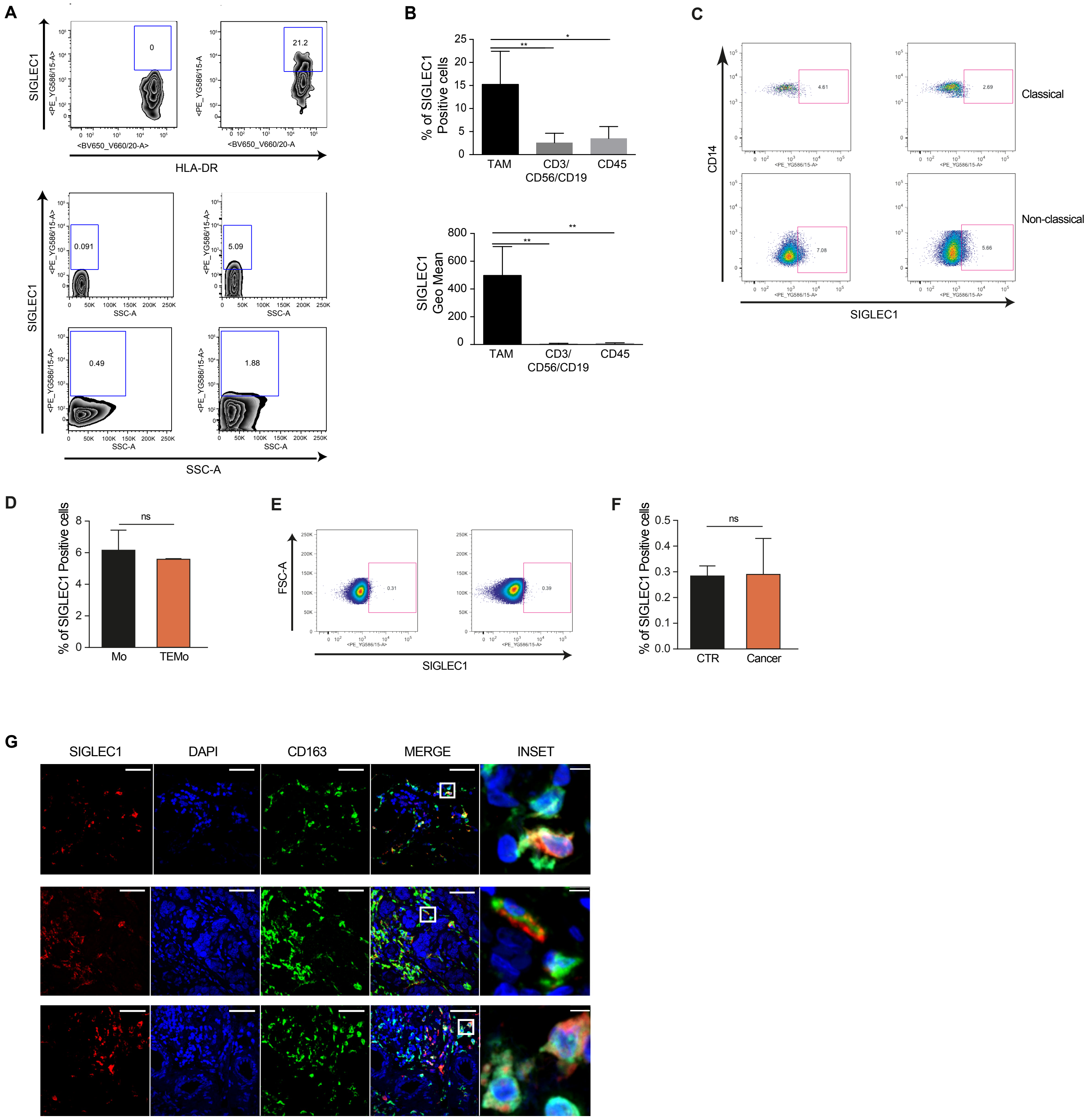
A
B
C
D
E
F
G
SIGLEC1
HLA-DR
SSC-A
% of SIGLEC1 Positive cells
SIGLEC1 Geo Mean
TAM
CD3/ CD56/CD19
CD45
CD14
Classical
Non-classical
Mo
TEMo
ns
FSC-A
CTR
Cancer
DAPI
CD163
MERGE
INSET

Figure S3. Expression of SIGLEC1 in Br-TAM and cell types, related to Figure 4.

(A) SIGLEC1 expression in Br-TAM samples (top), in $CD3^{+}/19^{+}/56^{+}$ cells (middle) and $CD45^{-}$ cells (bottom) from breast tumors. FMO controls (left), SIGLEC1 staining (right). Representative plot of 3 independent experiments is shown.

(B) Quantification of $SIGLEC1^{+}$ cells in Br-TAM, $CD3^{+}/19^{+}/56^{+}$ cells and $CD45^{-}$ cells from breast tumors (n = 3). Data are depicted as % of $SIGLEC1^{+}$ cells (top) and Geo Mean values (bottom).

(C) SIGLEC1 expression in classical and non-classical Mo and breast cancer TEMo (square gate based on FMO control). Representative plot of 3 independent experiments is shown.

(D) Quantification of % of $SIGLEC1^{+}$ cells in Mo and TEMo (n = 3). Data are depicted as % of SIGLEC1+ cells.

(E) SIGLEC1 expression in blood circulating granulocytes from healthy donors (CTR) and breast cancer patients (gated on live $CD45^{+}$ SSC high cells, square gate based on FMO control). Representative plot of 3 independent experiments is shown.

(F) Quantification of % of $SIGLEC1^{+}$ cells in blood circulating granulocytes from healthy donors (CTR) and breast cancer patients (n = 3).

(G) CD163 and SIGLEC1 immunofluorescent staining on breast cancer tissue samples (n = 5 each, Bars = 50 µm, inset = 5 µm, 3 representative samples are shown). Enlargement of the selected area showing a representative $SIGLEC1^{+}CD163^{+}$ macrophage.

ns = not significant, *p < 0.01, **p < 0.001; (B) One-way ANOVA, (D,F) Student's t-test, (B, D ,F) Data depicted as Mean±SEM.

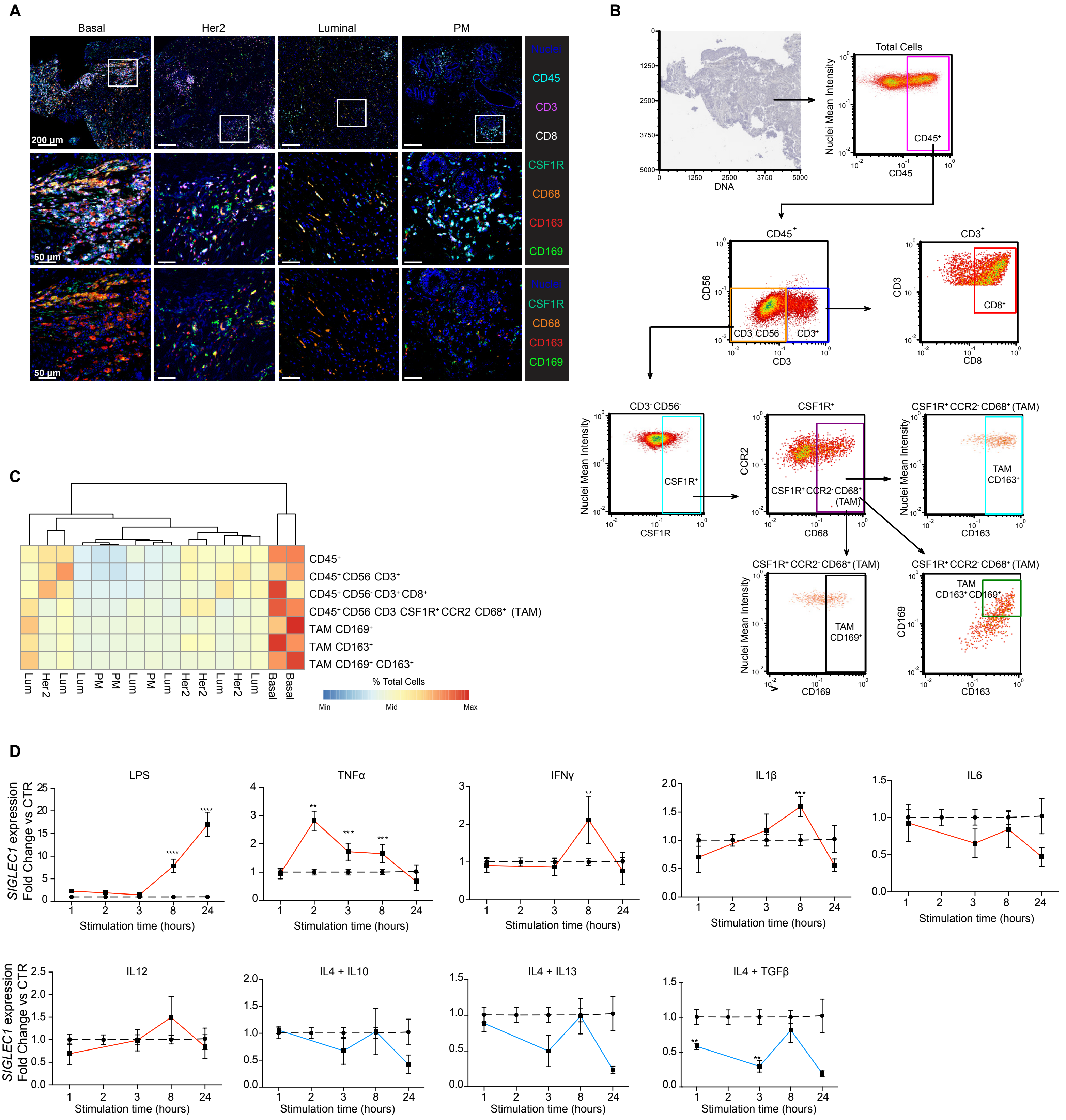
A
Basal
Her2
Luminal
PM
200 µm
50 µm
Nuclei
CD45
CD3
CD8
CSF1R
CD68
CD163
CD169
B
DNA
Total Cells
Nuclei Mean Intensity
CD45
CD45+
CD56
CD3
CD3- CD56-
CD3+
CD8
CD8+
CSF1R
CSF1R+
CCR2
CD68
CSF1R+ CCR2- CD68+ (TAM)
TAM CD163+
TAM CD169+
CD169
CD163
TAM CD163+ CD169+
C
CD45+
CD45+ CD56- CD3+
CD45+ CD56- CD3+ CD8+
CD45+ CD56- CD3- CSF1R+ CCR2- CD68+ (TAM)
TAM CD169+
TAM CD163+
TAM CD169+ CD163+
Lum
Her2
PM
Basal
% Total Cells
Min
Mid
Max
D
LPS
TNFα
IFNγ
IL1β
IL6
IL12
IL4 + IL10
IL4 + IL13
IL4 + TGFβ
SIGLEC1 expression Fold Change vs CTR
Stimulation time (hours)

Figure S4. Multiplex immunohistochemistry analysis of different breast cancer subtypes and SIGLEC1 regulation by cytokines, related to Figure 4.

(A) Representative micrographs reflecting pseudo-colored images following multiplex IHC of cell populations across breast cancer subtypes as indicated. Boxed insets are depicted at higher magnification in corresponding columns. Scale bars as indicated ($n = 16$).

(B) Image cytometry plots of quantitative multiplex immunohistochemistry on tissue biopsies. Cumulative cell populations from total tissue areas were normalized to total cell number. One representative sample out of 16 is shown.

(C) A heatmap of each cell population as a percent of total cells is shown with a dendrogram of unsupervised hierarchical clustering, scaled by row and using correlation as a distance measure, and average as a clustering method. Each column represents an independent tumor according to sub-type. (Lum: luminal) and prophylactic mastectomy/mammoplasty (PM) samples, ($n = 16$).

(D) *SIGLEC1* mRNA expression in PMA-treated THP1 cells stimulated with pro- (red) or anti-inflammatory (blue) cytokines as indicated. LPS acts as a positive pro-inflammatory signal control. Colored bar indicates *SIGLEC1* expression in cytokine-treated samples, dotted black line indicates *SIGLEC1* expression in control PBS-treated samples. Data are depicted as fold change vs CTR ($n = 3$, $**p < 0.001$, $***p<0.0001$, $****p<0.00001$; Student's t-test, Mean ± SEM).

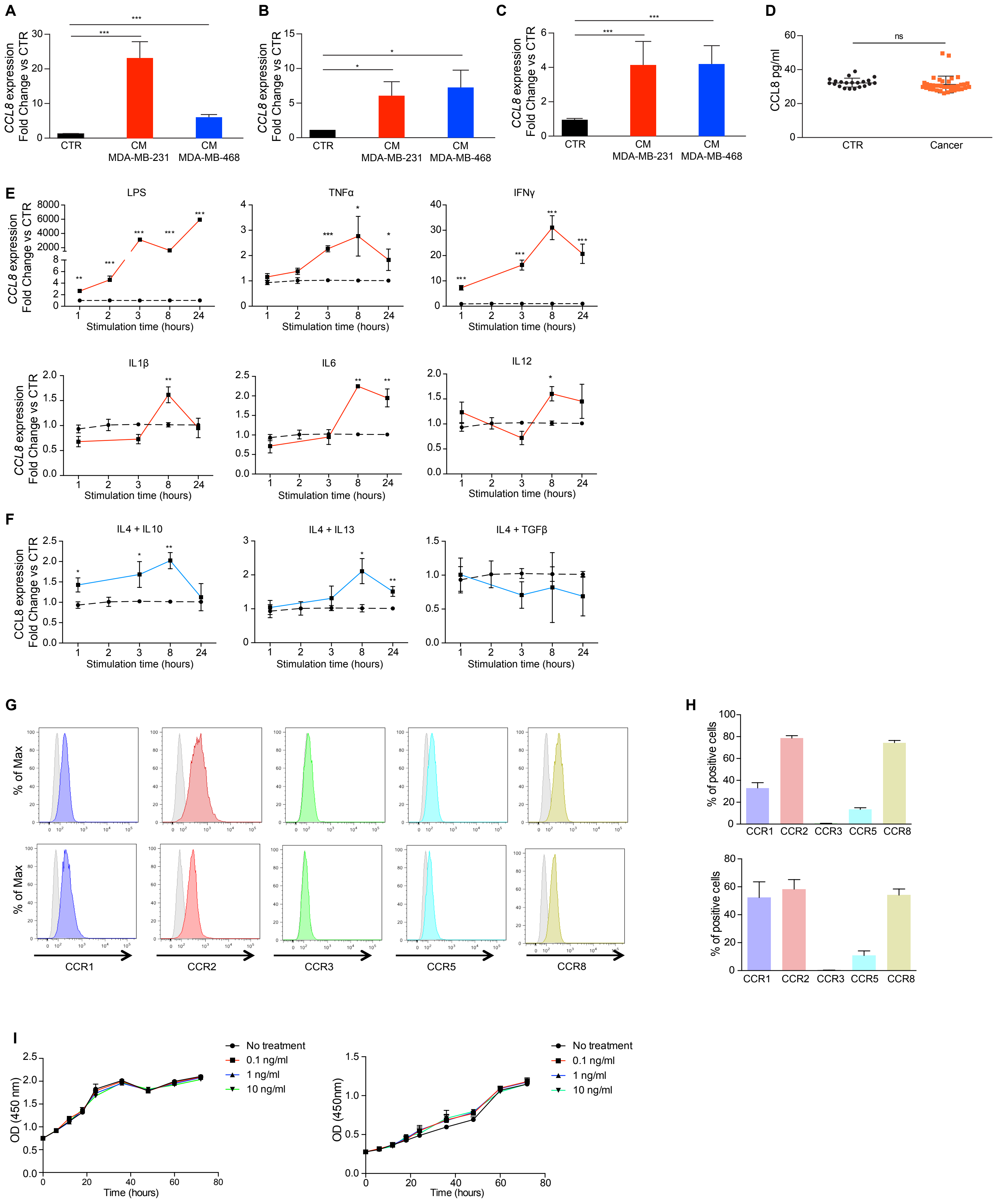

A
B
C
D
CCL8 expression Fold Change vs CTR
CTR
CM MDA-MB-231
CM MDA-MB-468
***
*
ns
CCL8 pg/ml
Cancer
E
LPS
TNFα
IFNγ
IL1β
IL6
IL12
Stimulation time (hours)
F
IL4 + IL10
IL4 + IL13
IL4 + TGFβ
G
% of Max
CCR1
CCR2
CCR3
CCR5
CCR8
H
% of positive cells
I
No treatment
0.1 ng/ml
1 ng/ml
10 ng/ml
OD (450 nm)
OD (450nm)
Time (hours)

Figure S5. Expression of SIGLEC1 and CCL8 in macrophages after stimulation with cancer cell conditioned medium or cytokines, related to Figure 5.

(A, B and C) *CCL8* mRNA expression in PMA-treated THP1 cells (A), primary MDM (B), and iPSDM (C) stimulated for 24 hr with culture medium (CTR), MDA-MB-231 CM or MDA-MB-468 CM. Data are depicted as fold change vs CTR (n = 3).

(D) ELISA for CCL8 in serum of healthy donors (n = 21) and breast cancer patients (n = 38). Data are depicted as pg/ml.

(E and F) *CCL8* mRNA expression in PMA-treated THP1 cells stimulated for times shown with pro- (E) or anti-inflammatory (F) cytokines as shown. Colored line indicated cytokine-treated samples, dotted black line PBS-treated samples; Data are depicted as fold change vs CTR (n = 3).

(G) Representative histograms of CCR1, CCR2, CCR3, CCR5 and CCR8 expression in MDA-MB-231 (top) and MDA-MB-468 (bottom) cells. Grey histograms indicate unstained samples. Representative plot of 3 independent experiments is shown.

(H) Percentage of CCR1, CCR2, , CCR3, CCR5 and CCR8 positive cells in total MDA-MB-231 (top) and MDA-MB-468 cells (bottom), (n = 3).

(I) MDA-MB-231 (left) and MDA-MB-468 (right) proliferation assay in the presence of PBS (No treatment), 0.1ng/ml, 1ng/ml and 10ng/ml of CCL8 from 0-80 hr. No statistical differences between treatments and controls (n = 3).

ns = not significant, $p < 0.01$, *$p < 0.001$, $p<0.0001$, ***$p<0.00001$; (A-C) One-way ANOVA, (D, E, F, I) Student's t-test, (A, B, C, E, F, H, I) Data depicted as Mean±SEM, (D) Horizontal bars represent the mean of the individual values±SD.

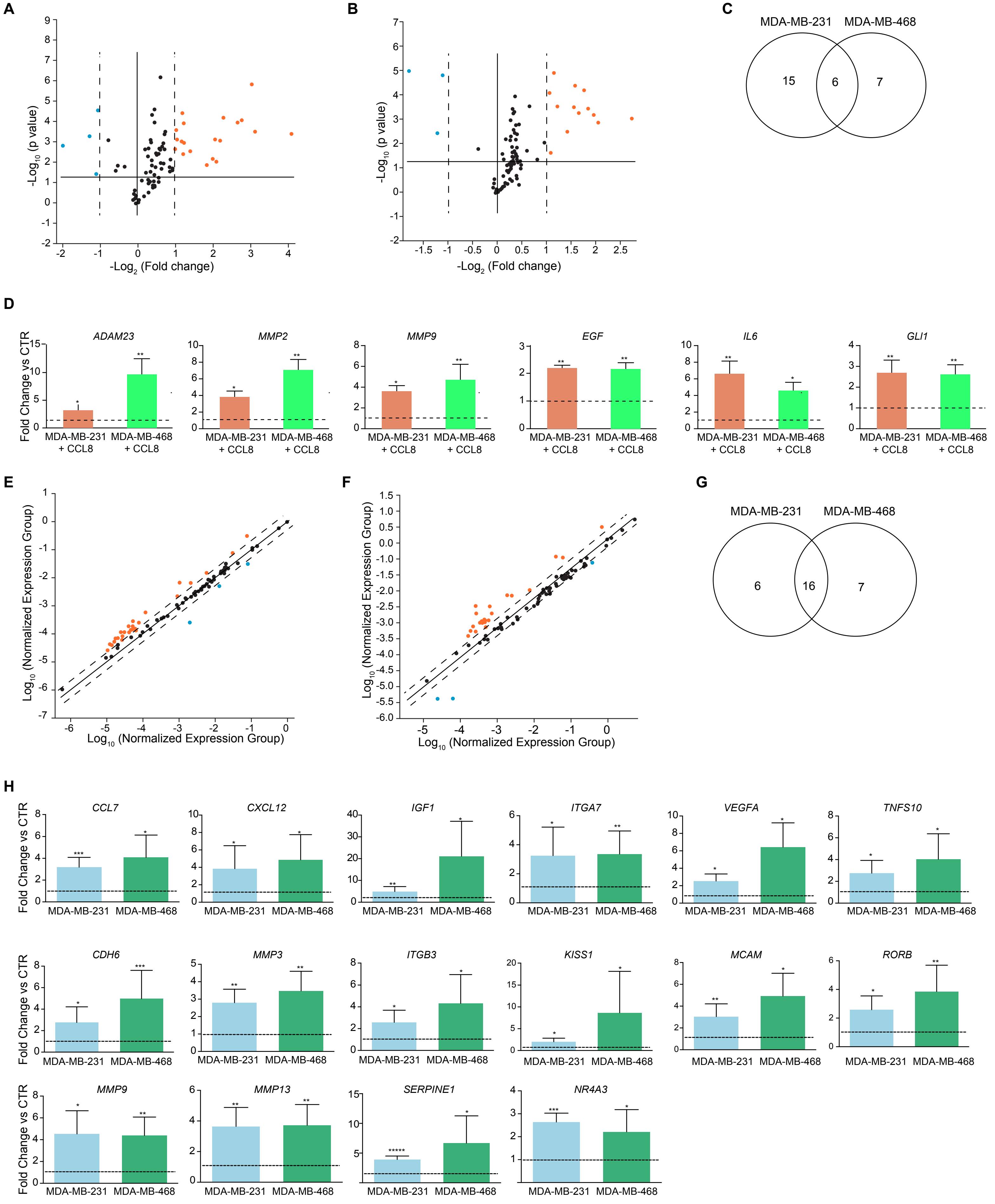
A
B
C
MDA-MB-231
MDA-MB-468
15
6
7
-Log10 (p value)
-Log2 (Fold change)
D
Fold Change vs CTR
ADAM23
MMP2
MMP9
EGF
IL6
GLI1
MDA-MB-231 + CCL8
MDA-MB-468 + CCL8
E
F
G
Log10 (Normalized Expression Group)
6
16
7
H
CCL7
CXCL12
IGF1
ITGA7
VEGFA
TNFS10
CDH6
MMP3
ITGB3
KISS1
MCAM
RORB
MMP9
MMP13
SERPINE1
NR4A3
MDA-MB-231
MDA-MB-468

Figure S6. Breast cancer qPCR array on cancer cells stimulated with rCCL8 and macrophage conditioned medium, related to Figure 5.

(A and B) Volcano plot showing genes whose expression is significantly (Log_2FC +/- 1, $p < 0.05$) downregulated (blue dots) and upregulated (orange dots) in MDA-MB-231 (A) or MDA-MB-468 (B) cells after incubation with 1ng/ml of rCCL8 for 16 hr (n = 3).

(C) Venn diagram of commonly upregulated genes between MDA-MB-231 (left circle) and MDA-MB-468 (right circle) after rCCL8 treatment.

(D) mRNA expression of 6 commonly upregulated genes in MDA-MB-231 or MDA-MB-468 after CCL8 stimulation. Dotted black line represents normalized expression level in untreated control samples. Data are depicted as fold change vs CTR (n = 3).

(E and F) Volcano plot showing normalised expression (Log_{10}) of cells after incubation with CM primed MDM supernatant for 16 hr (n = 3) in MDA-MB-231 (E) and MDA-MB-468 (F). Dots represent genes whose expression is significantly ($Log_{10}FC$ +/- 2, $p < 0.05$) downregulated (blue dots) and upregulated (orange dots).

(G) Venn diagram of commonly upregulated genes between MDA-MB-231 (left circle) and MDA-MB-468 (right circle).

(H) mRNA expression of 16 commonly upregulated genes in MDA-MB-231 or MDA-MB-468 after CM primed MDM supernatant stimulation; dotted black line represents normalized expression level in untreated control samples (n = 3).

*p < 0.01, **p < 0.001, ***p < 0.0001, *****p < 0.000001; (D, H) Student's t-test, (D, H) Data depicted as Mean±SEM.
