# Supplementary material for: Human Tumor-Associated Macrophage and Monocyte Transcriptional Landscapes Reveal Cancer-Specific Reprogramming, Biomarkers, and Therapeutic Targets
Source: Cancer Cell. 2019 Apr 15;35(4):588–602.e10. doi: 10.1016/j.ccell.2019.02.009 (PMC6472943; doi:10.1016/j.ccell.2019.02.009)
Supplement: Document S2. Article plus Supplemental Information [file mmc8.pdf]

Cancer Cell

Article

Human Tumor-Associated Macrophage and Monocyte Transcriptional Landscapes Reveal Cancer-Specific Reprogramming, Biomarkers, and Therapeutic Targets

Graphical Abstract

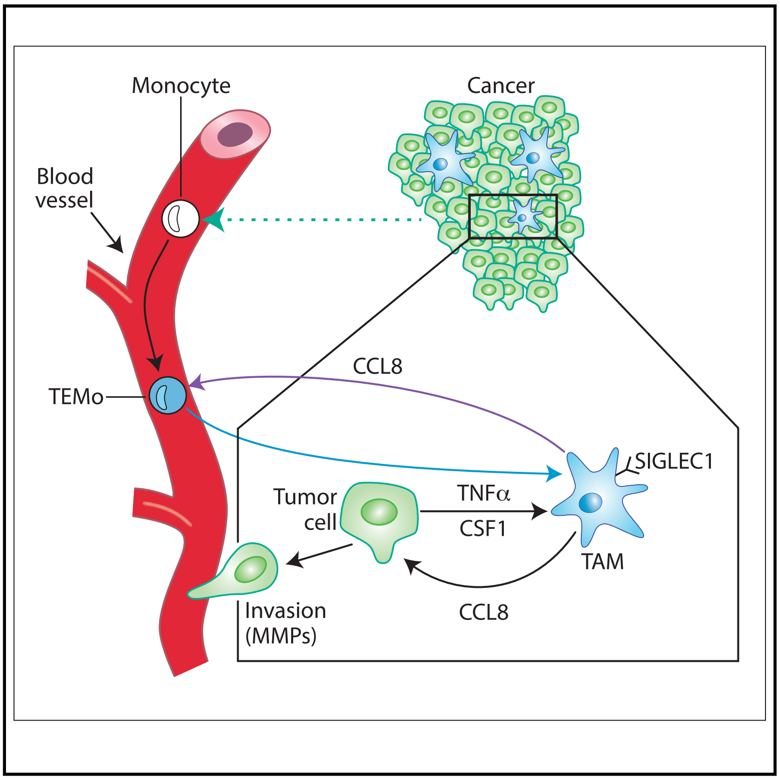

Highlights

- Cancer alters circulating monocytic populations and their transcriptomes
- Tumor-associated macrophages show tissue-specific programming
- Tumor-associated macrophages' gene signature correlates with poor clinical outcome
- Tumor-associated macrophages enhance cancer cells malignancy through CCL8

Authors

Luca Cassetta, Stamatina Fragkogianni, Andrew H. Sims, ..., Lisa M. Coussens, Harriet O. Smith, Jeffrey W. Pollard

Correspondence

jeff.pollard@ed.ac.uk

In Brief

Cassetta et al. identify a breast cancer tumor-associated macrophage (TAM) transcriptome that is different from those of monocytes and tissue-resident macrophages, and which is associated with shorter disease-specific survival, and they demonstrate crosstalk between tumor cells and TAMs via SIGLEC1, CCL8, and CSF1.

Cassetta et al., 2019, Cancer Cell *35*, 588–602
April 15, 2019 © 2019 The Author(s). Published by Elsevier Inc.
https://doi.org/10.1016/j.ccell.2019.02.009

Cancer Cell
Article

Human Tumor-Associated Macrophage and Monocyte Transcriptional Landscapes Reveal Cancer-Specific Reprogramming, Biomarkers, and Therapeutic Targets

Luca Cassetta,[1,12] Stamatina Fragkogianni,[1,12] Andrew H. Sims,[2] Agnieszka Swierczak,[1] Lesley M. Forrester,[4] Hui Zhang,[5] Daniel Y.H. Soong,[1] Tiziana Cotechini,[3] Pavana Anur,[10] Elaine Y. Lin,[3] Antonella Fidanza,[4] Martha Lopez-Yrigoyen,[4] Michael R. Millar,[1,9] Alexandra Urman,[7] Zhichao Ai,[1] Paul T. Spellman,[10] E. Shelley Hwang,[11] J. Michael Dixon,[6] Lisa Wiechmann,[7] Lisa M. Coussens,[3] Harriet O. Smith,[8] and Jeffrey W. Pollard[1,5,13,*]

[1]MRC Centre for Reproductive Health, Queen's Medical Research Institute, The University of Edinburgh, Edinburgh EH16 4TJ, UK
[2]Applied Bioinformatics of Cancer, University of Edinburgh Cancer Research Centre, Institute of Genetics and Molecular Medicine, Edinburgh EH4 2XR, UK
[3]Department of Cell, Developmental & Cancer Biology, and Knight Cancer Institute, Oregon Health & Science University, Portland 97239, USA
[4]MRC Centre for Regenerative Medicine, University of Edinburgh, Edinburgh EH16 4UU, UK
[5]Department of Developmental and Molecular Biology, Albert Einstein College of Medicine, New York 10461, USA
[6]Edinburgh Breast Unit and Breast Cancer Now Research Unit, University of Edinburgh, Edinburgh EH4 2XU, UK
[7]Department of Surgery, Montefiore Medical College, New York 10467, USA
[8]Department of Obstetrics and Gynecology, Albert Einstein College of Medicine and Montefiore Medical Center, New York 10461, USA
[9]Aquila Biomedical, Edinburgh Bioquarter, Little France Road, Edinburgh EH16 4TJ, UK
[10]Department of Molecular and Medical Genetics and Knight Cancer Institute, Oregon Health & Science University, Portland 97239, USA
[11]Department of Surgery, Duke University Medical Center, Durham, NC 27710, USA
[12]These authors contributed equally
[13]Lead Contact
*Correspondence: jeff.pollard@ed.ac.uk
https://doi.org/10.1016/j.ccell.2019.02.009

SUMMARY

The roles of tumor-associated macrophages (TAMs) and circulating monocytes in human cancer are poorly understood. Here, we show that monocyte subpopulation distribution and transcriptomes are significantly altered by the presence of endometrial and breast cancer. Furthermore, TAMs from endometrial and breast cancers are transcriptionally distinct from monocytes and their respective tissue-resident macrophages. We identified a breast TAM signature that is highly enriched in aggressive breast cancer subtypes and associated with shorter disease-specific survival. We also identified an auto-regulatory loop between TAMs and cancer cells driven by tumor necrosis factor alpha involving SIGLEC1 and CCL8, which is self-reinforcing through the production of CSF1. Together these data provide direct evidence that monocyte and macrophage transcriptional landscapes are perturbed by cancer, reflecting patient outcomes.

INTRODUCTION

Tumors evolve as ecosystems consisting of tumor, stromal, and infiltrating immune cells. Macrophages are major components of this ecosystem. In mouse models, different subpopulations of tumor-associated macrophages (TAMs) promote angiogenesis, tumor cell invasion, intravasation, and, at the metastatic site, tumor cell extravasation and persistent growth, and suppress cytolytic T cell responses (Cassetta and Pollard, 2018). In homeostasis, tissue macrophages have different

Significance

Human breast and endometrial cancer systemically alter circulating monocytes and, from these cells, a transcriptional signature recognizing cancer was determined. Cancer locally educates tumor-associated macrophages (TAMs) such that they are different from monocytes, from tissue-resident macrophages, and from each other. A "cancer-specific immune signature" derived from breast cancer TAMs is prognostic for poor disease-specific survival in publically available datasets from whole tumors. Breast cancer TAMs express CCL8, which is chemotactic for monocytes and drives a positive regulatory loop between cancer cells and TAMs through CSF1 and TNF-α, which upregulates SIGLEC1. *SIGLEC1* and *CCL8* expression together are independent prognostic markers for poor survival. These data suggest that cancer-specific targeting of TAMs could be of therapeutic benefit.

 © 2019 The Author(s). Published by Elsevier Inc.
This is an open access article under the CC BY license (http://creativecommons.org/licenses/by/4.0/).

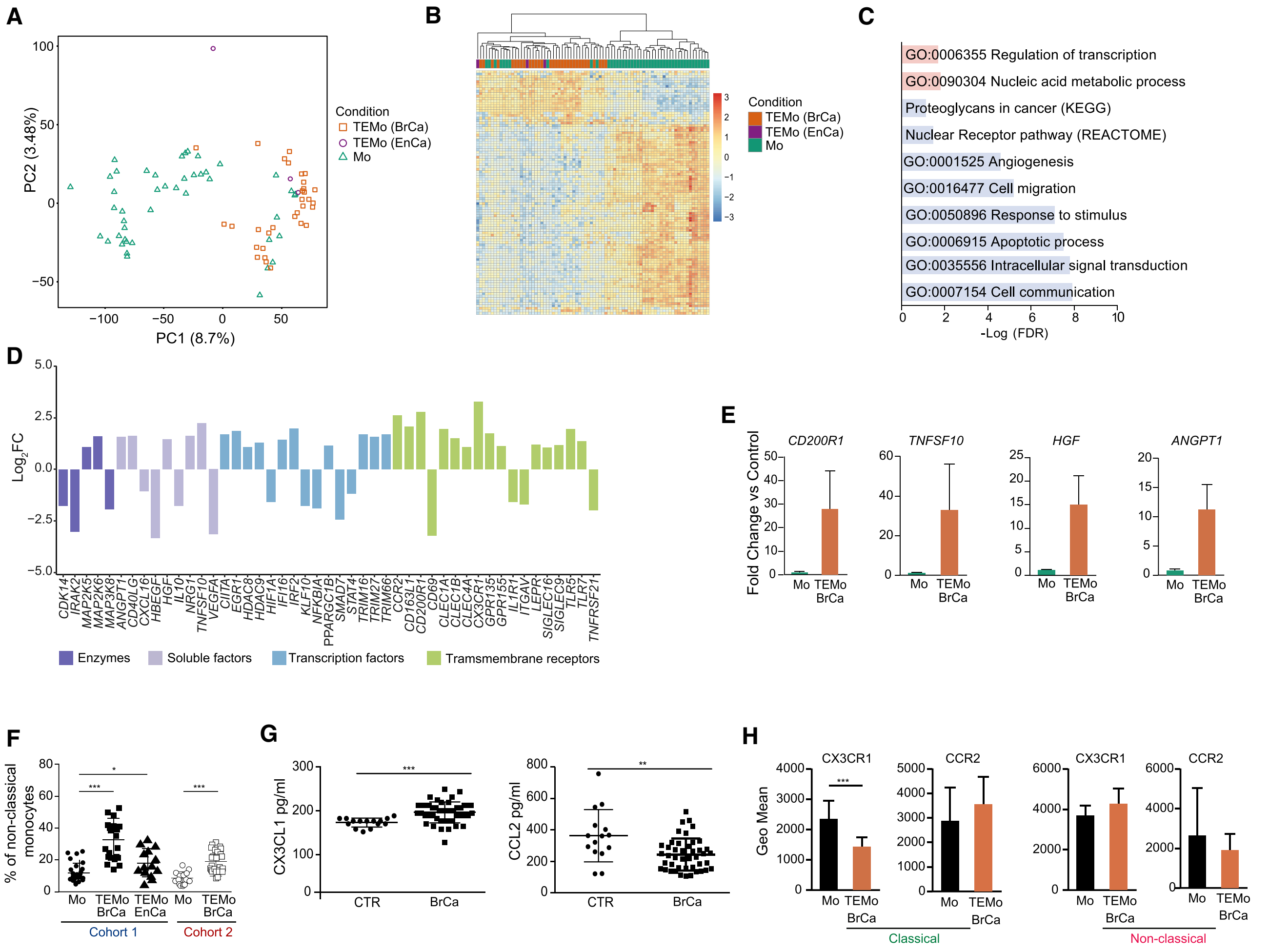

I

n = 22	Reference: Cancer n = 9	Reference: Healthy n = 13
Prediction: Cancer	9	4
Prediction: Healthy	0	9

J

RFE-RF 17 genes	Testing set n = 22
Accuracy	82%
Sensitivity	100%
Specificity	69%
Positive Predicted Value (PPV)	69%
Negative Predicted Value (NPV)	100%

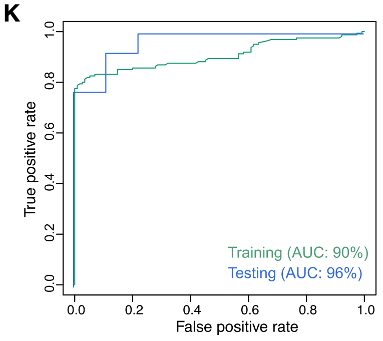

Figure 1. Cancer Alters the Transcriptome of Human Monocytes

(A) Principal-component analysis (PCA) plot of n = 12,157 genes expressed in monocytes from healthy individuals (Mo) (n = 45) and TEMo from cancer patients (n = 35; breast cancer [BrCa] = 32; endometrial cancer [EnCa] = 3).

(B) Hierarchical clustering of all differentially expressed genes (DEGs) between Mo and TEMo. Expression values are *Z* score transformed. Samples were clustered using complete linkage and Euclidean distance.

(C) Gene ontology (GO) analysis of DEGs between TEMo and Mo (blue, downregulated genes; red, upregulated genes).

(D) Bar plot of selected DEGs in TEMo (FDR <= 0.05).

(E) Expression of *CD200R1*, *TNFSF10*, *HGF*, and *ANGPT1* mRNA in Mo and breast TEMo (n = 3–5; independent from the RNA-seq cohort).

(F) Relative distribution of non-classical monocytes from healthy controls and BrCa and EnCa patients determined by flow cytometry shown as percentage in the monocyte gate. Cohort 1: Mo, n = 31, BrCa TEMo, n = 22, EnCa TEMo, n = 12. Cohort 2, BrCa and controls only: Mo, n = 18, TEMo, n = 33.

(G) ELISA quantification of CX3CL1 and CCL2 levels in the sera of control (CTR) (n = 15) and BrCa patients (n = 45).

(H) Expression of CX3CR1 and CCR2 in Mo (n = 10) and breast TEMo (n = 31). Data are expressed as geometric mean (Geo mean).

(legend continued on next page)

origins; however, in most cancer models, TAMs are recruited from bone marrow progenitors known as monocytes (Arwert et al., 2018; Franklin et al., 2014; Qian et al., 2011). These monocytes are termed classical (human $CD14^{++}CD16^{-}$ and mouse $CD11b^{+}Ly6C^{+}$) and non-classical (human $CD14^{+}CD16^{+}$; mouse $CD11b^{+}Ly6C^{-}$). The classical population is recruited as the tumor progresses and differentiates *in situ* to TAMs, often via a CCL2-CCR2 chemokine signaling pathway. Inhibition of CCR2 signaling blocks TAM recruitment and thus inhibits tumor cell seeding and persistent growth, improving the survival of mice (Qian et al., 2011).

The pro-tumoral behavior of monocytes and TAMs in mouse models has made them attractive therapeutic targets. Targeting strategies include inhibiting monocyte recruitment, depletion of TAMs, and functional/phenotypic reprogramming (Cassetta and Pollard, 2018). These therapies, however, are limited by the lack of TAM-specific markers (Williams et al., 2016), as well as our limited understanding of their functions in human cancers (Takeya and Komohara, 2016). We hypothesize that human breast and endometrial cancer will have a significant impact on circulating monocytes and their progeny TAMs, which will indicate signaling pathways, therapeutic and diagnostic approaches, as well as prognostic biomarkers.

RESULTS

Cancer Alters the Transcriptome of Human Monocytes

We performed bulk RNA sequencing (RNA-seq) on total monocytes isolated from women with breast (n = 32) or endometrial (n = 3) cancer and from healthy controls (n = 45) and (Figures S1A and S1B). Although there are outliers, principal-component analysis (PCA) and hierarchical clustering segregated the transcriptomic profiles of normal monocytes (Mo) from breast or endometrial cancer patient monocytes (Figures 1A and 1B). Thus, we designated cancer monocytes as tumor-educated monocytes (TEMo). Limma differential expression analysis (DEA) revealed 865 differentially expressed genes (DEGs) in breast TEMo compared with Mo (543 upregulated and 322 downregulated; false discovery rate [FDR] $\leq$ 0.05, Table S1) and 997 DEGs in endometrial TEMo compared with Mo (498 upregulated and 499 downregulated; FDR $\leq$ 0.05, Table S1). Because of the limited size of endometrial TEMo samples, we focused our downstream analysis on the breast TEMo. Gene ontology (GO) analysis reported a number of enriched terms, such as cell migration, angiogenesis, cell communication, and apoptotic process (Figure 1C). A number of genes encoding transmembrane receptors, soluble factors, transcription factors, and enzymes were deregulated, including increased expression of transcripts encoding immune regulatory receptors (*CD200R1*), pro-apoptotic molecules (*TNFSF10*), and pro-angiogenic factors (*HGF* and *ANGPT1*) (Figure 1D). qRT-PCR of monocytic RNA derived from an independent breast cancer cohort confirmed significant increased expression of these genes (Figure 1E).

To understand if this shift in TEMo transcriptomes was driven by a specific subpopulation, we analyzed classical and non-classical monocytes from two independent cancer cohorts as well as healthy women (Figures 1F and S1C–S1E; Table S1). Non-classical monocytes from cancer patients exhibited a significant expansion compared with healthy controls in both cohorts without significant differences between endometrial and breast cancer patients (Figure 1F). This expansion was associated with a significant increase in CX3CL1 and reduction of CCL2 in cancer patients' sera (Figure 1G). The expression level of the main receptor of CCL2, CCR2, did not change among subpopulations and conditions, although CX3CR1, the CX3CL1 receptor, was significantly downregulated in classical monocytes from cancer patients compared with controls, consistent with the alterations in monocytic populations (Figure 1H). We isolated non-classical monocytes from 13 cancer patients (n = 6 breast and n = 7 endometrial) and 5 healthy women, and performed RNA-seq. PCA and hierarchical clustering revealed distinct non-classical monocyte clusters in cancer patients versus healthy volunteers (Figure S1F). Limma DEA revealed 139 DEGs in non-classical monocytes from breast cancer patients compared with healthy individuals (103 upregulated and 36 downregulated; FDR $\leq$ 0.05, Table S1). Similarly, we identified 576 DEGs in non-classical monocytes in endometrial cancer patients compared with healthy individuals (501 upregulated and 75 downregulated; FDR $\leq$ 0.05, Table S1). Hierarchical clustering showed similar patterns of gene expression changes in non-classical monocytes from women with breast and endometrial cancer compared with healthy women (Figure S1G).

Given the significant transcriptional differences in monocytes between cancer patients and healthy volunteers, we hypothesized that a TEMo signature from a liquid biopsy with minimal processing could be generated for breast cancer detection. We tested this hypothesis using total monocytes and a Recursive Feature Elimination with a Random Forest algorithm. The dataset was split into training (70%, n = 55, 32 healthy individuals, 23 cancer patients) and testing sets (30%, n = 22, 13 healthy individuals, 9 cancer patients). In the training set, the algorithm selected 17 highest performing genes that yielded an average of 85% accuracy, 88% sensitivity, and 83% specificity during cross-validation (Figure S1H; Table S1). Subsequent validation using the test set yielded 82% accuracy, 100% sensitivity, and 69% specificity (Figures 1I and 1J) and area under curve of 96% to detect cancer (Figure 1K). In contrast, random classifiers, as determined by 1,000 rounds of random class permutations during model training, had no predictive power (mean accuracy: 53%, SD ± 6.8%, p = 0.001) (Figure S1I).

(I and J) Confusion matrix (I) and summary of results of Recursive Feature Elimination with Random Forest (RFE-RF) classification on the testing set (n = 22) for breast TEMo (J).
(K) Receiver operating characteristic curves of RFE-RF classification in the training and test set.
(E and H) Data depicted as means ± SEM; (F and G) horizontal bars represent the mean of the individual values ± SD; (E–H) Student's t test; *p < 0.01, **p < 0.001, ***p < 0.0001.
See also Figure S1 and Table S1.

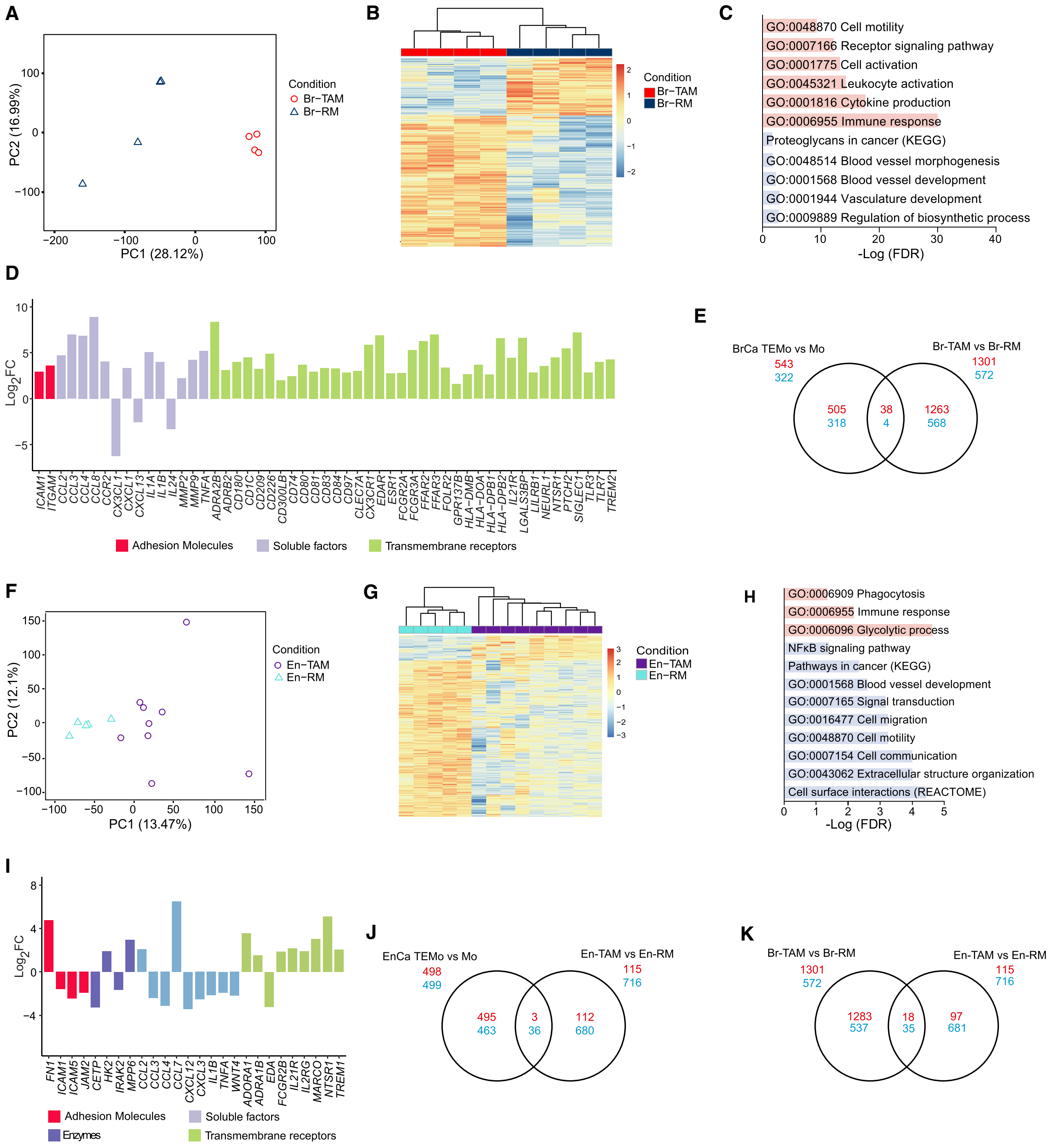

Figure 2. TAMs from Breast and Endometrial Cancers Exhibit Cancer-Specific Transcriptional Profiles

(A) PCA plot of n = 13,668 genes expressed in breast tissue-resident macrophages (Br-RM) (n = 4) and breast cancer TAMs (Br-TAM) (n = 4).

(B) Hierarchical clustering of all DEGs between Br-RM and Br-TAM. Expression values are *Z* score transformed and samples clustered using complete linkage and Euclidean distance.

(C) GO analysis of DEGs between Br-TAM and Br-RM (blue, downregulated genes; red, upregulated genes).

(D) Bar plot of selected DEGs in Br-TAM (FDR $\leq$ 0.05).

(E) Venn diagram of commonly regulated transcripts in Br-TAM and TEMo (red, upregulated; blue, downregulated).

(F) PCA plot of n = 13,739 genes expressed in endometrial tissue-resident macrophages (En-RM) (n = 5) from healthy individuals and endometrial cancer TAMs (En-TAM) (n = 9).

(G) Hierarchical clustering of all DEGs between En-RM and En-TAM. Expression values are Z score-transformed and samples clustered using complete linkage and Euclidean distance.

(legend continued on next page)

Gene Expression Profiles of TAMs in Human Breast and Endometrial Cancers

There is significant evidence showing pro-tumoral profiles of TAMs in mouse models of cancer; however, a detailed characterization of their transcriptomes and phenotypes in human cancers is lacking. Thus, we analyzed TAM transcriptomes by RNA-seq from breast and endometrial cancer in comparison with resident macrophages from homeostatic tissue after fluorescence-activated cell sorting (Figure S2A). PCA and hierarchical clustering revealed distinct clusters of breast tissue-resident macrophages (Br-RM) and breast cancer TAMs (Br-TAM) (Figures 2A and 2B). Limma DEA revealed 1,873 DEGs in Br-TAM compared with Br-RM (1,301 upregulated and 572 downregulated; FDR $\leq$ 0.05, Table S2). GO analysis reported enriched GO terms, such as cell motility and activation, vasculature development, and immune response (Figure 2C). Br-TAM showed increased transcript abundance of genes encoding transmembrane receptors associated with immune cell activation and antigen presentation, such as major histocompatibility complex class II molecules, Fc receptors, T cell co-stimulatory molecules, Toll-like receptors, and immunoglobulin receptor superfamilies (Figure 2D). Although in mice CD163 is often referred to as a TAM marker, we did not observe a significant difference in CD163 expression between Br-RM and Br-TAM (Figure S2B). Comparison of DEGs between breast TEMo and Br-TAM showed minimal overlap (Figure 2E).

PCA and hierarchical clustering revealed distinct clusters of endometrial tissue-resident macrophages (En-RM) and endometrial cancer TAMs (En-TAM) (Figures 2F and 2G). Limma DEA between En-RM and En-TAM identified 831 DEGs (115 upregulated and 716 downregulated; FDR $\leq$ 0.05, Table S2). GO analysis reported enriched GO terms, such as phagocytosis, immune response, cell communication, and blood vessel development (Figure 2H). In addition, a number of genes encoding transmembrane receptors, soluble factors, and enzymes were differentially expressed; the scavenger receptors *MARCO*, *TREM1*, *FCG2RB*, and *IL21RG* were upregulated in En-TAM compared with En-RM (Figure 2I). Similar to that found in breast cancer, En-TAMs have minimal similarity to endometrial TEMo (Figure 2J).

To better understand TAMs in different cancer types, we compared the gene expression profiles of Br-TAM and En-TAM. PCA and hierarchical clustering revealed two distinct groups (Figure S2C) with very few DEGs commonly deregulated (18 upregulated and 35 downregulated, Figure 2K; Table S2), indicating that breast and endometrial cancers activate cancer tissue-specific transcriptional profiles in TAMs. Resident macrophages from endometrial and breast tissue also exhibited a distinct transcriptional profile confirming the diversity of tissue macrophage phenotypes in homeostatic states (Figure S2D).

Macrophages exhibit distinct phenotypes and have been classified into two alternative polarization states, referred to as "M1" and "M2," with the latter being immune suppressive and pro-tumoral (Martinez et al., 2006). To determine whether these polarization states exist within human En- and Br-TAM, we performed gene set enrichment analysis using the M1/M2 signature as proposed by Martinez et al. (Table S2). Neither Br- nor En-TAM showed a preferential enrichment for M2-associated genes, supporting the idea that TAM phenotypes are much more complex and cannot be categorized into binary states (Figures S2E and S2F). Similarly, canonical markers for M2 that have been identified in mice, such as *Arg1* (arginase-1), were minimally, and not differentially, expressed in either Br- or En-TAM (Table S2).

TAM Gene Signature Is Enriched in Aggressive Breast Cancer Tumors

Increased density of TAMs has been associated with poor clinical outcomes in many human cancers (Yang et al., 2018). Importantly, studies using transcriptomic datasets have identified immune cell-specific gene sets to deconvolute the tumor microenvironment and its role in cancer progression (Charoentong et al., 2017; Gentles et al., 2015). Taking advantage of a previously defined and validated compendium of immune cells (Bindea et al., 2013; Tamborero et al., 2018), we sought to identify a TAM-specific immune signature. We focused on Br-TAM, as breast cancer has a greater number of in-depth studies published. We selected upregulated genes in Br-TAM compared with Br-RM ($Log_2FC > 3$, FDR $\leq$ 0.05) that were also highly co-expressed in the METABRIC cohort (Curtis et al., 2012), while filtering out genes belonging to other immune cell types (Tamborero et al., 2018), or those expressed by cancer cells (Table S3). As a result, we identified a 37-gene TAM signature (Table S3). We then performed whole-tumor RNA-seq on an independent cohort of 47 breast cancer patients (cohort 3, Table S3) and evaluated the expression of our TAM signature on this dataset. Colony-stimulating factor 1 (CSF1) is the major macrophage growth factor regulating their survival, differentiation and proliferation. A previous study of breast cancer defined a 112-gene CSF1 response signature associated with higher tumor grade, decreased expression of estrogen receptor (ER) and progesterone receptor (PR), and higher mutation rate (Beck et al., 2009). Using this CSF1 response signature, we stratified our dataset into CSF1-high, CSF1-mid, and CSF1-low groups then evaluated the TAM signature expression (TAM signature score). Results indicated that the CSF1-high group had a significantly higher TAM signature score compared with CSF1-mid and CSF1-low groups, suggesting that TAMs are associated with more aggressive tumors (Figure 3A). We then assigned these samples to breast cancer molecular subtypes based on the PAM50 classification (Parker et al., 2009), with the TAM signature showing significantly higher expression in human epidermal growth factor receptor 2 (Her2) compared with luminal A or B samples ($p = 0.02$) (Figure 3B).

We investigated whether the identified TAM signature was associated with clinical outcome in the METABRIC cohort. We observed a higher expression of the TAM signature in basal, claudin-low, Her2, and luminal B compared with luminal A

(H) GO analysis of DEGs between En-TAM and En-RM (blue, downregulated genes; red, upregulated genes).

(I) Bar plot of selected DEGs in En-TAM (FDR $\leq$ 0.05).

(J and K) Venn diagram of commonly regulated transcripts between En-TAM and TEMo (J) and En-TAM and Br-TAM (red, upregulated; blue, downregulated) (K). See also Figure S2, and Table S2.

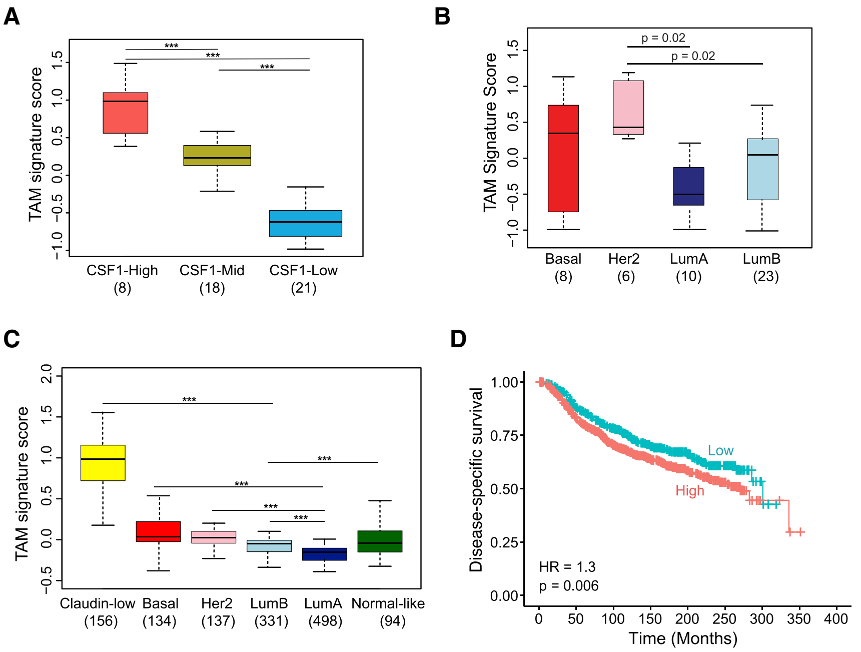

Figure 3. Breast TAM Signature Is Associated with Clinical Outcomes

(A and B) Boxplot showing TAM signature score stratified by the CSF1 signature (A) and across breast cancer subtypes in cohort 3 (n = 47) (B).

(C) TAM signature score across PAM50 molecular subtypes in the METABRIC cohort (n = 1,350).

(D) Disease-specific survival of the METABRIC cohort according to the TAM signature expression.

Boxplots depict the first and third quartiles, with the median shown as a solid line inside the box and whiskers extending to 1.5 interquartile range from first and third quartiles.

(A–C) One-way ANOVA with Tukey's *post hoc* multiple comparisons test (***$p < 0.0001$). (D) The p value is based on the Wald test.

See also Table S3.

tumors, again showing an association of the TAM signature with more aggressive tumors (Figure 3C). Consistent with these data, high expression of the TAM signature was significantly associated with shorter disease-specific survival (DSS) (Figure 3D). A previously reported macrophage immune signature (Bindea et al., 2013; Tamborero et al., 2018), consisting mainly of lineage markers, showed a similar trend of high expression in aggressive tumors, but was not significantly associated with DSS (hazard ratio [HR] = 1.17, p = 0.1, Table S3). Taken together, these results suggest a positive association of unique populations of TAMs with poor clinical outcomes and more aggressive breast cancers.

Identification of Breast TAM Markers

One of the main limitations of targeting TAMs for therapeutic approaches is the lack of reliable and specific markers. To address this, we selected genes encoding transmembrane receptors in our TAM signature. We selected *SIGLEC1*, which encodes CD169, as it was one of the top upregulated genes in Br-TAM compared with Br-RM (Log_2FC = 7.2, FDR = 0.0017) and it was also correlated with expression of the pan-macrophage marker *CD163* (Figure 4A). In the METABRIC cohort, univariate analysis showed that *SIGLEC1* high expression was significantly associated with shorter DSS (Figure 4B; Table S4). Consistent with this, in Cox multivariate analysis after adjusting for clinical parameters such as ER, PR, Her2, grade, and tumor size, *SIGLEC1* high expression was independently significantly associated with shorter DSS (HR = 1.42, $p = 1.85 \times 10^{-0.4}$, Table S4). Validation by qPCR confirmed the significant upregulation of *SIGLEC1* mRNA observed in the RNA-seq analysis (Figure 4C). Furthermore, *SIGLEC1* showed significantly higher expression in breast tumor stroma compared with normal breast stroma (Figure 4D).

We used multicolor flow cytometric analysis to determine SIGLEC1 expression at the protein level in an independent cohort of breast cancer patients and found that SIGLEC1 was expressed on Br-TAM, but not on other immune cells or $CD45^-$ non-immune cells, indicating specificity to macrophages/TAMs (Figures S3A and S3B). In the circulation, classical and non-classical monocytes (Figures S3C and S3D), but not granulocytes (Figures S3E and S3F), exhibited low expression of SIGLEC1, with no difference between cancer and non-cancer patients. Having established that SIGLEC1 is significantly expressed only by Br-TAM, we performed immunofluorescent staining using anti-SIGLEC1 and anti-CD163 antibodies on tissue biopsies from patients with invasive breast cancer and benign lesions (Figure 4E). Using machine-learning image analysis for unbiased quantification, we were able to segment and classify CD163 and SIGLEC1 single- and double-positive populations and determine their numbers within whole and subregions of the tissue sections. Cancer tissues had higher numbers of macrophages per mm^2 tissue area, and a higher percentage of $SIGLEC1^+$ cells compared with benign tissue (Figure 4F); results that were further confirmed by confocal microscopy of the stained sections (Figure S3G). These results indicate that SIGLEC1 is a human breast TAM-associated marker.

$SIGLEC1^+$ Macrophages Accumulate in Basal and Her2 Breast Cancers

To investigate expression of SIGLEC1 in different breast cancer subtypes we performed multiplex immunohistochemistry (Tsujikawa et al., 2017) on breast cancer tissues that had been independently acquired from cohort 3. Using image cytometry we identified three distinct Br-TAM subtypes ($CSFR1^+CCR2^-CD68^+CD163^+SIGLEC1^-$, $CSFR1^+CCR2^-CD68^+CD163^+SIGLEC1^+$, and $CSFR1^+CCR2^-CD68^+CD163^-SIGLEC1^+$, Figure S4A) confirming results reported in Figure 4F. Quantification of these three Br-TAM populations revealed enrichment in basal tumors compared with Her2 and luminal subtypes, while the three subsets were almost absent in tissues from prophylactic mastectomies (Figures S4B and S4C). This is consistent with the increased expression of the TAM signature in aggressive breast tumors at the mRNA level.

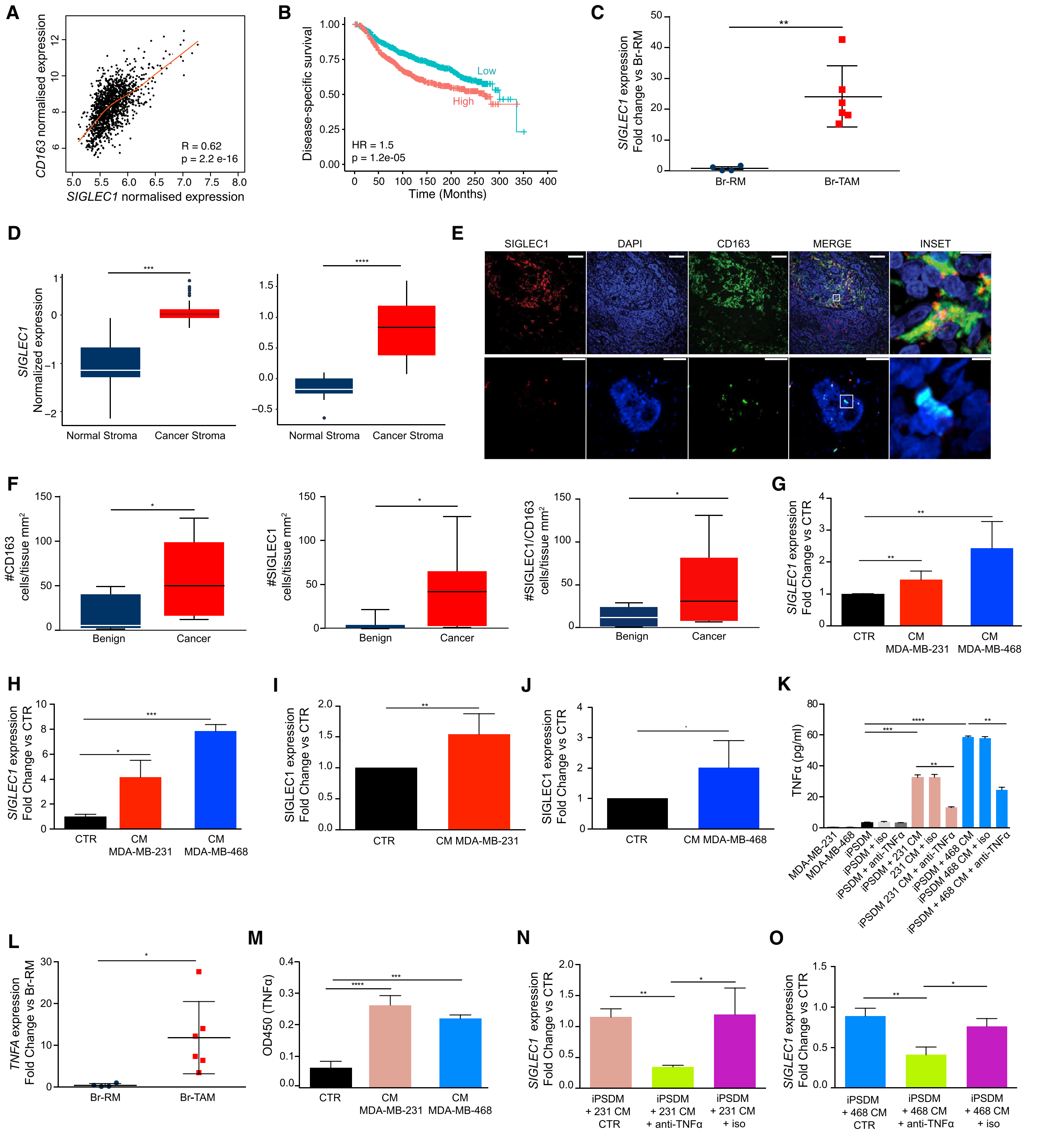

Figure 4. Breast TAM Transcriptomes Are Associated with Clinical Outcomes and Reveal TAM-Specific Markers

(A) Scatterplot showing Pearson's correlation between *CD163* and *SIGLEC1* expression in the METABRIC cohort. Red line indicates local regression (LOESS) fit.

(B) Disease-specific survival according to the mRNA level of *SIGLEC1* in the METABRIC cohort.

(C) Expression of *SIGLEC1* mRNA in Br-RM (n = 4) and Br-TAM (n = 6).

(D and E) *SIGLEC1* expression in the Finak et al. (2008) dataset (left) and the Karnoub et al. (2007) dataset (right). Expression calculated from the median centered normalized values. The p values were estimated using a Wilcoxon rank-sum test. Boxplots depict the first and third quartiles, with the median shown as a solid line inside the box and whiskers extending to 1.5 interquartile range from first and third quartiles (D). Data points beyond the limit of lines represent outliers (black dots). CD163 and SIGLEC1 immunofluorescent (IF) staining (n = 5) (E). Stains from cancer (top) and benign sample (bottom) are shown representative of n = 12 independent tumors analyzed. Single channels and merge are shown. Inset representing a double-positive SIGLEC1 and CD163 macrophage (top) and a single-positive CD163 macrophage (bottom). Scale bars, 50 μm, and 5 μm (inset).

(legend continued on next page)

Next, we investigated the regulation of SIGLEC1 expression in human macrophages using human monocyte-derived macrophages (MDMs), induced pluripotent stem cell (iPSC)-derived macrophages (iPSDM), and THP1 cells differentiated into macrophages using phorbol-12-myristate-13-acetate (PMA-THP1). All three were exposed to conditioned medium (CM) from triple-negative breast cancer cell lines MDA-MB-231 and MDA-MB-468 (Neve et al., 2006). CM from both cell lines increased expression of *SIGLEC1* mRNA in MDM and PMA-THP1 (Figures 4G and 4H). In addition, CM enhanced SIGLEC1 protein expression on the cell surface of iPSDM (Figures 4I and 4J).

To further investigate the stimulus generated by cancer cells, we stimulated PMA-THP1 with a panel of pro- and anti-inflammatory cytokines and measured *SIGLEC1* mRNA expression by qPCR. The inflammatory mediator positive control, lipopolysaccharides, and the pro-inflammatory cytokine, tumor necrosis factor alpha (TNF-α), were the main modulators of *SIGLEC1* expression, while interleukin 1β (IL-1β) and interferon γ produced a modest effect (Figure S4D). Conversely, anti-inflammatory cytokines did not affect *SIGLEC1* expression in a significant way, except for a downregulation after combined exposure with IL-4 and transforming growth factor β (Figure S4D). We tested if cancer cells produce TNF-α, by ELISA of MDA-MB-231 and MDA-MB-468 CM, but did not detect significant levels (Figure 4K). In contrast, qPCR analysis indicated a significant upregulation of *TNFA* mRNA in Br-TAM compared with Br-RM (Figure 4L). Consistent with this elevated expression in Br-TAM, MDM, and iPSDM, incubated with either MDA-MB-231 or MDA-MB-468 CM, produced significantly higher levels of TNF-α compared with untreated controls at the protein level (Figures 4K and 4M; Table S4). We next neutralized TNF-α in MDA-MB-231 and MDA-MB-468 CM-treated iPSDM (Figure 4K), and exposed new iPSDM to the neutralized CM. TNF-α neutralization resulted in a significant reduction of *SIGLEC1* expression compared with isotype control-treated CM (Figures 4N and 4O). These results indicate that Br-TAM responds to cancer signals by upregulating the expression of SIGLEC1 and by producing TNF-α, which further supports SIGLEC1 expression in macrophages.

CCL8 Is a Breast TAM Marker

To identify additional mediators of the crosstalk between human cancer cells and macrophages, we performed inflammatory gene expression qPCR array and found 19 commonly upregulated pro-inflammatory genes in PMA-THP1 cells incubated with MDA-MB-231 and MDA-MB-468 CM (Figures 5A–5C; Table S5). Of those, seven were also upregulated in Br-TAM compared with Br-RM (Figure 5D), among which *CCL8* was the most significantly upregulated. Interestingly, CCL8 has been reported to play a role in the tumor microenvironment by supporting mouse mammary cancer cell dissemination (Farmaki et al., 2016). In our data, *CCL8* was correlated with *CD163* expression (Figure 5E). In the METABRIC cohort, univariate analysis showed that *CCL8* high expression was significantly associated with shorter DSS (Figure 5F; Table S5). However, in Cox multivariate analysis, after adjusting for clinical parameters such as ER, PR, Her2, grade, and tumor size, high CCL8 expression was not independently significantly associated with shorter DSS (HR = 1.16, p = 0.13, Table S5). Internal validation by qPCR on samples used for RNA-seq showed significant upregulation of the *CCL8* transcript in Br-TAM (Figure 5G). We next validated these data by incubating PMA-THP1, MDM, and iPSDM with cancer CM and showed elevated CCL8 mRNA and protein levels (Figures 5H and S5A–S5C). In addition, fluorescence *in situ* hybridization analysis of breast cancer tissue sections revealed that *CCL8* mRNA is found in Br-TAM but not in cancer cells (Figure 5I). There were no differences in CCL8 serum levels between healthy individuals and cancer patients, indicating local production (Figure S5D). CCL8 production in human macrophages was induced by both pro- and anti-inflammatory stimulation (Figures S5E and S5F) consistent with reports using cultured mouse macrophages (Makita et al., 2015).

Similarly to the observations with SIGLEC1, TNF-α modulated the expression of *CCL8* (Figure S5E). We neutralized TNF-α in MDA-MB-231 and MDA-MB-468 CM-treated iPSDM with neutralizing antibodies and exposed new iPSDM to the neutralized CM. TNF-α neutralization resulted in a significant reduced *CCL8* expression compared with isotype control-treated CM, confirming a role for TNF-α in CCL8 regulation in macrophages exposed to cancer cell CM (Figures 5J and 5K). CCL8 treatment of both cancer cell lines significantly upregulated the expression of CSF1 mRNA and protein ($Log_2FC > 1$, $p < 0.05$, Figure 5L), as well as TNF-α and IL-1β (Figure 5M).

(F) Quantification of $CD163^+$ (left), $SIGLEC1^+$ (center), and $CD163^+$ and $SIGLEC1^+$ (right) cells per mm^2 of tissue in benign (n = 4) and breast cancer samples (n = 8). Boxplots depict the first and third quartiles, with the median shown as a solid line inside the box and whiskers extending to 1.5 interquartile range from first and third quartiles.

(G and H) *SIGLEC1* expression in primary MDM- (G) and PMA-treated THP1 cells (H) stimulated for 24 h with culture medium (CTR) normalized as 1, MDA-MB-231 conditioned medium (CM) or MDA-MB-468 CM. Data are depicted as fold change versus CTR (n = 3).

(I and J) Flow cytometric analysis of SIGLEC1 expression in iPSDM cells without stimulation (CTR) or stimulated with MDA-MB-231 (I) or MDA-MB-468 (J) CM (n = 3).

(K) TNF-α levels in supernatants of iPSDM incubated for 24 h with CTR plus isotype control or CTR plus anti-TNF-α antibody. Same conditions are shown for MDA-MB-231 and MDA-MB-468 CM (n = 3). Results are expressed as pg/mL.

(L) Expression of *TNFA* mRNA in Br-RM (n = 4) and Br-TAM (n = 6).

(M) TNF-α protein levels in supernatants of MDM incubated for 24 h with MDA-MB-231 and MDA-MB-468 CM or CTR. Results are expressed as optical density at 450 nm (OD_{450}) (n = 3).

(N and O) *SIGLEC1* mRNA expression in iPSDM stimulated for 24 h with MDA-MB-231 CM normalized as 1 (CTR), MDA-MB-231 CM + TNF-α neutralizing antibody and MDA-MB-231 CM + isotype control antibody (N) or with MDA-MB-468 CM normalized as 1 (CTR), MDA-MB-468 CM + TNF-α neutralizing antibody and MDA-MB-468 CM + isotype control antibody (O) (n = 3 each).

(C and L) Horizontal bars represent the mean of the individual values ± SD; (G–K and M–O) data depicted as means ± SEM; (B) The p value is based on the Wald test; (C, D, I, J, and L) Student's t test; (F) two-way ANOVA; (H, K, and M–O) one-way ANOVA; *p < 0.01, **p < 0.001, ***p < 0.0001, ****p < 0.00001.

See also Figures S3 and S4 and Table S4.

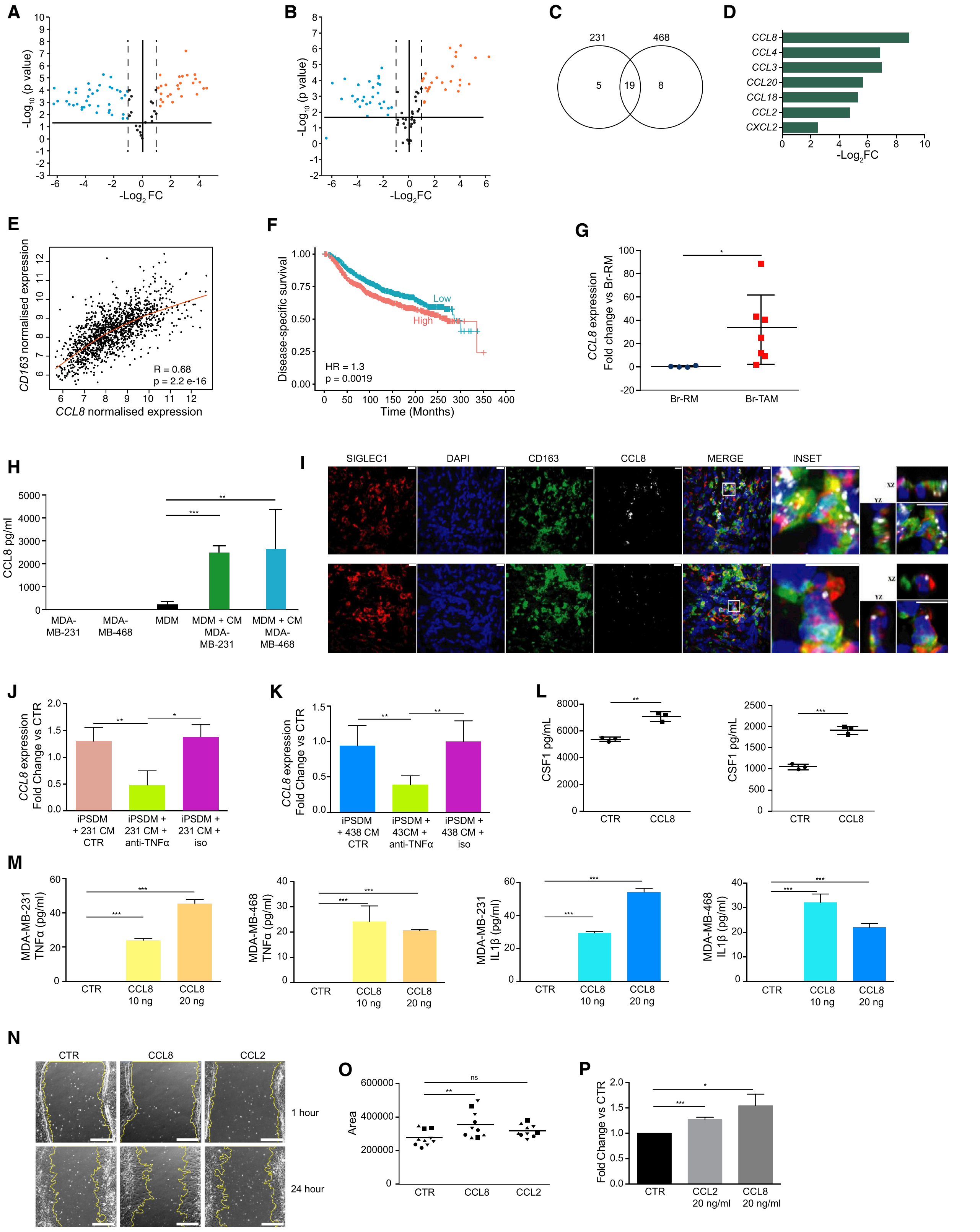

Figure 5. TAMs and Cancer Cells Engage in Cytokine Feedback Loops to Support CCL8 and SIGLEC1 Expression in Breast Cancer TAMs

(A and B) Volcano plot showing genes whose expression was significantly (Log$_2$FC ± 1, $p < 0.05$) deregulated in PMA-THP1 cells after incubation with MDA-MB-231 (A) or MDA-MB-468 (B) CM for 24 h (n = 3 each).

(legend continued on next page)

CCL8 Enhances Breast Cancer Cell Motility and Monocyte Recruitment

We investigated the effect of CCL8 on cancer cells. Cancer cell lines were analyzed for expression of the five reported CCL8 receptors (Figures S5G and S5H). Of these CCR1, 2, 5, and 8 were detected on the cell surface of both MDA-MB-231 and MDA-MB-468 cells. CCL8 receptors, mainly CCR1 and CCR2, have also been shown to be expressed on tumor cells in human breast cancers (Fang et al., 2012; Shin et al., 2017). Stimulation with recombinant CCL8 (rCCL8) did not affect cell proliferation of either breast cancer cell line (Figure S5I). We stimulated MDA-MB-231 and MDA-MB-468 with rCCL8 and performed a qPCR array for genes associated with breast cancer progression. Using stringent criteria for changes in gene expression ($Log_2FC > 2$, $p < 0.05$) (Figures S6A and S6B), six genes were identified that were commonly upregulated in both cell lines following stimulation with rCCL8 (Figure S6C). The product of these genes have been predicted to be involved in cancer cell invasion (*MMP2*, *MMP9*, *ADAM23*) (Roomi et al., 2009) and progression (*IL6*, *EGF*, and *GLI1*) (Knüpfer and Preiß, 2006; Makita et al., 2015) (Figure S6D; Table S5). Similar genes were identified by a metastasis qPCR array after exposure of MDA-MB-231 and MDA-MB-468 with CM from cancer cell-primed MDM (Figures S6E–S6H; Table S5). Consistent with the upregulated expression of genes involved in invasion, rCCL8 treatment enhanced motility of MDA-MB-231 cells (Figures 5N and 5O) to a greater extent than previously reported for CCL2 (Fang et al., 2012). Finally, as TEMo express CCR2 as the only CCL8 receptor differentially expressed (Table S1), we assessed the ability of CCL8 to recruit monocytes using an *in vitro* chemotaxis assay with THP1 monocytic cells in the presence of CCL2 and CCL8 as chemo-attractants. Both CCL2 and CCL8 attract these monocytic cells compared with controls (Figure 5P).

SIGLEC1/CCL8 Gene Signature Is an Independent Prognostic Factor in ER⁺ Breast Cancer

To assess whether a *SIGLEC1/CCL8* two-gene signature had clinical relevance in breast cancer, Cox proportional hazard regression analysis was performed on a breast cancer stroma dataset (Finak et al., 2008) representing 53 patients suffering 17 recurrence events reported over a median follow-up time of 8.7 years. Gene expression values of *SIGLEC1/CCL8* were dichotomized into high- and low-expression groups according to all possible cutoffs (Pearce et al., 2017). Univariate analysis revealed that *SIGLEC1/CCL8* high expression was associated with shorter recurrence-free survival (Figure 6A; Table S6). To further validate the clinical relevance of the *SIGLEC1/CCL8* gene signature, we utilized the METABRIC cohort with 456 breast cancer-specific events over a median follow-up time of 9.69 years. In univariate analysis high expression of *SIGLEC1/CCL8* was significantly associated with shorter DSS (Figure 6B; Table S6), along with Her2 status (HR = 2.1, $p = 2.3 \times 10^{10}$), grade (HR = 1.8, $p = 1.9 \times 10^{-9}$) and tumor size (HR = 1.8, p = 0.002). Conversely, ER (HR = 0.6, $p = 7.8 \times 10^{-7}$) and PR (HR = 0.64, $p = 3 \times 10^{-6}$) status were significantly associated with better DSS. In Cox multivariate analysis, *SIGLEC1/CCL8* high expression was associated with shorter DSS but did not reach significance (HR = 1.2, p = 0.06, Table S6).

In a subset of ER^+Her2^- patients from the METABRIC cohort, univariate analysis revealed that *SIGLEC1/CCL8* high expression was significantly associated with shorter DSS (Figure 6C), along with grade (HR = 1.7, $p = 9 \times 10^{-6}$) and age (HR = 1.5, $p = 2.2 \times 10^{-3}$) (Table S6). Cox multivariate analysis demonstrated that *SIGLEC1/CCL8* high expression was independently significantly associated with shorter DSS (HR = 1.35, p = 0.014) along with grade (HR = 1.54, $p = 3.4 \times 10^{-4}$) and age (HR = 1.44, p = 0.008).

DISCUSSION

In mouse models of cancer, monocytes are recruited to primary or metastatic tumors where they differentiate to TAMs, which promote tumor progression and metastasis (Arwert et al., 2018). Here, we show that circulating monocytes respond to breast and endometrial cancers with an expansion in the non-classical

(C) Venn diagram of commonly upregulated transcripts between MDA-MB-231-treated (left circle) and MDA-MB-468-treated (right circle) THP1 cells.
(D) Selection of pro-inflammatory genes commonly upregulated in Br-TAM (n = 4) (from RNA-seq analysis) and PMA-THP1 (n = 3) (qPCR).
(E) Scatterplot showing Pearson's correlation between *CD163* and *CCL8* expression in the METABRIC cohort. Red line indicates local regression (LOESS) fit.
(F) Disease-specific survival according to the mRNA level of *CCL8* in the METABRIC cohort.
(G) *CCL8* mRNA expression in Br-RM (n = 4) and Br-TAM (n = 7). Data are expressed as fold change versus Br-RM.
(H) CCL8 levels in CM from MDA-MB-231, MDA-MB-468, MDM, and MDM incubated for 24 h with the two cancer cell CM, respectively (n = 3).
(I) IF and fluorescence *in situ* hybridization for *CCL8* mRNA (top) or a DapB-control RNA (bottom) in breast cancer samples. Scale bars, 10 μm (n = 3). Inset representing a $SIGLEC1^+CD163^+$ macrophage-expressing *CCL8* mRNA (top) or DapB-control mRNA (bottom). XY, XZ, and YZ projections are shown (right panels).
(J and K) *CCL8* mRNA expression in iPSDM stimulated for 24 h with MDA-MB-231 CM normalized as 1 (CTR), MDA-MB-231 CM + TNF-α neutralizing antibody and MDA-MB-231 CM + isotype control antibody (J), or with MDA-MB-468 CM normalized as 1 (CTR), MDA-MB-468 CM + TNF-α neutralizing and MDA-MB-468 CM + isotype control antibody (K) (n = 3 each).
(L and M) CSF1 levels (L) and TNF-α and IL-1β levels (M) in supernatants from unstimulated MDA-MB-231 or MDA-MB-468 (CTR), and MDA-MB-231 or MDA-MB-468 incubated for 24 h with 10 or 20 ng/mL (or 20 ng/mL for CSF1) of rCCL8 (n = 3 each).
(N) *In vitro* scratch assay of untreated MDA-MB-231 or treated with CCL8 or CCL2 for the indicated period of time, yellow line = cell culture margins (n = 4). Scale bars, 500 μm.
(O) Quantification of *in vitro* scratch assay covered by MDA-MB-231 after 24 h (calculated as area covered at 24–1 h) in untreated (CTR), and CCL8- and CCL2-treated cells. Same symbols represent mean of technical replicates (n = 4).
(P) THP1 chemotaxis assay for CCL2 and CCL8. Cells were incubated with medium alone (CTR) or with 20 ng/mL of rCCL2 or rCCL8. Results shown as fold change versus CTR at 72 h (n = 3).
(H, J, K, M, and P) Data depicted as mean ± SEM; (G and L) horizontal bars represent the mean of the individual values ± SD; (O) horizontal bars represent the mean of the individual values; (F) the p value is based on the Wald test; (G and L) Student's t test; (H, J, K, M, and P) one-way ANOVA; (O) two-way ANOVA; *p < 0.01, **p < 0.001, ***p < 0.0001.
See also Figures S5 and S6, Table S5.

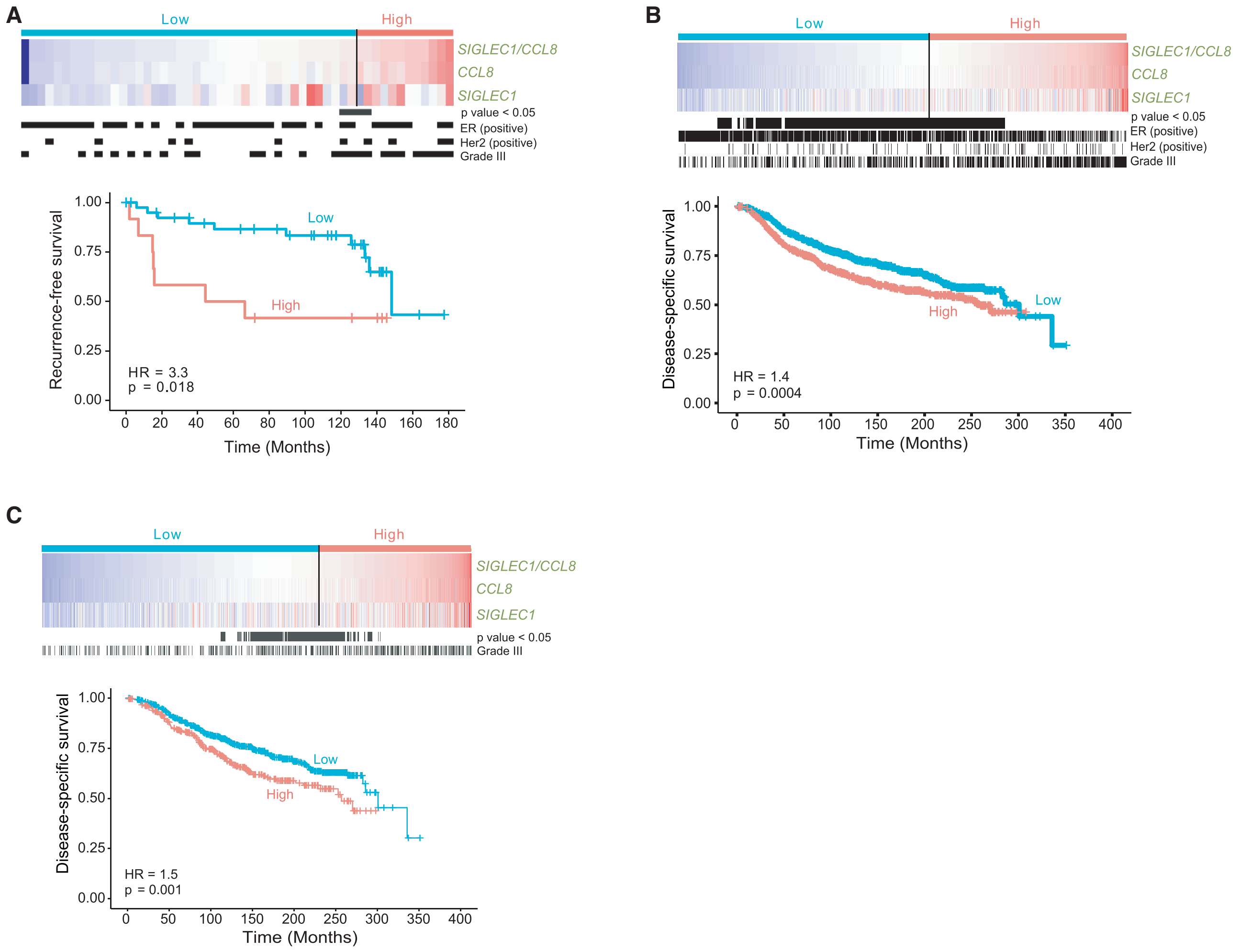

Figure 6. High Expression of SIGLEC1/CCL8 Is Associated with Poor Outcome in Breast Cancer Patients

(A) Heatmap and recurrence-free survival according to mRNA levels of *SIGLEC1* and *CCL8* in the breast cancer stroma dataset (Finak et al., 2008).

(B and C) Heatmap and disease-specific survival in all (B) and $ER^{+}Her2^{-}$ (C) patients from the METABRIC cohort. All significant cutoff points ($p < 0.05$) are shown in black. Black vertical lines indicate positivity for ER and Her2 expression or grade III tumors. All p values are based on the Wald test.

population and alteration of transcriptomes in both monocytic populations compared with healthy women. Using total monocyte transcriptional profiles we identified a 17-gene signature that indicated the presence of cancer. Alterations in non-classical populations have also been shown to be negatively associated with breast tumor size and disease stage (Feng et al., 2011). Monocytes in renal carcinoma and colorectal cancer patients also showed distinct transcriptional alterations compared with healthy individuals (Chittezhath et al., 2014). In mouse models, inhibition of classical monocyte recruitment inhibited metastasis (Qian et al., 2011), while depletion of non-classical monocytes correlated with enhanced metastasis through inhibition of natural killer cell activity (Hanna et al., 2015). However, non-classical monocytes have been shown to contribute to anti-vascular endothelial growth factor therapy resistance in mouse models of cancer (Jung et al., 2017). This therapy is associated with enhanced CX3CL1 levels in human colon cancers, leading to the recruitment of non-classical monocytes to the vascular bed of the tumor, where they promote accumulation of neutrophils and immune suppression through IL-10 secretion (Jung et al., 2017). We detected significantly higher levels of CX3CL1 in the sera of breast cancer patients compared with healthy controls. This chemokine increase could explain the elevated number and activation of monocytes.

Despite the strong evidence for pro-tumoral roles of TAMs in mouse models of cancer (Cassetta and Pollard, 2018), little is known about them in humans. Thus, we profiled TAMs in breast and endometrial cancers. Surprisingly, in contrast to monocytes, TAM transcriptomes from endometrial and breast cancers are distinct from each other, from their respective resident macrophages, and from their progenitor monocytes. These data suggest the existence of cancer-specific niches that influence the TAM transcriptional profiles according to tumor location and subtype. High expression of macrophage gene signatures has been associated with high tumor grade and poor clinical outcomes (Gentles et al., 2015). In our study, we identified a 37-gene TAM signature that is highly expressed in the most aggressive breast cancer subtypes and enriched in a CSF1-high

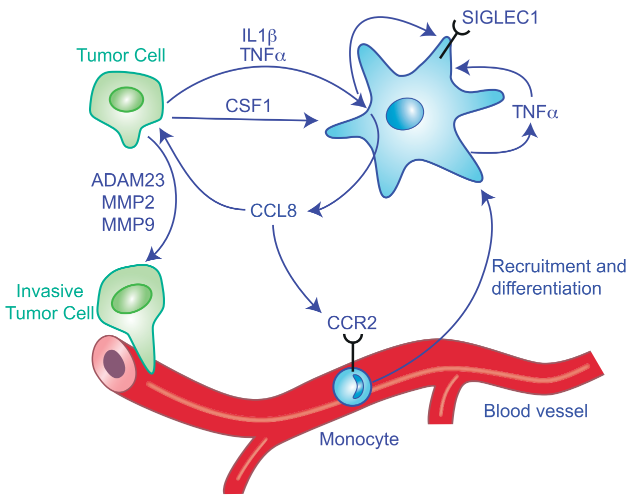

Figure 7. Schematic Representation of the Crosstalk between Br-TAM and Cancer Cells

Tumor cells upregulate SIGLEC1, TNF-α, and CCL8 expression in Br-TAM. In turn, cancer cells respond to CCL8 stimulation by producing CSF1, IL-1β, and TNF-α, which further contribute to the positive feedback loop.

group that has been previously associated with higher tumor grade, decreased expression of ER and PR, and higher mutation rate (Beck et al., 2009). The TAM signature was also associated with shorter DSS in the METABRIC cohort. These results, along with recent evidence of the role of TAMs in chemo- and immune-therapy resistance (Neubert et al., 2018) highlight the need to study TAMs in human cancers and to identify markers for TAM-specific targeting. Therefore, we focused on transmembrane receptors included in the TAM signature, of which, SIGLEC1, a sialic binding receptor mainly expressed by macrophages, was the most highly differentially expressed in Br-TAM compared with Br-RM. In homeostatic conditions, SIGLEC1$^+$ macrophages are mainly in the bone marrow, liver, spleen, colon, and lymph node, and they are involved in erythropoiesis and adaptive immune responses (Chávez-Galán et al., 2015). Consistent with our findings, SIGLEC1$^+$ macrophages have been identified in colorectal (Li et al., 2015) and hepatocellular carcinoma (Zhang et al., 2016). Infiltration of SIGLEC1$^+$ macrophages in colorectal cancer was associated with tumor progression, but in hepatocellular carcinoma they predicted favorable patient outcomes (Zhang et al., 2016), underpinning the hypothesis that TAM phenotypes/activation are organ and cancer specific.

To elucidate the crosstalk between human TAMs and breast cancer cells, we focused on soluble factors produced by TAMs in response to cancer cell CM. Our screening identified CCL8 as the top upregulated soluble factor in Br-TAM. In mouse models, CCL8 has a role in metastasis formation in melanoma (Barbai et al., 2015) and promoted tumor cell invasion and motility in mammary cancer models (Farmaki et al., 2016). SIGLEC1$^+$ macrophages in the mouse intestine produce high levels of CCL8 in response to inflammatory stimuli (Asano et al., 2015). CCL8 production also sustains colitis induced by dextran sulfate sodium treatment and to recruit pro-inflammatory monocytes to the inflamed site. We demonstrated that TAMs are the major source of CCL8, and CCL8 and SIGLEC1 engage in a tumor cell-TAM regulatory loop, involving TNF-α, which in turn enhances their expression and leads to increased tumor cell motility. Our data showed that cancer cells and TAMs secrete high levels of TNF-α that further supports CCL8 production in the tumor microenvironment, and that cancer cells respond to the presence of CCL8 by producing significant higher levels of the major survival and proliferation factor for macrophages CSF1, which further propagate the auto-stimulatory loop (Figure 7). The high concentration of CCL8 not only supports the cancer-TAM crosstalk but also acts as a monocyte chemoattractant. Interestingly, in mouse models of metastatic breast cancer, CCL8 was also shown to recruit regulatory T cells (Tregs) through CCR5, and that metastasis in this model was reduced by inhibition of this receptor (Halvorsen et al., 2016). In humans, immunosuppressive CCR8$^+$ Tregs infiltrate breast tumors and CCR8 high expression is correlated with poor prognosis (Plitas et al., 2016). Given these data, we propose that TAM-synthesized CCL8 will increase monocyte infiltration into the tumor site, thus generating more pro-tumoral TAMs (Figure 7) and an immunosuppressive microenvironment, as well as increasing the malignancy of tumor cells. Consistent with these data, SIGLEC1 and CCL8 were associated with shorter disease-specific and recurrence-free survival in public datasets derived from whole tumor homogenates. Such data reinforce the concept of TAMs in the promotion of human malignancy, and identification of uniquely expressed genes in human TAMs provides opportunities for new therapeutic targets and diagnostic/prognostic markers.

STAR★METHODS

Detailed methods are provided in the online version of this paper and include the following:

- KEY RESOURCES TABLE
- CONTACT FOR REAGENT AND RESOURCE SHARING
- EXPERIMENTAL MODELS AND SUBJECT DETAILS
  - Human Studies
- METHOD DETAILS
  - Isolation of Human Blood Monocytes
  - Isolation of Human Tissue Macrophages
  - Monocyte-Derived Macrophages Isolation and Stimulation
  - iPSC Derived Macrophages
  - THP-1 Monocyte Differentiation and Cytokine Stimulation
  - Cancer Cell Culture, Conditioned Medium Production and Cytokine Stimulation
  - Flow Cytometry - Sorting and Analysis
  - RNA Extraction and Sequencing of Purified Cells
  - Semi-quantitative PCR
  - Immunofluorescence and Quantitation
  - Multiplex Immunohistochemistry
  - Antibodies Used for Multiplex IHC
  - ELISAs
  - Cytokine Array
  - iPSDM-Cancer Cell Conditioned Medium Production
  - TNFα Neutralization in Conditioned Medium
  - PCR Arrays

- Cell Proliferation Assay
- *In Vitro* Cell Migration Assay
- Chemotaxis Assay

- QUANTIFICATION AND STATISTICAL ANALYSIS
  - Sequencing Alignment and Quantification
  - Statistical Analysis for RNA-seq Data
  - Enrichment and Pathway Analysis
  - Recursive Feature Elimination with Random Forest
  - Publically Available Datasets
  - TAM Signature
  - Survival Analysis
  - RNA-seq of Total Tissue Breast Cancer
  - Statistical Analysis
- DATA AND SOFTWARE AVAILABILITY

SUPPLEMENTAL INFORMATION

Supplemental Information can be found online at https://doi.org/10.1016/j.ccell.2019.02.009.

ACKNOWLEDGMENTS

This research was supported by Wellcome Trust (101067/Z/13/Z), MRC Center grant MR/N022556/1, and NIH grant PO1 CA100324 to J.W.P., Department of Defense Breast Cancer Research Program (W81XWH-111-0702) to L.M.C. and J.W.P., Susan G Komen Breast Cancer Foundation (KG111084 and KG110560) to E.S.H. and L.M.C., from Breast Cancer Now to A.H.S. and J.M.D., from CONACYT to M.L.-Y. and Wellcome Trust (102610) to A.S. The work was supported extensively by the Edinburgh Breast Unit Team, and particularly by Lorna Renshaw and Jane Keys in this unit and the Departments of Gynecological and Surgical Oncology at the Montefiore Medical Center. We would like to thank the CIR blood resource (AMREC no. 15-HV-013) for the recruitment of blood from normal controls and the CIR flow facility (Shonna Johnston, Will Ramsay, and Mari Pattinson). We thank Dr Dahlia Doughty Shenton and the EPAC lab for InCucyte access. We thank Dr. Samanta A. Mariani for the scientific discussions and suggestions. We thank all the patients and volunteers who contributed to this study as well as all the clinical support teams.

AUTHOR CONTRIBUTIONS

Conceptualization, L.C., S.F., L.M.C., H.O.S., and J.W.P.; Methodology, L.C., S.F., and J.W.P.; Writing – Original Draft, L.C., S.F., and J.W.P.; Writing – Review & Editing, L.C., S.F., L.M.C., and J.W.P.; Formal Analysis, L.C., S.F., D.Y.H.S., P.A., and P.T.S.; Investigation, L.C., S.F., D.Y.H.S., L.M.F., H.Z., A.S., E.Y.L., T.C., A.F., M.L.-Y., M.R.M., and Z.A.; Resources, L.M.F., L.W., E.S.H., H.O.S., A.U., and J.M.D.; Data Curation, S.F., A.H.S., and D.Y.H.S.; Visualization, S.F., L.C., L.M.C., and J.W.P.; Supervision, J.W.P. and A.H.S.; Project Administration, J.W.P.; Funding Acquisition, L.M.C., E.S.H., A.H.S., J.M.D., A.S., M.L.-Y., and J.W.P.

DECLARATION OF INTERESTS

All the authors declare that they have no financial interests. L.M.C. is a paid consultant for Cell Signaling Technologies, received reagent and/or research support from Plexxikon, Deciphera Pharmaceuticals, Pharma, and NanoString Technologies, and is a member of the Scientific Advisory Boards of Syndax Pharmaceuticals, Carisma Therapeutics, and Verseau Therapeutics. L.C., S.F., and J.W.P. are applying for patent protection for data contained in the paper and L.C. and J.W.P. have formed a company in order to exploit these patents.

Received: October 2, 2017
Revised: November 16, 2018
Accepted: February 25, 2019
Published: March 28, 2019

REFERENCES

Anders, S., Pyl, P.T., Huber, W., 2014. HTSeq - a Python framework to work with high-throughput sequencing data. https://doi.org/10.1101/002824.

Andrews, S. (2012). FastQC: a quality control application for high throughput sequence data. https://www.bioinformatics.babraham.ac.uk/projects/fastqc/.

Arwert, E.N., Harney, A.S., Entenberg, D., Wang, Y., Sahai, E., Pollard, J.W., and Condeelis, J.S. (2018). A unidirectional transition from migratory to perivascular macrophage is required for tumor cell intravasation. Cell Rep. *23*, 1239–1248.

Asano, K., Takahashi, N., Ushiki, M., Monya, M., Aihara, F., Kuboki, E., Moriyama, S., Iida, M., Kitamura, H., Qiu, C.-H., et al. (2015). Intestinal CD169(+) macrophages initiate mucosal inflammation by secreting CCL8 that recruits inflammatory monocytes. Nat. Commun. *6*, 7802.

Barbai, T., Fejős, Z., Puskas, L.G., Tímár, J., and Rásó, E. (2015). The importance of microenvironment: the role of CCL8 in metastasis formation of melanoma. Oncotarget *6*, 29111–29128.

Beck, A.H., Espinosa, I., Edris, B., Li, R., Montgomery, K., Zhu, S., Varma, S., Marinelli, R.J., van de Rijn, M., and West, R.B. (2009). The macrophage colony-stimulating factor 1 response signature in breast carcinoma. Clin. Cancer Res. *15*, 778–787.

Bindea, G., Mlecnik, B., Tosolini, M., Kirilovsky, A., Waldner, M., Obenauf, A.C., Angell, H., Fredriksen, T., Lafontaine, L., Berger, A., et al. (2013). Spatiotemporal dynamics of intratumoral immune cells reveal the immune landscape in human cancer. Immunity *39*, 782–795.

Bolger, A.M., Lohse, M., and Usadel, B. (2014). Trimmomatic - a flexible trimmer for Illumina sequence data. Bioinformatics *30*, 2114–2120.

Breiman, L. (2001). Random forests. Mach. Learn. *45*, 5–32.

Cassetta, L., Kajaste-Rudnitski, A., Coradin, T., Saba, E., Della Chiara, G., Barbagallo, M., Graziano, F., Alfano, M., Cassol, E., Vicenzi, E., and Poli, G. (2013). M1 polarization of human monocyte-derived macrophages restricts pre and postintegration steps of HIV-1 replication. AIDS *27*, 1847–1856.

Cassetta, L., Noy, R., Swierczak, A., Sugano, G., Smith, H., Wiechmann, L., and Pollard, J.W. (2016). Isolation of mouse and human tumor-associated macrophages. In Tumor Microenvironment, Advances in Experimental Medicine and Biology (Springer International), pp. 211–229, https://doi.org/10.1007/978-3-319-26666-4_12.

Cassetta, L., and Pollard, J.W. (2018). Targeting macrophages: therapeutic approaches in cancer. Nat. Rev. Drug Discov. *17*, 887–904.

Charoentong, P., Finotello, F., Angelova, M., Mayer, C., Efremova, M., Rieder, D., Hackl, H., and Trajanoski, Z. (2017). Pan-cancer immunogenomic analyses reveal genotype-immunophenotype relationships and predictors of response to checkpoint blockade. Cell Rep. *18*, 248–262.

Chávez-Galán, L., Olleros, M.L., Vesin, D., and Garcia, I. (2015). Much more than M1 and M2 macrophages, there are also CD169+ and TCR+ macrophages. Front. Immunol. *6*, 263.

Chittezhath, M., Dhillon, M.K., Lim, J.Y., Laoui, D., Shalova, I.N., Teo, Y.L., Chen, J., Kamaraj, R., Raman, L., Lum, J., et al. (2014). Molecular profiling reveals a tumor-promoting phenotype of monocytes and macrophages in human cancer progression. Immunity *41*, 815–829.

Curtis, C., Shah, S.P., Chin, S.-F., Turashvili, G., Rueda, O.M., Dunning, M.J., Speed, D., Lynch, A.G., Samarajiwa, S., Yuan, Y., et al. (2012). The genomic and transcriptomic architecture of 2,000 breast tumours reveals novel subgroups. Nature *486*, 346–352.

Dobin, A., Davis, C.A., Schlesinger, F., Drenkow, J., Zaleski, C., Jha, S., Batut, P., Chaisson, M., and Gingeras, T.R. (2012). STAR: ultrafast universal RNA-seq aligner. Bioinformatics *29*, 15–21.

Farmaki, E., Chatzistamou, I., Kaza, V., and Kiaris, H. (2016). A CCL8 gradient drives breast cancer cell dissemination. Oncogene *35*, 6309–6318.

Fang, W., Jokar, I., Zou, A., Lambert, D., Dendukuri, P., and Cheng, N. (2012). CCL2/CCR2 chemokine signaling coordinates survival and motility of breast cancer cells through Smad3 and p42/44MAPK dependent mechanisms. J. Biol. Chem. *287*, 36593–36608.

Feng, A.-L., Zhu, J.-K., Sun, J.-T., Yang, M.-X., Neckenig, M.R., Wang, X.-W., Shao, Q.-Q., Song, B.-F., Yang, Q.-F., Kong, B.-H., and Qu, X. (2011). CD16+ monocytes in breast cancer patients: expanded by monocyte chemoattractant protein-1 and may be useful for early diagnosis. Clin. Exp. Immunol. *164*, 57–65.

Finak, G., Bertos, N., Pepin, F., Sadekova, S., Souleimanova, M., Zhao, H., Chen, H., Omeroglu, G., Meterissian, S., Omeroglu, A., et al. (2008). Stromal gene expression predicts clinical outcome in breast cancer. Nat. Med. *14*, 518–527.

Franklin, R.A., Liao, W., Sarkar, A., Kim, M.V., Bivona, M.R., Liu, K., Pamer, E.G., and Li, M.O. (2014). The cellular and molecular origin of tumor-associated macrophages. Science *344*, 921–925.

Gentles, A.J., Newman, A.M., Liu, C.L., Bratman, S.V., Feng, W., Kim, D., Nair, V.S., Xu, Y., Khuong, A., Hoang, C.D., et al. (2015). The prognostic landscape of genes and infiltrating immune cells across human cancers. Nat. Med. *21*, 938–945, https://doi.org/10.1038/nm.3909.

Halvorsen, E.C., Hamilton, M.J., Young, A., Wadsworth, B.J., LePard, N.E., Lee, H.N., Firmino, N., Collier, J.L., and Bennewith, K.L. (2016). Maraviroc decreases CCL8-mediated migration of CCR5 regulatory T cells and reduces metastatic tumor growth in the lungs. OncoImmunology *5*, 1–15.

Hanna, R.N., Cekic, C., Sag, D., Tacke, R., Thomas, G.D., Nowyhed, H., Herrley, E., Rasquinha, N., McArdle, S., Wu, R., et al. (2015). Patrolling monocytes control tumor metastasis to the lung. Science *350*, 985–990.

Huang da, W., Sherman, B.T., and Lempicki, R.A. (2009). Systematic and integrative analysis of large gene lists using DAVID bioinformatics resources. Nat. Protoc. *4*, 44–57.

Johnson, W.E., Li, C., and Rabinovic, A. (2007). Adjusting batch effects in microarray expression data using empirical Bayes methods. Biostatistics *8*, 118–127.

Jung, K., Heishi, T., Khan, O.F., Kowalski, P.S., Incio, J., Rahbari, N.N., Chung, E., Clark, J.W., Willett, C.G., Luster, A.D., et al. (2017). Ly6Clo monocytes drive immunosuppression and confer resistance to anti-VEGFR2 cancer therapy. J. Clin. Invest. *127*, 3039–3051.

Karnoub, A.E., Dash, A.B., Vo, A.P., Sullivan, A., Brooks, M.W., Bell, G.W., Richardson, A.L., Polyak, K., Tubo, R., and Weinberg, R.A. (2007). Mesenchymal stem cells within tumour stroma promote breast cancer metastasis. Nature *449*, 557–563.

Kim, D., Pertea, G., Trapnell, C., Pimentel, H., Kelley, R., and Salzberg, S.L. (2013). TopHat2: accurate alignment of transcriptomes in the presence of insertions, deletions and gene fusions. Genome Biol. *14*, R36.

Kitamura, T., Qian, B., Soong, D., Cassetta, L., Noy, R., Sugano, G., Kato, Y., Li, J., and Pollard, J.W. (2015). CCL2-induced chemokine cascade promotes breast cancer metastasis by enhancing retention of metastasis-associated macrophages. J. Exp. Med. *212*, 1043–1059.

Knüpfer, H., and Preiß, R. (2006). Significance of interleukin-6 (IL-6) in breast cancer (review). Breast Cancer Res. Treat. *102*, 129–135.

Kuhn, M., 2015. Caret: classification and regression training. Astrophysics source code library. http://adsabs.harvard.edu/abs/2015ascl.soft05003K.

Leek, J.T. (2014). svaseq: removing batch effects and other unwanted noise from sequencing data. Nucleic Acids Res. *42*, https://doi.org/10.1093/nar/gku864.

Li, C., Luo, X., Lin, Y., Tang, X., Ling, L., Wang, L., and Jiang, Y. (2015). A higher frequency of CD14+ CD169+ monocytes/macrophages in patients with colorectal cancer. PLoS One *10*, e0141817, https://doi.org/10.1371/journal.pone.0141817.

Liang, C.-C., Park, A.Y., and Guan, J.-L. (2007). In vitro scratch assay: a convenient and inexpensive method for analysis of cell migration in vitro. Nat. Protoc. *2*, 329–333.

Lopez-Yrigoyen, M., Fidanza, A., Cassetta, L., Axton, R.A., Taylor, A.H., Meseguer-Ripolles, J., Tsakiridis, A., Wilson, V., Hay, D.C., Pollard, J.W., and Forrester, L.M. (2018). A human iPSC line capable of differentiating into functional macrophages expressing ZsGreen: a tool for the study and in vivo tracking of therapeutic cells. Philos. Trans. R. Soc. Lond. B Biol. Sci. *373*, https://doi.org/10.1098/rstb.2017.0219.

Makita, N., Hizukuri, Y., Yamashiro, K., Murakawa, M., and Hayashi, Y. (2015). IL-10 enhances the phenotype of M2 macrophages induced by IL-4 and confers the ability to increase eosinophil migration. Int. Immunol. *27*, 131–141.

Martinez, F.O., Gordon, S., Locati, M., and Mantovani, A. (2006). Transcriptional profiling of the human monocyte-to-macrophage differentiation and polarization: new molecules and patterns of gene expression. J. Immunol. *177*, 7303–7311.

Neubert, N.J., Schmittnaegel, M., Bordry, N., Nassiri, S., Wald, N., Martignier, C., Tillé, L., Homicsko, K., Damsky, W., Maby-El Hajjami, H., et al. (2018). T cell-induced CSF1 promotes melanoma resistance to PD1 blockade. Sci. Transl. Med. *10*, https://doi.org/10.1126/scitranslmed.aan3311.

Neve, R.M., Chin, K., Fridlyand, J., Yeh, J., Baehner, F.L., Fevr, T., Clark, L., Bayani, N., Coppe, J.-P., Tong, F., et al. (2006). A collection of breast cancer cell lines for the study of functionally distinct cancer subtypes. Cancer Cell *10*, 515–527.

Parker, J.S., Mullins, M., Cheang, M.C.U., Leung, S., Voduc, D., Vickery, T., Davies, S., Fauron, C., He, X., Hu, Z., et al. (2009). Supervised risk predictor of breast cancer based on intrinsic subtypes. J. Clin. Oncol. *27*, 1160–1167.

Pearce, D.A., Nirmal, A.J., Freeman, T.C., and Sims, A.H. (2017). Continuous biomarker assessment by exhaustive survival analysis. bioRxiv, 208660, https://doi.org/10.1101/208660.

Planet, E., 2013. phenoTest: tools to test association between gene expression and phenotype in a way that is efficient, structured, fast and scalable. We also provide tools to do GSEA (Gene set enrichment analysis) and copy number variation. R package version 1.28.0. https://doi.org/10.18129/B9.bioc.phenoTest.

Plitas, G., Konopacki, C., Wu, K., Bos, P.D., Morrow, M., Putintseva, E.V., Chudakov, D.M., and Rudensky, A.Y. (2016). Regulatory T cells exhibit distinct features in human breast cancer. Immunity *45*, 1122–1134.

Qian, B., Li, J., Zhang, H., Kitamura, T., Zhang, J., Campion, L.R., Kaiser, E.A., Snyder, L.A., and Pollard, J.W. (2011). CCL2 recruits inflammatory monocytes to facilitate breast-tumour metastasis. Nature *475*, 222–225.

Ritchie, M.E., Phipson, B., Wu, D., Hu, Y., Law, C.W., Shi, W., and Smyth, G.K. (2015). Limma powers differential expression analyses for RNA-sequencing and microarray studies. Nucleic Acids Res. *43*, e47.

Roberts, A., Trapnell, C., Donaghey, J., Rinn, J.L., and Pachter, L. (2011). Improving RNA-Seq expression estimates by correcting for fragment bias. Genome Biol. *12*, R22.

Robinson, M.D., and Oshlack, A. (2010). A scaling normalization method for differential expression analysis of RNA-seq data. Genome Biol. *11*, R25.

Roomi, M.W., Monterrey, J.C., Kalinovsky, T., Rath, M., and Niedzwiecki, A. (2009). Patterns of MMP-2 and MMP-9 expression in human cancer cell lines. Oncol. Rep. *21*, 1323–1333.

Shin, S.Y., Lee, D.H., Lee, J., Choi, C., Kim, J.-Y., Nam, J.-S., Lim, Y., and Lee, Y.H. (2017). C-C motif chemokine receptor 1 (CCR1) is a target of the EGF-AKT-mTOR-STAT3 signaling axis in breast cancer cells. Oncotarget *8*, 94591–94605.

Sing, T., Sander, O., Beerenwinkel, N., and Lengauer, T. (2005). ROCR: visualizing classifier performance in R. Bioinformatics *21*, 3940–3941.

Takeya, M., and Komohara, Y. (2016). Role of tumor-associated macrophages in human malignancies: friend or foe? Pathol. Int. *66*, 491–505.

Tamborero, D., Rubio-Perez, C., Muiños, F., Sabarinathan, R., Piulats, J.M., Muntasell, A., Dienstmann, R., Lopez-Bigas, N., and Gonzalez-Perez, A. (2018). A pan-cancer landscape of interactions between solid tumors and infiltrating immune cell populations. Clin. Cancer Res. *24*, 3717–3728.

Tóth, Z.E., and Mezey, E. (2007). Simultaneous visualization of multiple antigens with tyramide signal amplification using antibodies from the same species. J. Histochem. Cytochem. *55*, 545–554.

Trapnell, C., Williams, B.A., Pertea, G., Mortazavi, A., Kwan, G., van Baren, M.J., Salzberg, S.L., Wold, B.J., and Pachter, L. (2010). Transcript assembly and quantification by RNA-Seq reveals unannotated transcripts and isoform switching during cell differentiation. Nat. Biotechnol. *28*, 511–515.

Tsujikawa, T., Kumar, S., Borkar, R.N., Azimi, V., Thibault, G., Chang, Y.H., Balter, A., Kawashima, R., Choe, G., Sauer, D., et al. (2017). Quantitative multiplex immunohistochemistry reveals myeloid-inflamed tumor-immune complexity associated with poor prognosis. Cell Rep. *19*, 203–217.

Wang, X., Spandidos, A., Wang, H., and Seed, B. (2012). PrimerBank: a PCR primer database for quantitative gene expression analysis, 2012 update. Nucleic Acids Res. *40*, D1144–D1149.

Williams, C.B., Yeh, E.S., and Soloff, A.C. (2016). Tumor-associated macrophages: unwitting accomplices in breast cancer malignancy. NPJ Breast Cancer *2*, 15025.

Yang, C.-T., Ma, R., Axton, R.A., Jackson, M., Taylor, A.H., Fidanza, A., Marenah, L., Frayne, J., Mountford, J.C., and Forrester, L.M. (2017). Activation of KLF1 enhances the differentiation and maturation of red blood cells from human pluripotent stem cells. Stem Cells *35*, 886–897, https://doi.org/10.1002/stem.2562.

Yang, M., McKay, D., Pollard, J.W., and Lewis, C.E. (2018). Diverse functions of macrophages in different tumor microenvironments. Cancer Res. *78*, 5492–5503.

Zhang, Y., Li, J.-Q., Jiang, Z.-Z., Li, L., Wu, Y., and Zheng, L. (2016). CD169 identifies an anti-tumour macrophage subpopulation in human hepatocellular carcinoma. J. Pathol. *239*, 231–241.

STAR★METHODS

KEY RESOURCES TABLE

REAGENT or RESOURCE	SOURCE	IDENTIFIER
Antibodies		
Anti CD45 PE-Texas Red clone HI30	Thermofisher	Cat# MHCD4517; RRID: AB_10372514
Anti CD45 AF700 clone HI30	Biolegend	Cat# 304024; RRID: AB_493761
Anti CD45 clone HI30	eBioscience	Cat# 14-0459-82; RRID: AB_467274
Anti CD3 BV711 clone OKT3	Biolegend	Cat# 317328; RRID: AB_2562907
Anti CD3 Pe-Cy5 clone UCHT1	Biolegend	Cat# 300410; RRID: AB_314064
Anti CD3 clone SP7	Thermofisher	Cat# MA1-90582; RRID: AB_1956722
Anti CD56 BV711 clone HCD56	Biolegend	Cat# 318336; RRID: AB_2562417
Anti CD56 PE-Cy5 clone HCD56	Biolegend	Cat# 318308; RRID: AB_604105
Anti CD56 clone 123C3	Santa Cruz	Cat# sc-7326; RRID: AB_627127
Anti CD19 BV711 clone HIB19	Biolegend	Cat# 302246; RRID: AB_2562065
Anti CD19 PE-Cy5 clone HIB19	Biolegend	Cat# 302210; RRID: AB_314240
Anti CD11b BV605 clone ICRF44	Biolegend	Cat# 301332; RRID: AB_2562021
Anti CD11b PE-Cy7 clone ICRF44	Thermofisher	Cat# 25-0118-42; RRID: AB_1582272
Anti CD14 BV510 clone M5E2	Biolegend	Cat# 301842; RRID: AB_2561946
Anti CD14 FITC clone TuK4	Thermofisher	Cat# MHCD1401; RRID: AB_10373108
Anti CD16 EF450 clone eBIOCB16	Thermofisher	Cat# 48-0168-42; RRID: AB_1272052
Anti CD16 PE-Texas Red clone 3G8	Thermofisher	Cat# MHCD1617; RRID: AB_10373685
Anti HLA-DR BV650 clone L243	Biolegend	Cat# 307650; RRID: AB_2563828
Anti CX3CR1 FITC clone 2A9-1	Biolegend	Cat# 341606; RRID: AB_1626272
Anti CD64 AP-CCy7 clone 10.1	Biolegend	Cat# 305026; RRID: AB_2561588
Anti CD80 PE-Cy7 clone 2D10	Biolegend	Cat# 305218; RRID: AB_2076148
Anti CD86 APC clone IT2.2	Biolegend	Cat# 305412; RRID: AB_493231
Anti CD163 APC clone GH1/61	Biolegend	Cat# 333610; RRID: AB_2074533
Anti CD163 clone 10D6	Thermofisher	Cat# MA5-11458; RRID: AB_10982556
Anti CD163 clone 10D6	Leica Biosystems	Cat# NCL-L-CD163; RRID: AB_2756375
Anti CCR2 PE-Cy7 clone K036C2	Biolegend	Cat# 357212; RRID: AB_2562619
Anti CCR2 clone 48607	R&D Systems	Cat# MAB150; RRID: AB_2247178
Anti CD169 PE clone 7-239	Biolegend	Cat# 346003; RRID: AB_2189030
Anti CD169 clone 5F1.1	Millipore	Cat# MABT328
Anti CD169 polyclonal	Novus Biologicals	Cat# NBP2-30903
Anti CD8 clone C8/144B	Thermofisher	Cat# MA5-13473; RRID: AB_11000353
Anti CSF1R clone SP211	Abcam	Cat# ab183316
Anti CD95 PE-Cy7 clone DX2	Biolegend	Cat# 305622; RRID: AB_2100369
Anti CCR1 PE clone 5F10B29	Biolegend	Cat# 362903; RRID: AB_2563897
Anti CCR3 FITC clone 5E8	Biolegend	Cat# 310719; RRID: AB_2571958
Anti CCR5 PE clone HEK/1/85a	Biolegend	Cat# 313707; RRID: AB_345307
Anti CCR8 PE clone L263G8	Biolegend	Cat# 360603; RRID: AB_2562614
Anti TNFα clone 1825	R&D Systems	Cat# MAB210; RRID: AB_2240620
Mouse IgG_1 isotype control	R&D Systems	Cat# MAB002; RRID: AB_357344
Goat anti rabbit Peroxidase F(ab)	Abcam	Cat# ab7171; RRID: AB_955396
Chemicals, Peptides, and Recombinant Proteins		
Recombinant human IL3	PreproTech	0200-03-10
Recombinant human IL4	R&D Systems	204-IL
Recombinant human IL10	R&D Systems	217-IL

(*Continued on next page*)

Continued		
REAGENT or RESOURCE	SOURCE	IDENTIFIER
Recombinant human IL13	R&D Systems	213-ILB
Recombinant human CCL2	R&D Systems	279-MC
Recombinant human CCL8	R&D Systems	281-CP
Recombinant human CSF1	Biolegend	574806
Recombinant human BMP4	R&D Systems	314-BP-010
Recombinant human SCF	Thermo Fisher	PHC2111
Recombinant human VEGF 165	R&D Systems	293-VE-010
Recombinant human TGFβ	R&D Systems	240-B
Recombinant human IFNγ	R&D Systems	285-IF
Recombinant human TNFα	R&D Systems	210-TA
Recombinant human Basic FGF (aa 10-155)	Thermo Fisher	PHG0021
Phorbol-12-myristate-13-acetate (PMA)	Sigma Aldrich	16561-29-8
Liberase enzyme TL	Roche	5401020001
Liberase enzyme DL	Roche	5401160001
Dnase I	Sigma Aldrich	11284932001
StemPro® hESC SFM	Invitrogen	A1000701
Critical Commercial Assays		
RNAEasy Microkit	Qiagen	74004
SuperScript Vilo master mix	Thermofisher	11755050
Human CCL8 DuoSet ELISA	R&D Systems	DY281
Human TNFα DuoSet ELISA	R&D Systems	DY210
Human IL1β DuoSet ELISA	R&D Systems	DY201
Human CX3CL1 Quantikine ELISA kit	R&D Systems	DCX310
Human CSF1 Quantikine ELISA kit	R&D Systems	DMC00B
Human Cytokine ELISA array (colorimetric)	Signosis	EA-4002
Human Proinflammatory chemokine Legendplex	Biolegend	740003
Cell counting kit-8	Sigma Aldrich	96992
Human Breast Cancer RT^2 Profiler PCR Array	Qiagen	PAHS-131Z
RT^2 Profiler™ PCR Array Human Inflammatory Cytokines & Receptors	Qiagen	PAHS-011Z
Human Tumor Metastasis RT^2 Profiler PCR Array	Qiagen	PAHS-028Z
RNAscope 2.5 LS Reagent Kit	ACD	322100
RNAscope 2.5 LS Probe-Hs-PPIB	ACD	313908
RNAscope 2.5 LS Probe-Hs-CCL8	ACD	466498
IncuCyte® ClearView 96-Well Chemotaxis Plate	Essen bioscience	4582
TSA Plus Cyanine 3 system	Perkin Elmer	NEL774B001KT
Deposited Data		
TEMo and TAM RNA-seq (Cohort 1 and 2)	This paper	GSE117970
Breast cancer tissue RNA-seq (Cohort 3)	This paper	GSE100925
Microarray data from human healthy and breast cancer stroma	Karnoub et al., 2007	GSE8977
Microarray data from human healthy and breast cancer stroma	Finak et al., 2008	GSE9014
Microarray data from the METABRIC cohort	Curtis et al., 2012	http://www.cbioportal.org/
RNA-seq samples from the Cancer cell Encyclopedia (CCLE)	N/A	https://portals.broadinstitute.org/ccle
Experimental Models: Cell Lines		
MDA-MB-231	ATCC	Cat# HTB-26; RRID: CVCL_0062
MDA-MB-468	ATCC	Cat# HTB-132; RRID: CVCL_0419

(*Continued on next page*)

Continued		
REAGENT or RESOURCE	SOURCE	IDENTIFIER
THP-1	ATCC	Cat# TIB-202; RRID: CVCL_000
SFCi55 iPSC	Prof. Lesley Forrester	Lopez-Yrigoyen et al., 2018
Oligonucleotides		
GAPDH Forward	Sigma Aldrich	5'- GGAGCGAGATCCCTCCAAAAT-3'
GAPDH Reverse	Sigma Aldrich	5'- GGCTGTTGTCATACTTCTCATGG-3'
HGF Forward	Sigma Aldrich	5'- GCTATCGGGGTAAAGACCTACA-3'
HGF Reverse	Sigma Aldrich	5'- CGTAGCGTACCTCTGGATTGC-3'
TNFSF10 Forward	Sigma Aldrich	5'- TGCGTGCTGATCGTGATCTTC-3'
TNFSF10 Reverse	Sigma Aldrich	5'- GCTCGTTGGTAAAGTACACGTA-3'
ANGPT1 Forward	Sigma Aldrich	5'-AGAACCTTCAAGGCTTGGTTAC-3'
ANGPT1 Reverse	Sigma Aldrich	5'-GGTGGTAGCTCTGTTTAATTGCT-3'
CD200R1 Forward	Sigma Aldrich	5'-CAGAGGCATAGTGGTAACACCT-3'
CD200R1 Reverse	Sigma Aldrich	5'-GTGCCATTGCCCCAGTATTCT-3'
SIGLEC1 Forward	Sigma Aldrich	5'-CCTCGGGGAGGAACATCCTT-3'
SIGLEC1 Reverse	Sigma Aldrich	5'-AGGCGTACCCCATCCTTGA-3'
CCL8 Forward	Sigma Aldrich	5'-TGGAGAGCTACACAAGAATCACC-3'
CCL8 Reverse	Sigma Aldrich	5'-TGGTCCAGATGCTTCATGGAA-3'
TNFA Forward	Sigma Aldrich	5'-CCTGCTGCACTTTGGAGTGA-3'
TNFA Reverse	Sigma Aldrich	5'-TCGAGAAGATGATCTGACTGCC-3'
Software and Algorithms		
Image J	NIH	https://imagej.nih.gov/ij/
BD Facs Diva version 8	BD Biosciences	http://www.bdbiosciences.com/
FlowJo Version 9 and 10	FlowJo LLC	https://www.flowjo.com/
Prism Version 6 and 7	GraphPad	https://www.graphpad.com/scientific-software/prism
Mathematica 11	Wolfram Inc.	http://www.wolfram.com/mathematica/
Tissue Studio 2.7	Definiens AG	https://www.definiens.com/
Developer XD 2.7	Definiens AG	https://www.definiens.com/
Zen	Carl Zeiss AG	https://www.zeiss.com/microscopy/int/products/microscope-software/zen.html
IncuCyte software	Essen Biosciences	https://www.essenbioscience.com/
FastQC	(Andrews, 2012)	https://www.bioinformatics.babraham.ac.uk/projects/fastqc/
STAR 2.3	(Dobin et al., 2012)	https://github.com/alexdobin/STAR
HTSeq	(Anders et al., 2014)	https://htseq.readthedocs.io/en/release_0.9.1/
DAVID	(Huang da et al., 2009)	https://david.ncifcrf.gov/
Trimmomatic	(Bolger et al., 2014)	http://www.usadellab.org/cms/?page=trimmomatic
TopHat 2.0.12	(Kim et al., 2013)	https://ccb.jhu.edu/software/tophat/index.shtml
Cufflinks	(Trapnell et al., 2010)	http://cole-trapnell-lab.github.io/cufflinks/

CONTACT FOR REAGENT AND RESOURCE SHARING

Further information and requests for resources and reagents should be directed to and will be fulfilled by the Lead Contact, Jeffrey W. Pollard (jeff.pollard@ed.ac.uk).

EXPERIMENTAL MODELS AND SUBJECT DETAILS

Human Studies

All study protocols were approved by the IRB of the Albert Einstein Medical College (Bronx, NY, USA), by The University of Edinburgh (Edinburgh, UK) and Duke University (Durham, NC) ethics committees as appropriate. Informed consent was obtained from all human subjects included in this study.

Cohort 1: For control samples, mononuclear cells were isolated from peripheral blood obtained from female healthy individuals through the New York Blood Center, USA. In some cases, blood was also donated from volunteers in the Bronx, NY who were

age and weight matched to the Bronx cancer cohort. Peripheral blood (20 ml) was obtained from breast and endometrial cancer patients attending the Montefiore Medical Center, Bronx, NY, USA. Breast cancer tissue (0.1-1 grams) and endometrial cancer tissue (0.1-1 grams) was obtained from Montefiore Medical Center, NY, USA. Normal breast tissue from mammoplasty reduction surgeries (25-50 grams) was obtained from the Human Tissue Procurement Facility (HTPF), Ohio State University, USA; normal/benign endometrial tissue (1-2 grams) was obtained after surgery for conditions unrelated to cancer from Montefiore Medical Center, NY, USA.

Cohort 2: For control samples, mononuclear cells were isolated from peripheral blood obtained from female healthy individuals through Cambridge Biosciences, UK or CIR blood resource (AMREC #15-HV-013). Peripheral blood (20 ml) and cancer tissue (0.1-1 grams) was obtained from breast cancer patients from NHS, Edinburgh, Scotland, UK. Normal/benign breast tissue (0.1-1 grams) from patients with benign conditions was obtained from NHS, Edinburgh, Scotland, UK.

Cohort 3: Breast cancer tissue was obtained by Duke University, Durham NC, USA. Pathologically the breast cancer patients consisted of invasive breast cancers with either node$^-$ or node$^+$ disease. Patients had biopsy-confirmed invasive tumors of at least 1.5 cm at diagnosis. Tumor samples were shipped on ice to Oregon Health & Science University Hospital (OHSU) for immune and genomic assays.

The exclusion criteria for all cancer patients at baseline included systemic metastatic disease, any inflammatory disorder, and active infection or immunocompromised status not related to cancer. All the patients recruited were chemotherapy and radiotherapy naive before collection.

METHOD DETAILS

Isolation of Human Blood Monocytes

All blood samples were collected and processed by the same person according to site, HZ in the Bronx and LC Edinburgh. They were processed as attained and not batched together according to sample type. All the blood samples were collected in Venous Blood Collection Tubes containing EDTA and stored immediately at 4°C after collection. Blood was centrifuged at 700 RCF for 10 min at 4°C in a swinging bucket rotor to separate cells from plasma, Plasma was then subjected to centrifugation in conical tubes for 10 min at 16,000 x g at 4°C in a fixed angled rotor, immediately aliquoted and stored at -80°C. After red blood cell lysis (10X RBC lysis buffer, Biolegend) cells were centrifuged 500 RCF 5 min at 4°C, counted and stained for cytofluorimetric analysis; the remaining cells were frozen in 10% v/v DMSO, 90% v/v fetal bovine serum solution for subsequent cell sorting and RNA extraction.

Isolation of Human Tissue Macrophages

Cancer tissue and normal endometrial tissue were washed with Phosphate Buffer Saline (PBS) in a petri dish and tissue was chopped into small fragments with a razorblade on ice. The sample was transferred to a 15-50 ml tube according to size and Liberase enzymes TL (14 U/mL) and DL (28 U/mL) (Roche) and DNAse (15 mg/mL) (Roche) were added in serum-free PBS. Tissue was digested at 37°C on a rotating wheel for 1-18 hr depending on tissue weight; at the end of digestion the cell suspension was filtered using a 100 μm cell strainer and PBS 1% w/v Bovine Serum Albumin (BSA, Sigma-Aldrich) was added in order to interrupt the digestion process. Cells were centrifuged at 400 RCF for 5 min at 4°C in a swinging bucket rotor. The pellet was re-suspended in PBS, 1%w/v BSA and cells counted and stained for FACS sorting or analysis. Macrophages were sorted using the antibodies CD45 AlexaFluor-700, CD3 PE-Cy5, CD56 PE-Cy5, CD19 PE-Cy5, CD14 FITC, CD11b PE-Cy7, CD163 APC as reported in Figure S2A (Cassetta et al., 2016).

Monocyte-Derived Macrophages Isolation and Stimulation

Peripheral blood was collected from healthy donors in EDTA coated blood tubes and diluted 1:2 using serum free PBS. 40 mL of the diluted blood was then stratified on top of 10 mL of Ficoll; samples were centrifuged at 400 RCF (no brake, no acceleration) for 30 min at room temperature (RT) in a swinging bucket rotor. The peripheral mononuclear cell (PBMC) fraction (ring) was collected with a pipette and cells washed with PBS (Cassetta et al., 2013). PBMC were counted and seeded in a 12-well plate (NUNC-BD) at the concentration of $8x10^6$ cells/ml for 2 hr at 37°C 5% v/v CO_2 in serum free medium (Dulbecco's Modified Eagle Medium, DMEM). Non-adherent cells were removed and wells washed twice with PBS and 2 ml of DMEM 10% v/v Fetal Bovine Serum (Lonza), 5% v/v Human AB serum (Lonza) and 1% v/v penicillin/streptomycin were added to each well; 50% of the medium (1.0 ml) was replaced with fresh medium every 3 days. After 7 days of differentiation monocyte-derived macrophages (MDM) were treated for 24 hr with MDA-MB-231 and MDA-MB-468 cancer cell derived supernatant (CM) as reported in the below sections (Kitamura et al., 2015). After 24 hr all the supernatant was removed and used for quantitative real-time (qPCR) metastasis breast cancer array (see below), cells were washed twice with PBS and lysed with Trizol Reagent (Thermo Fisher) for RNA extraction; RNA was extracted using Trizol manufacturer's protocol. RNA was converted to cDNA using Invitrogen Superscript Vilo cDNA synthesis kit and qPCR was performed using the protocol described above in the text.

iPSC Derived Macrophages

The SFCi55 iPSC line was generated in house and was confirmed to be pluripotent and have a normal karyotype (Yang et al., 2017). The cells were maintained in StemPro medium prepared by supplementing DMEM/F12 + Glutamax (Invitrogen) with StemPro hESC supplement (Invitrogen), 1.8% w/v BSA (Invitrogen), 0.1 mM β-mercaptoethanol (Invitrogen) and 20 ng/ml human basic FGF (Invitrogen). When iPSC colonies covered approximately 80% of the culture surface, (Day 0), spent medium was removed and replaced with 1.0 ml StemPro supplemented with cytokine Mix 1 (50 ng/ml BMP4, 50 ng/ml VEGF, and 20 ng/ml SCF). Colonies

were cut using the EZPassage™ tool, and gently dislodged with a Pasteur pipette. They were divided equally into two wells of an Ultra-Low Attachment 6-well plate (Corning), and 2 ml of fresh StemPro media with cytokine Mix 1. Cells were cultured in suspension until day 4 with a cytokine top up on Day 2, to make embryoid bodies (EBs). On Day 4, EBs were lifted and transferred to gelatin-coated tissue-culture grade 6-well plates in X-VIVOTM 15 media (Lonza) supplemented with cytokine Mix 2 (100 ng/ml CSF1, 25 ng/ml IL3, 2.0 mM Glutamax, 1% v/v Penicillin/Streptomycin, 0.055 M β-mercaptoethanol). 10 to 15 EBs were plated in each well. EBs were maintained in this medium for the remainder of the protocol, with spent medium being replaced with fresh medium every 3-4 days. After about 2-3 weeks, the EBs produced macrophage progenitors in the culture supernatant that were harvested and transferred to 10 cm^2 bacteriological dishes in X-VIVOTM 15 medium supplemented with cytokine Mix 3 (100 ng/ml CSF1, 2.0 mM Glutamax, 1% v/v Penicillin/Streptomycin) and allowed to mature for 7 days into iPSC-derived macrophages (iPSC-DM). Macrophage progenitors were harvested every 4 days for approximately 2 months (Lopez-Yrigoyen et al., 2018).

THP-1 Monocyte Differentiation and Cytokine Stimulation

Human THP-1 monocytes were maintained in culture medium (10% v/v Fetal Bovine Serum [FBS] Roswell Park Memorial Institute [RPMI] 1640 Medium) and incubated at 37°C in a 5% v/v CO_2 atmosphere. For monocyte-macrophage differentiation, cells were seeded in at a density of $2.5x10^5$ cells/ml on 12-well plates, or $5x10^5$ cells/ml in 6-well plates and macrophage differentiation was initiated by exposing the cells to 5ng/ml phorbol-12-myristate-13-acetate (PMA) (Sigma-Aldrich, 16561-29-8) in 10% v/v FBS culture medium at 37°C in a 5% v/v CO_2 atmosphere for 24 hr. Subsequently, THP-1 derived macrophages were polarized using different combinations of IL4, IL10, IL13 and TGFβ (R & D systems) or using different pro-inflammatory cytokines including TNFα, IFNγ IL1β, IL6 and IL12 (R & D systems) and Lipopolysaccharide (LPS). The cytokines doses were 20 ng/ml and LPS was used at 25 ng/ml.

Cancer Cell Culture, Conditioned Medium Production and Cytokine Stimulation

MDA-MB-468 and THP1 cell lines were cultured in RPMI1640 with 10% v/v serum (GIBCO, Life Technologies); MDA-MB-231 cells were cultured in DMEM with 10% v/v serum (GIBCO, Life Technologies). All cells were originally obtained from ATCC (Manassas, VA, USA) and subsequently maintained in our laboratory. All cell lines were frequently tested for mycoplasma contamination using a commercially available Mycoplasma detection kit (Myco alert kit, Lonza, USA), and all tested negative. To obtain MDA-MB-231 and -468 CMs cells were resuspended in culture medium, seeded at a density of $1x10^5$ cells/ml in 2.5 ml culture medium on 6-well plates and cultured overnight at 37°C in a 5% v/v CO_2 atmosphere. Subsequently, for CM exposure on PMA-THP-1 monocytes, culture medium was replaced with 10% v/v FBS RPM1640 medium, for CM exposure on human MDMs, culture medium was replaced with 10% v/v FBS DMEM supplemented with 5% v/v human serum and for CM exposure on human iPSDM culture medium was replaced with 10% v/v FBS DMEM. After medium change, cells were cultured for an additional 24 hr with fresh medium and thereafter, cell free supernatants were harvested and directly used for the experiment.

Flow Cytometry - Sorting and Analysis

PBMCs or total blood cells were counted and re-suspended in PBS, 1%w/v BSA; blocking of Fc receptors was performed by incubating samples with 10% v/v human serum (Sigma Aldrich) for 1 hr on ice. For cytofluorimetric analysis $5x10^5$ cells were stained in a final volume of 100 μL using the following antibodies at 1:100 dilutions: CD45 PE-Texas Red, CD3-, CD56-, CD19-BV711, CD11b BV605, CD14 BV510, CD16 EF450, CX3CR1 FITC, HLA-DR BV650, CCR2 PE-Cy7 (Biolegend). For monocyte and macrophage sorting cells were stained and antibody concentration was scaled up based on cell number; cells were stained with the following antibodies at 1:100 dilutions: CD45-AlexaFluor 700, CD3-, CD56-, CD19- PE-Cy5, CD14 FITC, CD11b PE-Cy7, CD16 PE-Texas Red, CD163 APC (Biolegend). Cancer cell lines were stained for the 5 CCL8 receptors with the following antibodies: CCR1 PE, CCR2 PE-Cy7, CCR3 FITC, CCR5 PE, CCR8 PE (Biolegend). Cells were incubated in the dark for 1 hr on ice; after washing with PBS 1%w/v BSA (analysis) or PBS 0.1%w/v BSA (sorting) cells were filtered and re-suspended in the appropriate buffer before analysis or sorting. Cytofluorimetric analysis was performed using a 6-laser Fortessa flow cytometer (BD); FACS sorting was performed using FACS AriaII and FACS Fusion sorters (BD). Cell sorting was performed at 4°C in 1.5 ml RNAse and DNAse free tubes (Simport, Canada) pre-filled with 750 μl of PBS 0.1%w/v BSA; at the end of each isolation a sorting purity check was performed. A minimum of 5,000 events in the monocyte/macrophage gate was acquired for cytofluorimetric analysis. Results were analyzed with Flowjo (Treestar) or DIVA software (BD) (see Key Resources Table).

RNA Extraction and Sequencing of Purified Cells

Immediately after sorting all the samples were centrifuged at 450 RCF for 10 min at 4°C. The cell pellet was re-suspended in 350 μL of RLT lysis buffer + 1% v/v βmercaptoethanol, and RNA extracted with RNAeasy Microkit (Qiagen) according to manufacturer's instructions. RNA quantity was determined by QUBIT (Invitrogen); total RNA integrity was assessed by Agilent Bioanalyzer and the RNA Integrity Number (RIN) was calculated; samples that had a RIN > 7 were selected for RNA amplification and sequencing. RNA was amplified with Ovation RNA-seq Amplification kit v2 (Nugen) according to manufacturer's instructions. Amplified RNA was sent to Albert Einstein Genomic Facility (https://www.einstein.yu.edu/departments/genetics/resources/genomics-core.aspx) or BGI (https://www.bgi.com/us/) where library preparation, fragmentation and paired-end multiplex sequencing were performed (HiSeq 2000 and 2005, Illumina). All samples were processed and randomly assigned to lanes without knowledge of clinical identity to avoid bias and batch effects.

Semi-quantitative PCR

Cells were lysed and RNA extracted with RNAeasy Microkit (Qiagen) according to manufacturer's instructions. Typically, 0.1 ug of total RNA was reverse transcribed using Super Script Vilo kit (Invitrogen) and the cDNA generated was used for semi quantitative PCR on a 7900 Real Time cycler (Applied Biosystem) as per manufacturer's instructions using SYBR green master mix (ThermoFisher). Target gene expression was normalized to the expression of the housekeeping gene GAPDH. Relative gene expression was calculated using the standard 2-ΔΔCT method. Primers were designed using Primer Bank (Wang et al., 2012). The full list of primers used can be found in Key Resources Table.

Immunofluorescence and Quantitation

All tissues were fixed in 4% w/v paraformaldehyde, dehydrated and embedded in paraffin blocks; 5 μm sections were cut onto positively charged glass slides and stained with the following antibodies: CD163 (Leica Biosystems NCL-LCD163, Clone 10D6) dilution 1:1000, CD169 (Novus Biologicals, NPB2-30903, polyclonal) dilution 1:100. High throughput immunofluorescence was performed by the SURF Facility at the University of Edinburgh (http://surf.ed.ac.uk/facilities/immunodetection-and-histological-imaging/) after primary antibody optimization. Immunofluorescently stained tissues were batch-scanned on a Zeiss AxioScan.Z1 (Carl Zeiss, Oberkochen, Germany) with specific scan profiles for each stain group and using a 40x Plan-Apochromat 0.95NA coverslip corrected air objective. Slide scanned images were imported into a Definiens Tissue Studio workspace (Definiens AG, Munich, Germany) and pre-processed for nuclear detection and cell simulation using built-in nuclear detection and cell growth algorithms. The pre-processed workspace was then imported into Definiens Developer XD (Definiens AG, Munich, Germany) for further processing, quality control, machine learning, and k-Nearest Neighbour classification and output compiled in Mathematica 10.3 (Wolfram Inc., Champaign, Illinois, United States) and tabulated in a spreadsheet. Incomplete or low-quality nuclei and cells were discarded using a combination of DAPI pixel intensities and standard deviation. For CD163, examples of 300 cells each were given for positive and negative cases in a single large tissue sub-region of one cancer tissue previously identified to show the most variation of intensity. These class samples were used to optimize a feature space consisting of 49 subjectively selected morphological, textural, statistical, and intensity-based metrics. Feature space optimization indicated 19 features as being most important for separation of both populations using a Euclidean distance matrix. A classifier algorithm was used to compile these 19 metrics for each given class sample in each population and then used to classify all remaining cells in that tissue. A selection of at least 10 incorrectly classed cells were then manually corrected and added to the relevant class sample populations before recompiling the 19-dimensional feature space and reclassifying the whole tissue. This iterative learning process was repeated at least 10 times with a final sample size of 400-500 cells for each class. The classifier was stored as .xml and used to batch classify the entire data set of tissues from all patients.

Multiplex Immunohistochemistry

Chromogenic sequential IHC was conducted with 5 μm of FFPE tissue sections. Following de-paraffinization, slides were stained by hematoxylin (S3301, Dako) for 1 min, followed by whole tissue scanning using Aperio ImageScope AT (Leica Biosystems). Slides were subjected to endogenous peroxidase blocking followed by heat-mediated antigen retrieval. Primary antibodies were detected using a species-specific F(ab') fragment-specific secondary antibody-labeled polymer-based peroxidase system (Histofine, Nichirei Biosciences Inc, Japan) in conjunction with 3-amino-9-ethylcarbazole (AEC). Digital scanning of antibody/chromogen staining, and stripping performed as described (Tsujikawa et al., 2017). Hematoxylin was used to identify cell nuclei. Regions of interest (ROIs) (three regions/slide), encompassing the total tissue area for quantitation were selected using ImageScope (Leica). Images were co-registered (aligned) in MatLab utilizing a sift/surf algorithm. Color deconvolution then performed to extract signal for quantitation of signal intensity and attribution of signal to single cells (based on masks made in previous steps) performed in cell profiler. Image cytometry gating then performed in FCS Express 5 Image Cytometry (De Novo Software) and cell populations determined using multiparameter cytometric image analysis. Cell populations were normalized to total cell number (cells/total cells) and populations were quantified. Unsupervised hierarchical clustering was performed using R package pheatmap_1.0.8. Correlation was used as a distance measure and average was used as clustering method (Tsujikawa et al., 2017).

Target	Company/Product#	Clone	Dilution
CD45	eBiosciences: 14-0459-82	H130	1:100
CD3	ThermoFisher Scientific:MA1-90582	SP7	1:150
CD8	ThermoFisher Scientific:MA5-13473	C8/144B	1:100
CSF1R	abcam: ab183316	SP211	1:150
CD169	Millipore: MABT328	5F1.1	1:200
CD163	ThermoFisher: MA5-11458	10D6	1:100
CD56	Santa Cruz Biotech	123C3	1:100
CCR2	Novus: MAB150-SP	48607	1:400

Antibodies Used for Multiplex IHC

CCL8 *mRNA, SIGLEC1 and CD163 Protein Detection in Human Breast Cancer Biopsies*

Mixed multiplex staining (RNAscope, Tyramide dual immunofluorescence) for CCL8 mRNA, CD169 (SIGLEC1) and CD163 protein detection was performed on a Leica RX research-staining robot (Newcastle, UK). RNAscope (ACD Bio Newark, CA) was performed using manufacturers recommendations using ACD LS2.5 Brown kit (322100) as follows. FFPE fixed breast cancer needle biopsies were dewaxed in xylene and rehydrated through graded ethanol, following a brief rinse in water sections were washed in tris buffered saline containing 0.01% v/v tween 20 (TBST). Slides were placed onto a Leica RX robot and stained using the manufacturer's recommended LS2.5 Brown RNAscope protocol. mRNA integrity was assessed using PPIB (cat 313908) using the following standard tissue pretreatments: ACD ER2 for 10 min with ACD Protease 5 min or ACD ER2 at 95°C for 15 min with ACD Protease 15 min or ACD ER2 at 95°C for 20 min with ACD Protease 25 min. Mild conditions (ACD ER2 95°C 10 min) with ACD protease (5 min) were assessed as providing optimal mRNA detection whilst maintaining both protein antigenicity and tissue section morphology of these relatively delicate sections. Following tissue pretreatment the standard recommended protocol was followed, briefly AMP1 30 min, AMP2 15 min, AMP3 30 min, AMP4 15 min, AMP5 30 min and AMP6 15 min followed by visualisation in DAB for 20 min using standard recommended washes. Modification of standard protocol for CCL8 (466498) to obtain a fluorescent endpoint involved omitting DAB substrate and replacing with Tyramide Cy5 at 1:100 dilution (Perkin Elmer, NEL745001KT, Seer Green, UK).

Following CCL8 mRNA detection using RNAscope with Cy5, sections were sequentially stained for CD169 protein with Tyramide Cy3 detection and CD163 with Tyramide FITC detection using heat elution between detections for specificity. Using a Leica RX robot slides were subject to further Heat Induced Epitope Retrieval (HIER) using ER1 retrieval buffer for 10 min at 99C, followed by blocking in 3% Hydrogen Peroxide and 20% Normal Goat Serum. CD169 (Novus Biologicals, NBP2-30903, Cambridge, UK) was added to sections at 1:100 dilution for 60 min followed by secondary antibody Goat anti Rabbit Peroxidase fab at 1:500 dilution (Abcam, ab7171, Cambridge, UK) before visualisation with Tyramide Cy3 at 1:50 dilution. (Perkin Elmer, NEL744E001KT). Stripping of antibodies from the tissue sections was performed for 10 min at 99C followed by blocking in 3% v/v Hydrogen Peroxide and 20% v/v Normal Goat Serum. CD163 (Leica Biosystems NCL-LCD163, Clone 10D6) was added to sections at 1:1000 dilution for 60 min followed by secondary antibody Goat anti Rabbit Peroxidase fab at 1:500 dilution (Abcam, ab7171) before visualisation with Tyramide FITC (Perkin Elmer, NEL741001KT) at 1:50 dilution and counterstaining with DAPI at 1:1000 dilution. All washes between incubations were for 2 x 5 min in TBST (Tóth and Mezey, 2007).

ELISAs

Human CCL8, TNFα, and IL1β protein levels were quantified by Duoset ELISA kits (R&D systems, USA) following manufacturer's instructions. Human CSF1 and CX3CL1 protein levels were quantified by quantikine ELISA kit (R&D systems, USA). CCL2 levels were assessed in plasma from 15 healthy donors and 42 breast cancer patients using Legendplex bead-based immunoassays (Biolegend) according to manufacturer's protocol. Data were collected using the C4 Accuri (BD). All cell culture supernatants were used undiluted.

Cytokine Array

Human Cytokine ELISA Plate Array (Signosis, EA-4002), consisting of one pre-coated plate able to detect 32 cytokines simultaneously for 3 independent human samples was used to quantify cytokines in supernatants from MDMs before or after cancer CM stimulation. Detection of cytokines produced from MDMs before or after CM stimulation was performed based on the manufacturers instructions. 8.0 μl of MDM supernatants from each group was added into each well of the plate and incubated at room temperature for 2 hr. After washing, 100μl of diluted biotin-labelled antibody mixtures were added into each well for another one-hour incubation. After washing again, each well was incubated with detection antibody mix and then HRP, and the plate was read on a plate reader at 450 nm. The full list of proteins detected and raw data can be found in Table S4.

iPSDM-Cancer Cell Conditioned Medium Production

Human iPSDM culture medium was replaced with 10% v/v FBS DMEM 24h before CM incubation. iPSDM were incubated with MDA-MB-231 and -468 CMs (prepared as described above) for 24 hr and then medium was changed; after medium change, cells were cultured for an additional 24 hr with fresh medium and thereafter, cell free supernatants were harvested and directly used for the experiment.

TNFα Neutralization in Conditioned Medium

iPSDM-Cancer cell conditioned medium was incubated for 24 hr with 1.0 μg/ml of mouse anti human TNFα neutralizing antibody (R and D systems, USA, MAB210-SP, Clone 1825) or 1.0 μg/ml of mouse IgG_1 isotype control (R and D systems, USA, MAB002). Efficacy of anti-TNFα antibody neutralization was tested by TNFα ELISA before use.

PCR Arrays

PCR-based microarrays for evaluating the expression of genes mediating the inflammatory response were performed using the human inflammatory cytokines and receptors RT2 Profiler TM PCR array (Qiagen, PAHS-011ZE-4); PCR-based microarrays for evaluating the expression of genes in breast cancer cell lines were performed using the Breast cancer PCR array RT2 Profiler TM PCR array (Qiagen, PAHS-131Z-4) and the Tumor Metastasis PCR array RT2 Profiler TM PCR array (Qiagen, PAHS-028Z). The arrays

were configured in a 384-well plate consisted of a panel of 92 genes and 4 endogenous genes. Reverse transcription was performed using the RTC First Strand Kit (Qiagen, 330401) and qPCR was performed using RTC SYBR Green/ROX PCR Master mix (Qiagen, 330521), and the raw data were analyzed by the GeneGlobe Data Analysis Center (www.qiagen.com) according to the manufacturer's instructions.

Cell Proliferation Assay

Cell proliferation was determined using the Cell Counting Kit (CCK)-8 assay (Sigma-Aldrich, 96992) according to the manufacturer's instructions. A total of 5,000 cells were seeded into each well in the 96-well plates and allowed to attach overnight. Cells were then treated with 0.1 ng/ml, 1.0 ng/ml or 10 ng/ml CCL8 (R&D Systems). After the treatment (6 to 72 hr), a CCK-8 solution was added to each well and then cells were incubated at 37°C for 2 hr. Cell proliferation was measured using the microplate reader and the proliferation of cells was defined as OD450-OD620.

In Vitro Cell Migration Assay

Cells were grown in DMEM with 10% v/v FBS in 12-well plates until they reached confluence; after 24 hr of starvation (DMEM 0% FBS), a scratch was performed using a p200 tip. Recombinant CCL8 and CCL2 (R & D systems) were used at 1ng/ml concentrations in all the experiments. Cells were filmed for 24 hr in a 37°C thermostatic chamber using an Axiovert Scope. 2-3 independent sections/well were filmed and 4 independent experiments per condition were performed. Data analysis was performed with Image J (NIH) (Liang et al., 2007).

Chemotaxis Assay

THP1 chemotaxis was performed using Essen Biosciences reagents. THP1 cells were cultivated in RPMI medium with 10% v/v FBS and seeded at 4000 cells/well in 96 well chemotaxis plates (Essen Biosciences) in the presence or absence of 20 ng/ml recombinant human CCL2 or CCL8 (R & D systems). Migration was recorded every hour for 72 hr using the IncuCyte system (Essen Bioscience) and number of cells migrated was calculated using IncuCyte quantification software.

QUANTIFICATION AND STATISTICAL ANALYSIS

Sequencing Alignment and Quantification

FastQ files of 2x100bp paired-end reads were quality checked using FastQC (Andrews, 2012). Samples were filtered for low quality reads (Phred score >= 20) and adapters were removed when necessary using Cutadapt. Quality controlled reads were then aligned to the Human reference genome (GRCh37/hg19) using STAR aligner (version 2.3) (Dobin et al., 2012). Quantification of genes was performed using the count function of HTSeq (Anders et al., 2014). Reads were counted at the gene level and the unstranded option was used (-s no).

Statistical Analysis for RNA-seq Data

All statistical calculations were performed in R programming language (version 3.2.3). For macrophage samples, genes with count per million (CPM) reads > 1 in at least N samples (N number of the fewest replicates of a condition) was retained. Gene expression levels were normalized using the Trimmed Mean of M-values (TMM) method using the calcNormFactors() function and $\log_2$ transformed using the cpm() function from the EdgeR package in R (Robinson and Oshlack, 2010). Differential expression analysis was performed with sample quality weights using the package limma-voom package in R (Ritchie et al., 2015). For monocyte samples, gene expression levels were normalized according to upper-quantile normalization using calcNormFactors() function and $\log_2$ transformed using the cpm() function. Normalized reads were fed to the Combat function of the Surrogate Variable Analysis (SVA) package (Leek, 2014). Samples were corrected on the basis of the sequencing batch they were processed. Differential expression analysis was performed using the limma package. Significantly differentially expressed genes (DEGs) were selected with controlled False Positive Rate (B&H method) at 5% (FDR <= 0.05). Upregulated genes were selected at a minimum $\log_2$ fold change of 1.5 and down-regulated genes at a minimum $\log_2$ fold change of -1.5. PCA plots were drawn using the TMM/$\log_2$ transformed (macrophages) or the batch effect corrected (monocytes) normalized values on expressed genes. Heatmaps were drawn on the normalized expression matrix using the pheatmap package in R. Euclidean distance and complete linkage were used for hierarchical clustering. Venn diagrams were constructed based on the overlapping differentially expressed transcripts (FDR <= 0.05, Log_2FC more or less than 1.5/-1.5).

Enrichment and Pathway Analysis

Gene set enrichment analysis was performed using the gsea() function from phenoTest package in R (Planet, 2013). The function is used to compute the enrichment scores and simulated enrichment scores for each variable and signature. For our analysis, the logscale variable was set to false, as the $\log_2$ transformed expression values were fed into the function and 20,000 simulations were used (B = 20,000). The Database for Annotation, Visualization and Integrated Discovery (DAVID) functional annotation tool was used for gene ontology and pathway (KEGG and Reactome) analysis on the list of differentially expressed genes (FDR <= 0.05, Log_2FC more or less than 1.5/-1.5). Important GO terms and pathways were selected based on an FDR <= 0.05.

Recursive Feature Elimination with Random Forest

Recursive feature elimination (RFE) is a wrapper feature selection method that starts by fitting the classification model to all features and then each feature is ranked for its importance to the model. We assume that S is a sequence of values that indicates the number of features to be retained. At each iteration, the S_i top ranked features are used to refit the model and the subset of genes with the highest accuracy is selected. We used the RFE feature selection with resampling and a Random forest (RF) classifier as implemented in the caret package in Bioconductor (Kuhn, 2015). In brief, RF is an ensemble learning method that constructs many decision trees (forest) and uses a majority vote to make predictions (Breiman, 2001). Assuming N number of samples in the training set, the algorithm creates a subset of the data of the N samples with replacement, and at each node, *m* number of genes are selected at random from all the genes M. The variable *m* that gives the best split is selected and used for a binary split. This procedure is repeated for each node until the tree is grown to terminal nodes k.

Before model training and feature selection, the breast TEMo dataset of n = 77 samples (32 healthy, 45 breast cancer) was filtered for lowly expressed genes (CPM > 1 across conditions), normalized using upper-quantile normalization, log_2 transformed using the *cpm()* function and corrected for batch effects using ComBat. Then, the dataset was split into training (70%, n = 55, 32 healthy, 23 cancer samples) and testing (30%, n = 22, 13 healthy, 9 cancer samples). While the test set was kept on the side, the training set was used for RFE-RF model training and feature selection using 5 times repeated 10-fold cross-validation (CV). In short, the training samples were randomly partitioned into k (k = 10) subsamples. Out of the k sub-samples, one is kept for testing the classifier, and the remaining k-1 subsamples are used for training the classifier. Then the whole process is repeated n= 5 times. For the training set, overall accuracy, sensitivity and specificity were calculated averaging the CV predictions for the optimal subset. The model with the highest average accuracy was selected as optimal. The optimal model was the fitted on the test set that had not been used for training or feature selection. For the RFE-RF model, we selected subsets of features ranging between 2 to 30, variable *ntree* was set to 500 trees and variable *mtry* was calculated as $\sqrt{p}$, where p is the number of features used during training the model. The receiver-operating characteristic (ROC) curves were drawn using the ROCR package in R (Sing et al., 2005). To determine the accuracy rates of the classifiers that can be obtained by chance, a RF model using the 17 selected genes was trained with permuted class labels. This process was performed during training 1,000 times using 5 x 10-fold CV. The accuracy of the 1,000 random classifiers was recorded. The p value was calculated by counting the accuracy of the random classifiers that achieved similar or higher total accuracy compared to the observed accuracy of the RFE-RF model on the training data.

Publically Available Datasets

The following publically available datasets were used in this study:

a) Karnoub et al. (GSE8977) (Karnoub et al., 2007): total of 22 samples coming from breast ductal carcinoma-in-situ (DCIS) patients (n = 15) and invasive ductal (IDC) breast cancer patients (n = 7) were downloaded from GEO. Samples we processed and normalized using the robust Multi-Array average expression measure (RMA) from the affy package in R. Probes representing the same gene were averaged to a single value.
b) Finak et al. (GSE9014) (Finak et al., 2008): total of 59 samples coming from breast cancer stroma patients (n = 53) and healthy controls (n = 6) including updated clinical information were downloaded from GEO. Technical replicates were averaged to a single array using the averarrays() function from limma package in R. Data were then quantile normalized using the normalizeQuantiles() function. Samples were annotated and probes representing the same gene were averaged to a single value.
c) METABRIC cohort (Curtis et al., 2012): Microarray gene expression data and associated clinical information (n = 1980) (Log_2 transformed intensity values) were downloaded from the cBioPortal for cancer genomics database (http://www.cbioportal.org/) under the study name Breast cancer. Gene expression values were quantile normalized and samples with gene expression and corresponding clinical information were selected resulting in n = 1353 patients. Data were filtered for missing values and samples with molecular subtype NC were removed. The filtering resulted in n = 1350 patients that were used for further analysis. For survival analysis, events were censored based on disease-related deaths (Died of disease = 1; Living or Died of other causes = 0).
d) Cancer cell Encyclopedia (CCLE) data: Gene expression RPKM normalized reads from breast cancer cell lines (n = 57) were downloaded from the CCLE website (https://portals.broadinstitute.org/ccle).

TAM Signature

As a starting point for the selection of the immune signature we used the upregulated genes in Br-TAM compared to Br-RM (n = 553, $Log_2FC > 3$ and FDR < 0.05). This gene list of highly differentially expressed genes was filtered using the compendium of immune genes that includes 17 immune cell-specific gene sets as initially assembled by Bindea et al. and Charoentong et al. (Bindea et al., 2013; Charoentong et al., 2017) and most recently validated by Tamborero et al. (Tamborero et al., 2018) (Table S3). After filtering, n = 528 TAM related genes were selected. In order to identify the most relevant genes we used the METABRIC cohort and correlation analysis. Genes were considered coexpressed when having an absolute Pearson correlation of R >= 0.5 (findCorrelation() function from the caret package in R (Kuhn, 2015). This threshold was selected in order to satisfy two main aims: a) genes with relatively high correlation would not be considered a chance event; b) selection of a relatively small number of genes in order to be suitable for gene set enrichment and survival analysis (Charoentong et al., 2017). Additionally, genes were

selected based on their positive Pearson correlation ($R > 0.5$, $p <= 0.05$) with known TAM marker CD163, resulting in n = 37 genes (Table S3). Finally, we downloaded breast cancer cell line data from the CCLE database (n = 57 breast cancer cell lines) in order to filter out genes expressed by tumor cells. Genes with median expression of Log_2RPKM > 6 were considered expressed in tumor cells (TAM: median = 0.031). This resulted in a set of 37 genes expressed by TAMs and not tumor cells or other immune-specific signatures (Table S3). The TAM and macrophage signature scores were calculated as the median of expression of the TAM or macrophage signature genes (Tamborero et al., 2018) using the median centered normalized values.

Survival Analysis

For the TAM signature and SIGLEC1/CCL8 signature the summed normalized gene expression values were dichotomized based on the optimal cutoff calculated by iteratively calculating every possible expression cutoff (n-1) and selecting the value with the lowest p value (Pearce et al., 2017). For the METABRIC cohort, disease-specific survival (DSS) was used as an endpoint. For the breast cancer stroma dataset, recurrence-free survival (RFS) was used as an endpoint and censored at date of last follow-up. Survival curves were estimated using the Kaplan Meier method (survival and survminer R packages). For SIGLEC1 and CCL8 single gene survival analysis, clinical risk factors such as ER status (+/-), PR status (+/-), Her2 status (+/-), histological grade (I, II or III), age (greater or less 55) and tumor size (greater or less than 50mm) were used in the univariate and multivariate models. Candidate prognostic factors for RFS and DSS with a p value (Wald test) lower than 0.05 in univariate analysis were used in the multivariate analysis. Multivariate analysis was performed by fitting a Cox proportional hazard regression model. The Cox regression model was used to calculate the Hazard ratio (HR) and 95% confidence internal (CI). A p value less than 0.05 based on a Wald test was considered significant.

RNA-seq of Total Tissue Breast Cancer

RNA isolated from 47 breast cancer tumors (cohort 3) was utilized for RNA-seq. These RNA samples were converted into a library of cDNA fragments. Illumina sequencing adapters were added and 50 bp single end read sequence was obtained using Illumina HiSeq. Quality check was performed on these sequence reads using FastQC. PCR primers and adapters were filtered out of the sequence reads using Trimmomatic (Bolger et al., 2014). Filtered reads were aligned to reference genome build hg19 using TopHat 2.0.12, a splice junction aligner (Kim et al., 2013). Aligned sequences were assembled into transcripts. Transcript abundance was estimated as Fragments Per Kilobase of exon per Million fragments mapped (FPKM), using Cufflinks 2.2.1 (Roberts et al., 2011; Trapnell et al., 2010). FPKM estimates were normalized using Cuffnorm. Further data was quartile normalized and batch effects were removed using ComBat (Johnson et al., 2007). Samples were classified into CSF1 High, Mid and Low expression groups using K-means clustering on the expression of CSF1 signature genes (Beck et al., 2009). TAM signature score was estimated from the median of expression of TAM signature genes. Samples were assigned to breast cancer subtypes based on hierarchical clustering of PAM50 genes (Parker et al., 2009). Clustering was performed using R package pheatmap_1.0.8. Correlation was used as a distance measure and average was used as clustering method.

Statistical Analysis

Statistical significance was calculated by Student's t-test when comparing two groups or by one-way or two-way ANOVA when comparing three or more groups. A p value < 0.05 was considered as statistically significant.

DATA AND SOFTWARE AVAILABILITY

The accession numbers for the RNA seq data reported in this paper are GSE100925 and GSE117970.

Cancer Cell, Volume *35*

Supplemental Information

Human Tumor-Associated Macrophage and Monocyte Transcriptional Landscapes Reveal Cancer-Specific Reprogramming, Biomarkers, and Therapeutic Targets

Luca Cassetta, Stamatina Fragkogianni, Andrew H. Sims, Agnieszka Swierczak, Lesley M. Forrester, Hui Zhang, Daniel Y.H. Soong, Tiziana Cotechini, Pavana Anur, Elaine Y. Lin, Antonella Fidanza, Martha Lopez-Yrigoyen, Michael R. Millar, Alexandra Urman, Zhichao Ai, Paul T. Spellman, E. Shelley Hwang, J. Michael Dixon, Lisa Wiechmann, Lisa M. Coussens, Harriet O. Smith, and Jeffrey W. Pollard

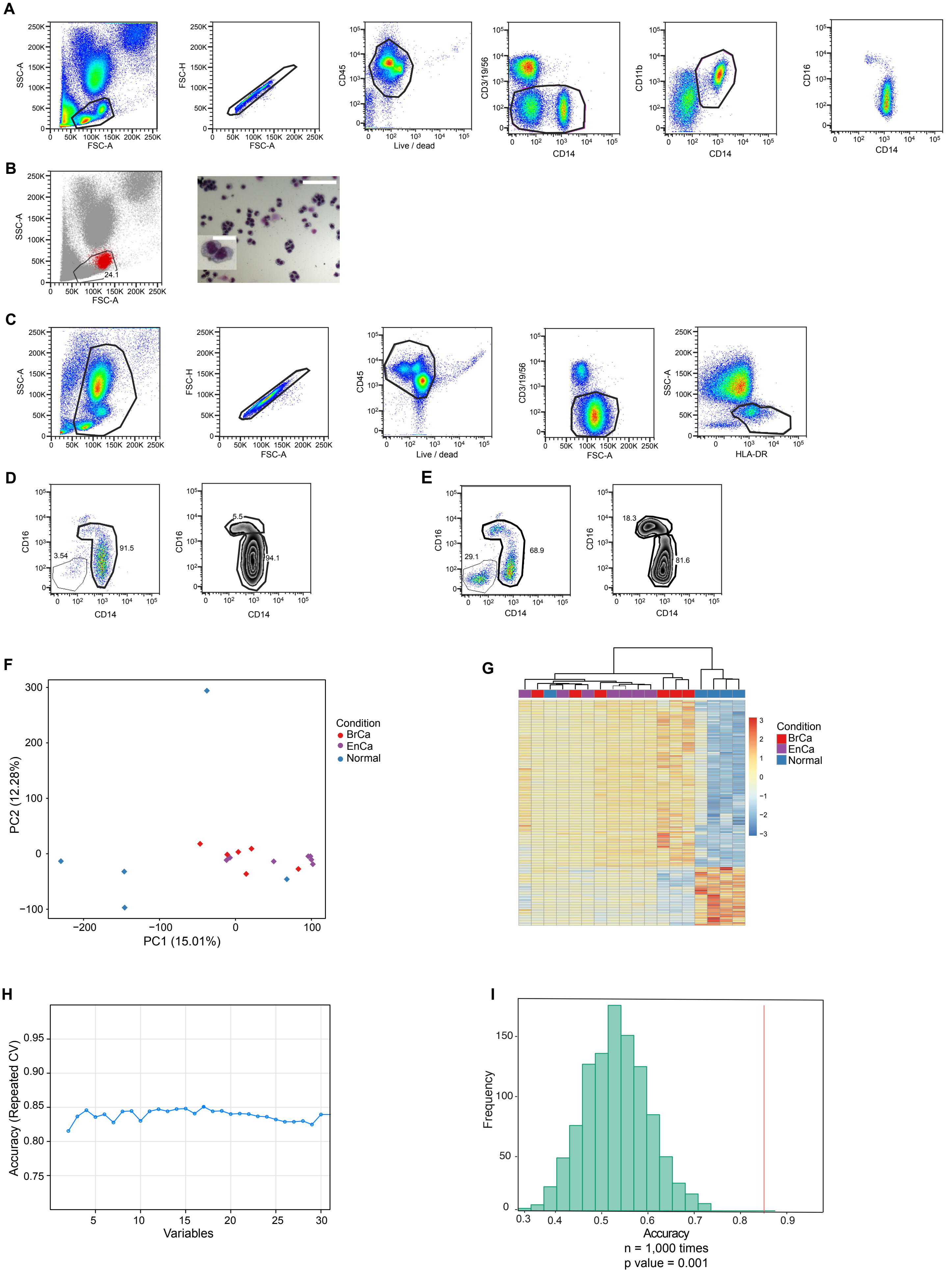
A
SSC-A
FSC-A
FSC-H
CD45
Live / dead
CD3/19/56
CD14
CD11b
CD16
B
24.1
C
HLA-DR
D
3.54
91.5
5.5
94.1
E
29.1
68.9
18.3
81.6
F
PC2 (12.28%)
PC1 (15.01%)
Condition
BrCa
EnCa
Normal
G
Condition
BrCa
EnCa
Normal
H
Accuracy (Repeated CV)
Variables
I
Frequency
Accuracy
n = 1,000 times
p value = 0.001

Figure S1. Flow cytometry gating strategy for the identification and isolation of human monocytes and transcriptomic analysis of the non-classical sub-population, related to Figure 1.

(A) Representative monocyte gating strategy based on physical and fluorescence parameters.

(B) Validation of gating strategy was performed by backgating and nuclei coloration (Giemsa, staining, scale bar 50 µm, inset 10 µm). Representantive monocytic cell shown.

(C, D and E) Representative monocyte gating strategy based on physical, and fluorescence parameters (C) of classical and non-classical monocytes separation in healthy controls (D) and cancer patients (E).

(F) PCA plot of n = 12,712 genes expressed in non-classical monocytes derived from healthy individuals (n = 6), breast cancer (BrCa) (n = 6) and endometrial cancer (EnCa) patients (n = 7).

(G) Hierarchical clustering on all samples (Healthy, n = 5; BrCa, n = 6; EnCa, n = 7) using the significantly DEGs between breast and healthy non-classical monocytes (n = 139 genes). Expression values are Z score-transformed and samples clustered using complete linkage and Euclidean distance.

(H) Plot of the accuracy yielded for different gene signature sizes during feature selection with Recursive feature elimination with Random forest (RFE-RF) model training.

(I) Histogram showing the performance of random classifiers during Random forest (RF) model training. Solid vertical red line represents the performance of the observed 17-gene signature on the training data.

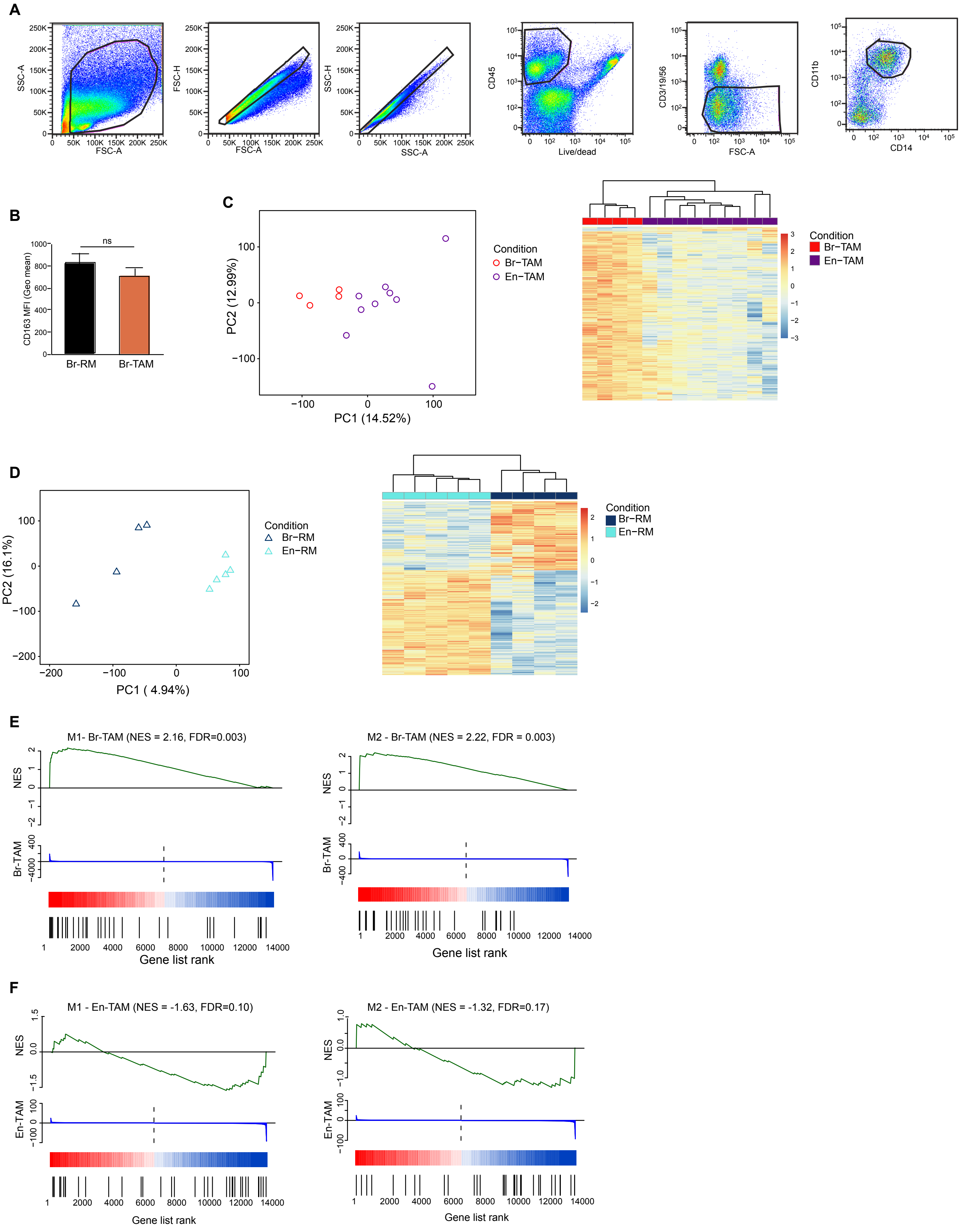
A
SSC-A
FSC-A
FSC-H
FSC-A
SSC-H
SSC-A
CD45
Live/dead
CD3/19/56
FSC-A
CD11b
CD14
B
ns
CD163 MFI (Geo mean)
Br-RM
Br-TAM
C
PC2 (12.99%)
PC1 (14.52%)
Condition
Br−TAM
En−TAM
D
PC2 (16.1%)
PC1 (4.94%)
Condition
Br−RM
En−RM
E
M1- Br-TAM (NES = 2.16, FDR=0.003)
M2 - Br-TAM (NES = 2.22, FDR = 0.003)
NES
Br-TAM
Gene list rank
F
M1 - En-TAM (NES = -1.63, FDR=0.10)
M2 - En-TAM (NES = -1.32, FDR=0.17)
NES
En-TAM
Gene list rank

Figure S2. Flow cytometry sorting strategy of tissue macrophages and TAMs, and analysis of CD163 expression and distinct macrophage populations, related to Figure 2.

(A) Gating strategy for tissue macrophages and TAMs; macrophages were defined as $CD45^{+}CD3/56/19^{-}CD11b^{+}CD14^{+}CD163^{+}$.

(B) Representative histogram of macrophage CD163 expression in Br-MR (n = 5) compared to Br-TAM (n = 5). Data are expressed as Geometric Mean (Mean±SEM).

(C) PCA plot of n = 14,229 expressed genes in Br-TAM (n = 4) and En-TAM (n = 9) (left). Hierarchical clustering of all DEGs between Br-TAM and En-TAM (right). Expression values are Z score-transformed and samples clustered using complete linkage and Euclidean distance.

(D) PCA plot of n = 13,907 expressed genes in Br-RM (n = 4) and En-RM (n = 5) (left). Hierarchical clustering of all DEGs between Br-RM against En-RM (right).Expression values are Z score-transformed and samples clustered using complete linkage and Euclidean distance.

(E) Enrichment analysis of M1-like (left) and M2-like (right) macrophage signature (Martinez et al., 2006) in Br-TAM. Black bars represent the position of M1-like or M2-like genes in the ranked list of Br-TAM expressed genes together with the running enrichment score (green line).

(F) Enrichment analysis of M1-like (left) and M2-like (right) macrophage signature (Martinez et al., 2006) in En-TAM. Black bars represent the position of M1-like or M2-like genes in the ranked list of En-TAM expressed genes together with the running enrichment score (green line).

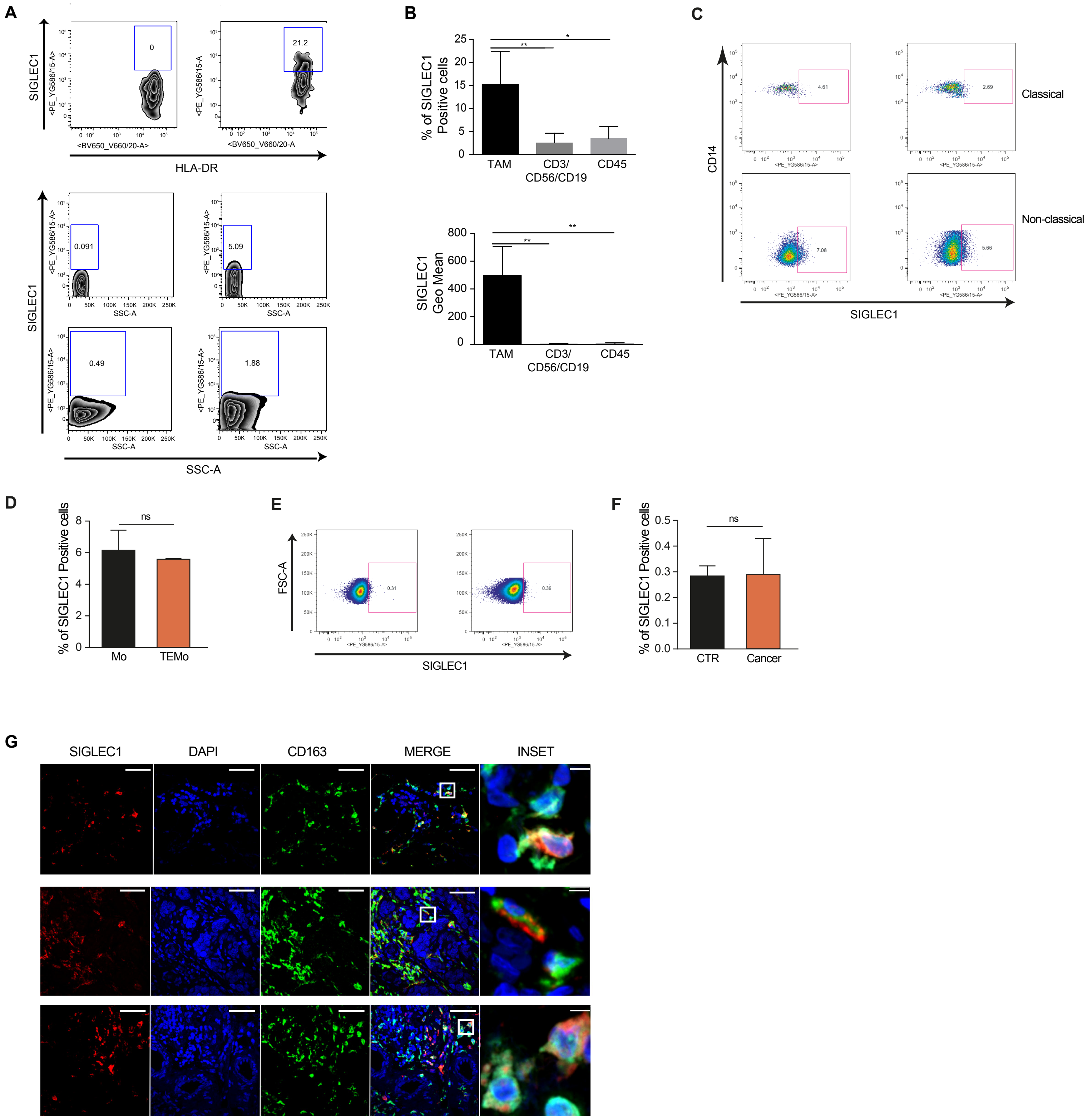
A
SIGLEC1
HLA-DR
0
21.2
0.091
5.09
0.49
1.88
SSC-A
B
% of SIGLEC1 Positive cells
SIGLEC1 Geo Mean
TAM
CD3/ CD56/CD19
CD45
C
CD14
SIGLEC1
Classical
Non-classical
4.61
2.69
7.08
5.66
D
% of SIGLEC1 Positive cells
ns
Mo
TEMo
E
FSC-A
SIGLEC1
0.31
0.39
F
% of SIGLEC1 Positive cells
ns
CTR
Cancer
G
SIGLEC1
DAPI
CD163
MERGE
INSET

Figure S3. Expression of SIGLEC1 in Br-TAM and cell types, related to Figure 4.

(A) SIGLEC1 expression in Br-TAM samples (top), in $CD3^+/19^+/56^+$ cells (middle) and $CD45^-$ cells (bottom) from breast tumors. FMO controls (left), SIGLEC1 staining (right). Representative plot of 3 independent experiments is shown.

(B) Quantification of $SIGLEC1^+$ cells in Br-TAM, $CD3^+/19^+/56^+$ cells and $CD45^-$ cells from breast tumors ($n = 3$). Data are depicted as % of $SIGLEC1^+$ cells (top) and Geo Mean values (bottom).

(C) SIGLEC1 expression in classical and non-classical Mo and breast cancer TEMo (square gate based on FMO control). Representative plot of 3 independent experiments is shown.

(D) Quantification of % of $SIGLEC1^+$ cells in Mo and TEMo ($n = 3$). Data are depicted as % of SIGLEC1+ cells.

(E) SIGLEC1 expression in blood circulating granulocytes from healthy donors (CTR) and breast cancer patients (gated on live $CD45^+$ SSC high cells, square gate based on FMO control). Representative plot of 3 independent experiments is shown.

(F) Quantification of % of $SIGLEC1^+$ cells in blood circulating granulocytes from healthy donors (CTR) and breast cancer patients ($n = 3$).

(G) CD163 and SIGLEC1 immunofluorescent staining on breast cancer tissue samples ($n = 5$ each, Bars = 50 μm, inset = 5 μm, 3 representative samples are shown). Enlargement of the selected area showing a representative $SIGLEC1^+CD163^+$ macrophage.

ns = not significant, $*p < 0.01$, $**p < 0.001$; (B) One-way ANOVA, (D,F) Student’s t-test, (B, D ,F) Data depicted as Mean±SEM.

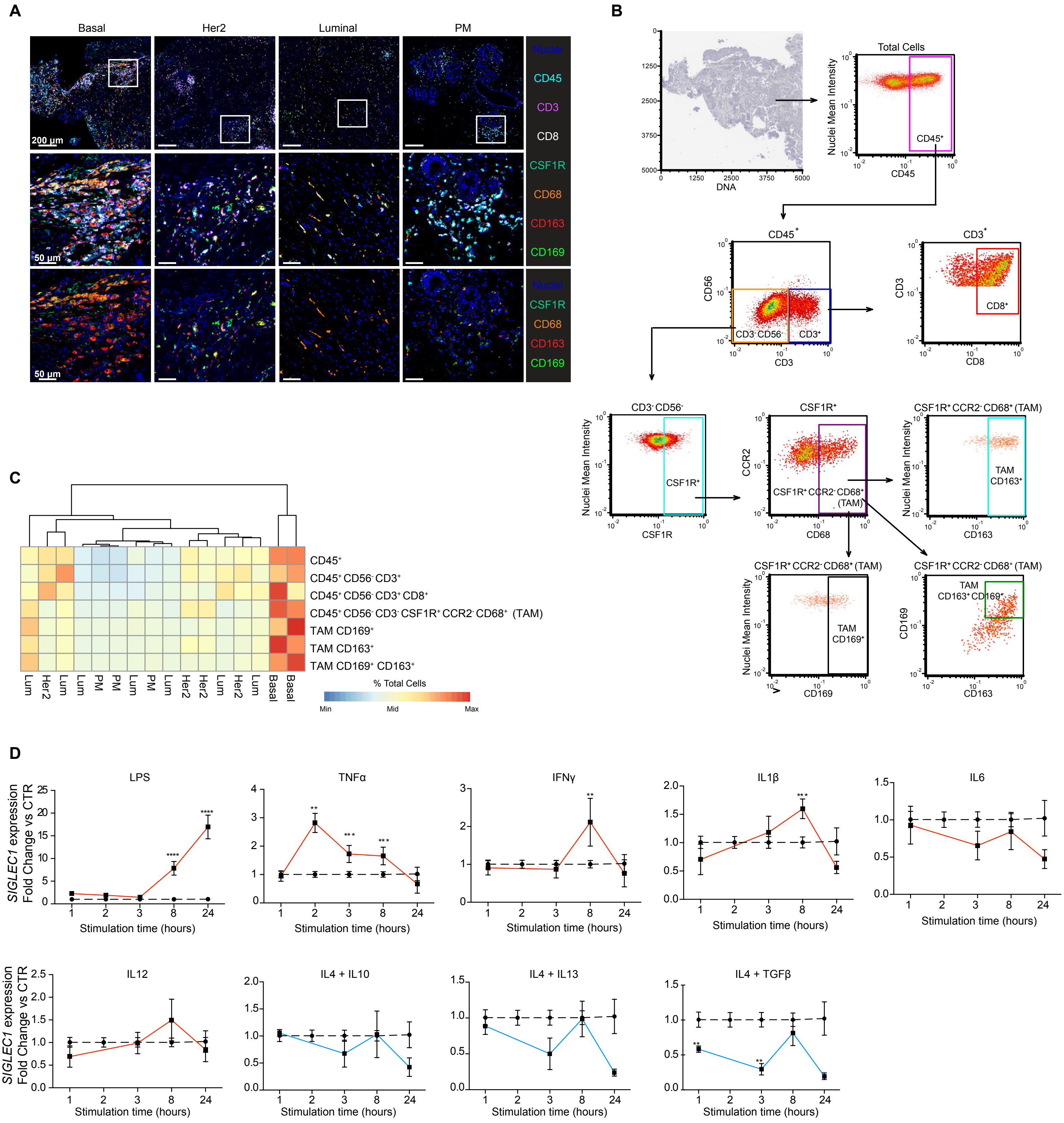
A
Basal
Her2
Luminal
PM
200 µm
50 µm
Nuclei
CD45
CD3
CD8
CSF1R
CD68
CD163
CD169
B
Total Cells
CD45+
CD3+
CD8+
CD3- CD56-
CSF1R+
CSF1R+ CCR2- CD68+ (TAM)
TAM CD163+
TAM CD169+
TAM CD163+ CD169+
Nuclei Mean Intensity
DNA
CD56
CCR2
C
CD45+
CD45+ CD56- CD3+
CD45+ CD56- CD3+ CD8+
CD45+ CD56- CD3- CSF1R+ CCR2- CD68+ (TAM)
TAM CD169+
TAM CD163+
TAM CD169+ CD163+
Lum
Her2
PM
Basal
% Total Cells
Min
Mid
Max
D
LPS
TNFα
IFNγ
IL1β
IL6
IL12
IL4 + IL10
IL4 + IL13
IL4 + TGFβ
SIGLEC1 expression Fold Change vs CTR
Stimulation time (hours)

Figure S4. Multiplex immunohistochemistry analysis of different breast cancer subtypes and SIGLEC1 regulation by cytokines, related to Figure 4.

(A) Representative micrographs reflecting pseudo-colored images following multiplex IHC of cell populations across breast cancer subtypes as indicated. Boxed insets are depicted at higher magnification in corresponding columns. Scale bars as indicated (n = 16).

(B) Image cytometry plots of quantitative multiplex immunohistochemistry on tissue biopsies. Cumulative cell populations from total tissue areas were normalized to total cell number. One representative sample out of 16 is shown.

(C) A heatmap of each cell population as a percent of total cells is shown with a dendrogram of unsupervised hierarchical clustering, scaled by row and using correlation as a distance measure, and average as a clustering method. Each column represents an independent tumor according to sub-type. (Lum: luminal) and prophylactic mastectomy/mammoplasty (PM) samples, (n = 16).

(D) *SIGLEC1* mRNA expression in PMA-treated THP1 cells stimulated with pro- (red) or anti-inflammatory (blue) cytokines as indicated. LPS acts as a positive pro-inflammatory signal control. Colored bar indicates *SIGLEC1* expression in cytokine-treated samples, dotted black line indicates *SIGLEC1* expression in control PBS-treated samples. Data are depicted as fold change vs CTR (n = 3, **p < 0.001, ***p<0.0001, ****p<0.00001; Student's t-test, Mean ± SEM).

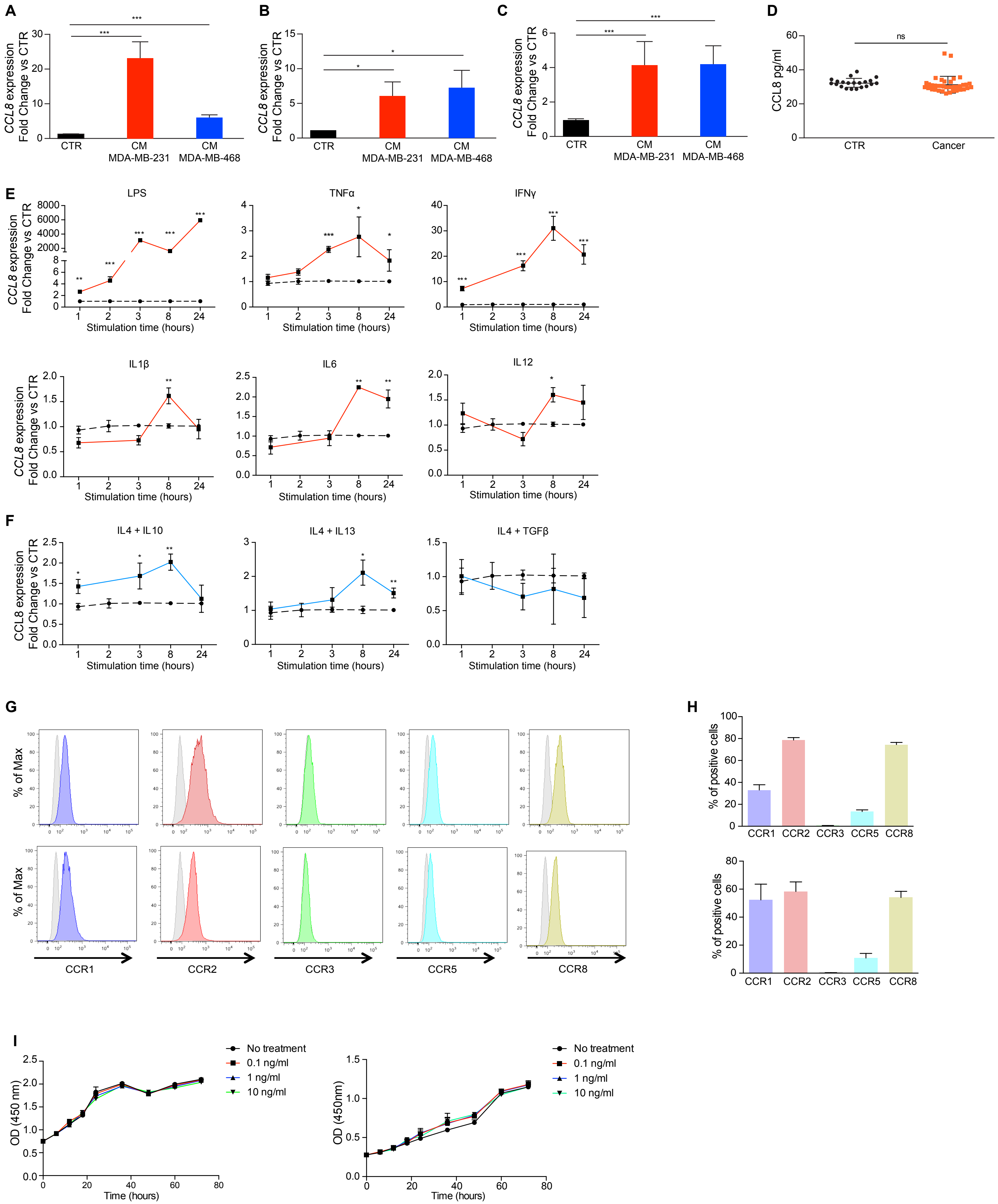

A
B
C
D
CCL8 expression Fold Change vs CTR
CTR
CM MDA-MB-231
CM MDA-MB-468
CCL8 pg/ml
Cancer
ns
E
LPS
TNFα
IFNγ
IL1β
IL6
IL12
Stimulation time (hours)
F
IL4 + IL10
IL4 + IL13
IL4 + TGFβ
G
% of Max
CCR1
CCR2
CCR3
CCR5
CCR8
H
% of positive cells
I
No treatment
0.1 ng/ml
1 ng/ml
10 ng/ml
OD (450 nm)
OD (450nm)
Time (hours)

Figure S5. Expression of SIGLEC1 and CCL8 in macrophages after stimulation with cancer cell conditioned medium or cytokines, related to Figure 5.

(A, B and C) *CCL8* mRNA expression in PMA-treated THP1 cells (A), primary MDM (B), and iPSDM (C) stimulated for 24 hr with culture medium (CTR), MDA-MB-231 CM or MDA-MB-468 CM. Data are depicted as fold change vs CTR ($n = 3$).

(D) ELISA for CCL8 in serum of healthy donors ($n = 21$) and breast cancer patients ($n = 38$). Data are depicted as pg/ml.

(E and F) *CCL8* mRNA expression in PMA-treated THP1 cells stimulated for times shown with pro- (E) or anti-inflammatory (F) cytokines as shown. Colored line indicated cytokine-treated samples, dotted black line PBS-treated samples; Data are depicted as fold change vs CTR ($n = 3$).

(G) Representative histograms of CCR1, CCR2, CCR3, CCR5 and CCR8 expression in MDA-MB-231 (top) and MDA-MB-468 (bottom) cells. Grey histograms indicate unstained samples. Representative plot of 3 independent experiments is shown.

(H) Percentage of CCR1, CCR2, , CCR3, CCR5 and CCR8 positive cells in total MDA-MB-231 (top) and MDA-MB-468 cells (bottom), ($n = 3$).

(I) MDA-MB-231 (left) and MDA-MB-468 (right) proliferation assay in the presence of PBS (No treatment), 0.1ng/ml, 1ng/ml and 10ng/ml of CCL8 from 0-80 hr. No statistical differences between treatments and controls ($n = 3$).

ns = not significant, $p < 0.01$, *$p < 0.001$, $p<0.0001$, ***$p<0.00001$; (A-C) One-way ANOVA, (D, E, F, I) Student's t-test, (A, B, C, E, F, H, I) Data depicted as Mean±SEM, (D) Horizontal bars represent the mean of the individual values±SD.

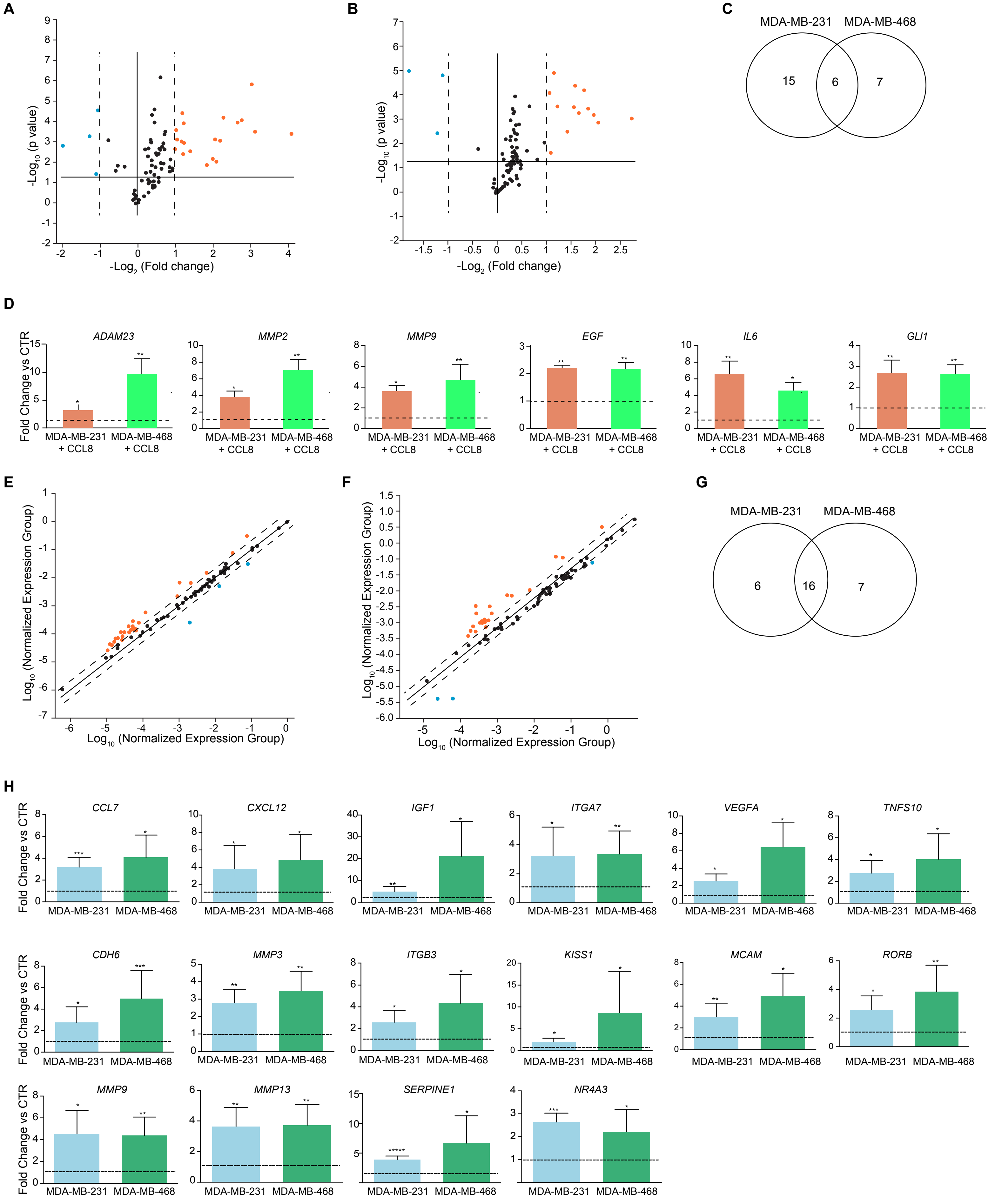
A
B
C
-Log10 (p value)
-Log2 (Fold change)
MDA-MB-231
MDA-MB-468
15
6
7
D
Fold Change vs CTR
ADAM23
MMP2
MMP9
EGF
IL6
GLI1
MDA-MB-231 + CCL8
MDA-MB-468 + CCL8
E
F
G
Log10 (Normalized Expression Group)
MDA-MB-231
MDA-MB-468
6
16
7
H
Fold Change vs CTR
CCL7
CXCL12
IGF1
ITGA7
VEGFA
TNFS10
CDH6
MMP3
ITGB3
KISS1
MCAM
RORB
MMP9
MMP13
SERPINE1
NR4A3
MDA-MB-231
MDA-MB-468

Figure S6. Breast cancer qPCR array on cancer cells stimulated with rCCL8 and macrophage conditioned medium, related to Figure 5.

(A and B) Volcano plot showing genes whose expression is significantly (Log_2FC +/- 1, $p < 0.05$) downregulated (blue dots) and upregulated (orange dots) in MDA-MB-231 (A) or MDA-MB-468 (B) cells after incubation with 1ng/ml of rCCL8 for 16 hr (n = 3).

(C) Venn diagram of commonly upregulated genes between MDA-MB-231 (left circle) and MDA-MB-468 (right circle) after rCCL8 treatment.

(D) mRNA expression of 6 commonly upregulated genes in MDA-MB-231 or MDA-MB-468 after CCL8 stimulation. Dotted black line represents normalized expression level in untreated control samples. Data are depicted as fold change vs CTR (n = 3).

(E and F) Volcano plot showing normalised expression (Log_{10}) of cells after incubation with CM primed MDM supernatant for 16 hr (n = 3) in MDA-MB-231 (E) and MDA-MB-468 (F). Dots represent genes whose expression is significantly ($Log_{10}FC$ +/- 2, $p < 0.05$) downregulated (blue dots) and upregulated (orange dots).

(G) Venn diagram of commonly upregulated genes between MDA-MB-231 (left circle) and MDA-MB-468 (right circle).

(H) mRNA expression of 16 commonly upregulated genes in MDA-MB-231 or MDA-MB-468 after CM primed MDM supernatant stimulation; dotted black line represents normalized expression level in untreated control samples (n = 3).

*$p < 0.01$, **$p < 0.001$, ***$p < 0.0001$, *****$p < 0.000001$; (D, H) Student's t-test, (D, H) Data depicted as Mean±SEM.
